# Supplementary material for: The impact of general practitioners working in or alongside emergency departments: a rapid realist review
Source: BMJ Open. 2019 Apr 11;9(4):e024501. doi: 10.1136/bmjopen-2018-024501 (PMC6500276; doi:10.1136/bmjopen-2018-024501)
Supplement: Supplementary file 1 [file bmjopen-2018-024501supp001.pdf]

Supplementary file 1: Search Strategy for Medline (adapted for other databases)

1. exp Primary Health Care/
2. primary care.mp. [mp=title, abstract, original title, name of substance word, subject heading word, keyword heading word, protocol supplementary concept word, rare disease supplementary concept word, unique identifier, synonyms]
3. exp Physicians, Family/
4. exp Physicians, Primary Care/
5. family physician*.mp. [mp=title, abstract, original title, name of substance word, subject heading word, keyword heading word, protocol supplementary concept word, rare disease supplementary concept word, unique identifier, synonyms]
6. exp Family Practice/
7. family practic*.mp. [mp=title, abstract, original title, name of substance word, subject heading word, keyword heading word, protocol supplementary concept word, rare disease supplementary concept word, unique identifier, synonyms]
8. GP.mp. [mp=title, abstract, original title, name of substance word, subject heading word, keyword heading word, protocol supplementary concept word, rare disease supplementary concept word, unique identifier, synonyms]
9. exp After-Hours Care/
10. (after-hours care or out of hours or out-of-hours or OOH).mp. [mp=title, abstract, original title, name of substance word, subject heading word, keyword heading word, protocol supplementary concept word, rare disease supplementary concept word, unique identifier, synonyms]
11. exp General Practitioners/
12. general practic*.mp. [mp=title, abstract, original title, name of substance word, subject heading word, keyword heading word, protocol supplementary concept word, rare disease supplementary concept word, unique identifier, synonyms]
13. 1 or 2 or 3 or 4 or 5 or 6 or 7 or 8 or 9 or 10 or 11 or 12
14. exp Emergency Medical Services/
15. exp Emergency Service, Hospital/
16. emergency department*.mp. [mp=title, abstract, original title, name of substance word, subject heading word, keyword heading word, protocol supplementary concept word, rare disease supplementary concept word, unique identifier, synonyms]
17. (accident and emergenc*).mp. [mp=title, abstract, original title, name of substance word, subject heading word, keyword heading word, protocol supplementary concept word, rare disease supplementary concept word, unique identifier, synonyms]
18. casualt*.mp. [mp=title, abstract, original title, name of substance word, subject heading word, keyword heading word, protocol supplementary concept word, rare disease supplementary concept word, unique identifier, synonyms]

19. emergency room.mp. [mp=title, abstract, original title, name of substance word, subject heading word, keyword heading word, protocol supplementary concept word, rare disease supplementary concept word, unique identifier, synonyms]

20. A&E.mp. [mp=title, abstract, original title, name of substance word, subject heading word, keyword heading word, protocol supplementary concept word, rare disease supplementary concept word, unique identifier, synonyms]

21. urgent care centre*.mp. [mp=title, abstract, original title, name of substance word, subject heading word, keyword heading word, protocol supplementary concept word, rare disease supplementary concept word, unique identifier, synonyms]

22. (walkin or walk in or walk-in).mp. [mp=title, abstract, original title, name of substance word, subject heading word, keyword heading word, protocol supplementary concept word, rare disease supplementary concept word, unique identifier, synonyms]

23. 14 or 15 or 16 or 17 or 18 or 19 or 20 or 21 or 22

24. 13 and 23

25. limit 24 to yr="2015 -Current"

Supplementary file 2: Articles that contributed data to theory development (listed chronologically by Country)

Lead author	Country	Title	Study Design
Ablard 2017	UK	Primary care services co-located with Emergency Departments across a UK region: early views on their development	Survey and semi-structured interviews
Bentley 2017	UK	Emergency Department redirection to primary care: a prospective evaluation of practice	Prospective evaluation of the subsequent management and outcome of redirected non-urgent patients from a Scottish ED over 2 months
Dale 2017	UK	Extended training to prepare GPs for future workforce needs: a qualitative investigation of a one-year fellowship in urgent care	Qualitative investigation of a one-year fellowship in urgent care
Tammes 2016	UK	Exploring the relationship between general practice characteristics, and attendance at walk-in centres, minor injuries units and EDs in England 2012/2013: a cross-sectional study	Cross-sectional observational large data analysed using multivariable regression models
Proctor 2016	UK	A&E Avoidance schemes across London: A rapid review of good practice examples	NHS report - 2 case studies involving redirection of non-urgent patients from the ED
Smith 2016	UK	To GP or not TO GP: Evaluation of children triaged to see a GP in a tertiary paediatric emergency department	Retrospective cohort study of children classified as 'GP appropriate' seen by a GP between 14:00 and 22:00 and seen by ED staff outside these hours
Gnani 2016	UK	Healthcare use among preschool children attending GP-led urgent care centres: a descriptive observational study	Retrospective observational study using routinely collected data
O'Cathain 2016	UK	Variation in avoidable emergency admissions: multiple case studies of emergency and urgent care systems	Ethnographic residual analysis. Interviews with members of emergency care teams at 6 case study sites

Begum 2016	UK	Solving the A&E crisis using GP lead triage and redirection	Poster - Patient questionnaire of 150 patients over 5 weeks redirected back to the GP for treatment with an appointment made on the same day
Gritz 2016	UK	More under fives now seen in urgent care centre than A&E should we shift our focus	Observational - retrospective analysis of routine operational data for attendances
Greenfield 2016	UK	Staff perceptions on patient motives for attending GP-led urgent care centres in London: a qualitative study	Semi-structured interviews
Cowling 2015	UK	Referral outcomes of attendances at general practitioner-led urgent care centres in London, England: retrospective analysis of hospital administrative data	Retrospective analysis of administrative data recorded at a London urgent care centre of 243042 attendances from October 2009 to December 2012
Morton 2016	UK	Describing team development within a novel GP-led urgent care centre model: a qualitative study	Staff semi-structured interviews at 2 GP-led urgent care centres in 2 London academic teaching hospitals
Arain 2015a	UK	Perceptions of healthcare professionals and managers regarding the effectiveness of GP-led walk-in centres in the UK	Qualitative using a phenomenological approach using semi structured interviews
Arain 2015b	UK	Impact of a GP-led walk-in centre on NHS emergency departments	Patient survey over a 3-week period and analysis of attendances at the local children's hospital and minor injuries unit a year before and after the WIC opened
Johnson 2015	UK	Evidence of primary care services at A&E	Letter (opinion piece) Provider of 4 UCC in London supporting co-located GPs services with emergency departments

NHS ECIST 2015	UK	Primary care in emergency departments: a guide to good practice	NHS policy document - Overview of factors to be considered when planning how best to use primary care clinicians in emergency departments, monitoring and refining the service
Harris 2014	UK	How do clinicians with different training backgrounds manage walk-in patients in the ED setting?	Retrospective case note review of a random sample of 384 patients that self-presented to the ED and were initially assessed by GPs or ED staff
Thompson 2013	UK	Suitability of emergency department attenders to be assessed in primary care: survey of general practitioner agreement in a random sample of triage records analysed in a service evaluation project	Four GPs independently used data extracted from 765 clinical notes to rate the appropriateness for management in primary care
Arain 2013	UK	Patients' experience and satisfaction with GP led walk-in centres in the UK; a cross sectional study.	Patient survey over 3 weeks in 2 GP-led WICs
Hunter 2013	UK	A qualitative study of patient choices in using emergency health care for long-term conditions: The importance of candidacy and recursivity.	Questionnaire and semi-structured interviews
Lengu 2012	UK	Application of simulation and modelling in managing unplanned healthcare demand	Conference paper - Simulation and modelling to assess the impact of primary care clinicians deflecting patients with non-urgent needs away from A&E
Carson 2010	UK	Primary care and emergency departments	Report based on results of a literature review, web-based survey and ED visits
Clancy 2009	UK	Launching a social enterprise see-and-treat service	Report outlining the service, number of patients seen and referred on in a 4-month period
Maheswaren 2009	UK	Repeat attenders at national health service walk in centres	Descriptive study using routine data from 4 walk-in centres in England

Sandhu 2009	UK	Emergency nurse practitioners and doctors consulting with patients in an emergency department: a comparison of communication skills and satisfaction	Observation study with a stratified sample of 296 video-taped consultations
Dale 2008	UK	The patient, the doctor and the emergency department: A cross-sectional study of patient-centeredness in 1990 and 2005	Observational study with a stratified sample of 430 video-taped consultations with data collection in May–July 1990 and May–July 2005.
Salisbury 2007	UK	The impact of co-located NHS walk-in centres on emergency departments	Controlled before and after study
Chalder 2007	UK	Comparing care at walk-in centres and at accident and emergency departments: an exploration of patient choice, preference and satisfaction	A controlled, mixed-method study comparing 8 EDs with co-located WICs with the same number of ''traditional'' EDs.
Pope 2005	UK	What do other local providers think of NHS walk-in centres? Results of a postal survey	Postal survey
Bickerton 2005	UK	Streaming A&E patients to walk-in centre services	Analysis of all patients attending a London hospital over 24 hours for suitability for WIC treatment
Chew-Graham 2004	UK	A new role for the general practitioners? Reframing inappropriate attenders to inappropriate services	Qualitative semi-structured staff interviews
Hsu 2003	UK	Effect of NHS walk-in centre on local primary healthcare services	Before and after observational study of consultation rate in 12 general practices after the implementation of a walk-in centre
Salisbury 2002	UK	What is the role of walk-in centres in the NHS?	Analysis of routinely collected data, questionnaire completed by managers followed by semi-structured interviews and site visits
Grant 2002	UK	An observational study comparing quality of care in walk-in centres with general practice and NHS Direct using standardised patients	Observational study involving assessment of clinicians by standardised patients at 20 walk in centres, 20 general practices ad 11 NHS direct sites

Coleman 2001	UK	Will alternative immediate care services reduce demands for non-urgent treatment at accident and emergency?	Questionnaire survey and notes review of non-urgent patients to assess the suitability of management by an alternative care service
McGugan 2000	UK	Primary care or A&E?	Prospective study over 2 months of a redirection policy
Rajpar 2000	UK	Study of choice between accident and emergency departments and general practice centres for out of hours primary care problems	Interview of patients attending A&E and GP out-of-hours
Freeman 1999	UK	Primary care units in A and E departments in North Thames in the 1990s: Initial experience and future implications	Postal questionnaire to ED staff and local GPs with follow up staff interviews
Dale 1998	UK	Primary care in accident and emergency departments: the cost effectiveness and applicability of a new model of care	PhD thesis – Includes data for included papers and additional analysis of 163 video-taped consultations
Ward 1996	UK	Primary care in London: an evaluation of general practitioners working in an inner-city accident and emergency department	Prospective survey over 6 weeks
Dale 1996	UK	Cost effectiveness of treating primary care patients in accident and emergency: a comparison between GPs, senior house officers and registrars	Prospective intervention study which was retrospectively costed
Dale 1995a	UK	Primary care in the accident and emergency department I: Prospective identification of patients	1 year prospective study at a London ED to compare patient characteristics and consultation activities for attenders assessed by nurse triage to have 'primary care' or 'accident and emergency' type problems
Dale 1995b	UK	Primary care in the accident and emergency department: II. Comparison of general practitioners and hospital doctors	1 year prospective study at a London ED to compare patient characteristics and consultation activities for attenders assessed by nurse triage to have 'primary care' or 'accident and emergency' type problems

O'Kelly 2010	Ireland	Impact of a GP cooperative on lower acuity emergency department attendances	A retrospective review of all attendances at the 'Dubdoc' service was compared with attendances at the ED for triage categories 4 and 5 of the same hospital over a 9-year period
Murphy 2000	Ireland	Effect of patients seeing a general practitioner in accident and emergency on their subsequent attendance: cohort study	Analysis of reattendance of non-urgent patients that had been allocated to general practitioners or usual accident and emergency staff depending on time of registration
Gibney 1999	Ireland	Randomized controlled trial of general practitioner versus usual medical care in a suburban accident and emergency department using an informal triage system	Patients 'randomised' at time of registration to either GP or ED care. Case note review
Murphy 1996	Ireland	Randomised controlled trial of general practitioner versus usual medical care in an urban accident and emergency department: process, outcome and comparative cost	Randomised controlled trial of care provided by general practitioners to non-emergency patients in an accident and emergency department differs significantly from care by usual emergency staff in terms of process, outcome ad cost
van Veelen 2016	Netherlands	Effects of a general practitioner cooperative co-located with an emergency department on patient throughput	Pre-post comparison before and after implementation of a GP cooperative at an ED
Schols 2016	Netherlands	Access to diagnostic tests during GP out-of -hours care: A cross sectional study of all GP out-hours services in the Netherlands	Cross-sectional survey of all 117 GP out of hours services in the Netherlands
Van-Gils-van Rooij 2016	Netherlands	Is patient flow more efficient in urgent care collaborations?	Observational study, compared usual care with UCCs (single point of access for ED and GP OOH)

van Gils-van Rooij 2015	Netherlands	Out-of-Hours Care Collaboration between General Practitioners and Hospital Emergency Departments in the Netherlands	Observational study - comparing attendance and patient characteristics between EDs with standard care and EDs with co-located primary care and single joint triage
Thijssen 2013	Netherlands	The impact on emergency department utilization and patient flows after integrating with a general practitioner cooperative: an observational study	Observational study - routinely collected data over 6 years
Huibers 2013	Netherlands	GP cooperative and emergency department: an exploration of patient flows	Retrospective record review of patients who had visited GPC or ED
Van der Straten 2012	Netherlands	Safety and efficiency of triaging low urgent self-referred patients to a general practitioner at an acute care post: an observational study	Prospective observational study
Bosmans 2012	Netherlands	Addition of a general practitioner to the accident and emergency department: a cost-effective innovation in emergency care	Observational study before and after implementation of new service
Van Veen 2012	Netherlands	Van Veen referral of non-urgent children from the emergency department to general practice: compliance and cost savings	Prospective observational before after study
Van Veen 2011	Netherlands	Safety of the Manchester Triage System to identify less urgent patients in paediatric emergency care: a prospective observational study	Analysis of the hospitalisation rate of self referred children triaged as non-urgent
Boeke 2010	Netherlands	Effectiveness of GPs in accident and emergency departments	Observational study before and after implementation of new service
Kool 2008	Netherlands	Towards integration of general practitioner posts and accident and emergency departments: a case study of two integrated emergency posts in the Netherlands	Observational study comparing contacts, patient satisfaction and staff satisfaction pre-and post set up of a 2 co-located GP OOHs and 2 control sites

Giesen 2006	Netherlands	Patients either contacting a general practice cooperative or accident and emergency department out of hours: a comparison	Retrospective record review
Van Uden 2006	Netherlands	Out-of-hours primary care. Implications of organisation on costs	Annual reports of 2 GP co-operatives (1 co-located, 1 separate) analysed together with ED costs
Van Uden 2005	Netherlands	The Impact of a Primary Care Physician Cooperative on the Caseload of an Emergency Department: The Maastricht Integrated Out-of-Hours Service	Observational study, patient characteristics collected for 3 weeks in Jan/Fen 1998 and March 2001 (co-operative set up in 2000)
Van Uden 2004	Netherlands	Does setting up out of hours primary care cooperatives outside a hospital reduce demand for emergency care?	Before and after observational study
Van Uden 2003	Netherlands	Use of out of hours services: a comparison between two organisations	Observational study of patient contacts at 2 different OOH centres and their associated EDs (1 co-located, 1 not)
Colliers 2017	Belgium	Implementation of a general practitioner cooperative adjacent to the emergency department of a hospital increases the caseload for the GPC but not for the emergency department	Quasi-experimental study analysing the implementation of 2 out of hours general practitioner co-operatives one adjacent to the ED, the other not and 2 control sites
Van den Heede 2016	Belgium	The 2016 proposal for the reorganisation of urgent care provision in Belgium: A political struggle to co-locate primary care providers and emergency departments	Outline of the 2016 political proposal for the reorganisation of urgent care provision toned down due to GP opposition
Ellbrant 2015	Sweden	Paediatric emergency department management benefits from appropriate early redirection of non-urgent visits	Prospective observational study using ED records and case notes
Krakau 1999	Sweden	Provision for clinic patients in the ED produces more nonemergency visits	Before and after observational study

Hansagi 1987	Sweden	Trial of a method of reducing inappropriate demands on a hospital emergency department.	Prospective observational study of 454 patients classified as non-urgent by the ED and redirected to alternative care over a 3-month period
Chmiel 2016	Switzerland	Implementation of a hospital-integrated general practice – a successful way to reduce the burden of inappropriate emergency-department use	Longitudinal observational study
Hess 2015	Switzerland	Satisfaction of health professionals after implementation of a primary care hospital emergency centre in Switzerland: A prospective before-after study	Questionnaire study of job satisfaction before and after a new emergency care model was implemented in Switzerland
Wang 2014	Switzerland	Hospital integrated general practice: a promising way to manage walk in patients	Pre and post comparison study before and after implementation of a new hospital-integrated general practice model
Chmiel 2011	Switzerland	Walk-ins seeking treatment at an emergency department or general practitioner out-of-hours service: a cross-sectional comparison	Analysis of routinely collected data of 2974 patient encounters attending a GPC or ED
Posocco 2017	Italy	Role of out of hours primary care service in limiting inappropriate access to emergency department	Retrospective analysis of 408 ED referrals from a local OOH service
Kork 2016	Finland	Improving access and managing healthcare demand with walk in clinic: convenient but at what cost?	Observational study over 48 months of the characteristics of 107 frequent attenders at a WIC from electronic patient records
Allen 2015	Australia	Low acuity and general practice type presentations to emergency departments: A rural perspective	Analysis of GP type presentations to 2 rural EDs over a 4-month period
Desborough 2013	Australia	Development and implementation of a nurse-led walk-in centre: evidence lost in translation?	Evaluation of the first 12 months of operation of the first Australian public nurse-led primary care walk-in centre compared to the English NHS model.

Nagree 2013	Australia	Quantifying the proportion of general practice and low-acuity patients in the emergency department	Four methods for calculating general practice-type patients were compared for 3 tertiary EDs in Perth, Australia in 2009-2011
Sharma 2011	Australia	Impact of co-located general practitioner (GP) clinics and patient choice on duration of wait in the emergency department	Mathematical modelling of wait times using routine ED data
Richardson 2009	Australia	Myths versus facts in emergency department overcrowding and hospital access block.	Report referencing previous work
Bolton 2001	Australia	The reasons for, and lessons learned from, the closure of the Canterbury GP After-Hours Service.	Report describing why a 12-month trial of GP staffed after hours service with an ED was not continued because the opportunity cost was greater than existing alternative services
Doran 2013	USA	An intervention connecting low acuity emergency department patients with primary care: Effect on future primary care linkage	Analysis of primary care follow up of patients presenting to ED assessed to have non-urgent problem and referred to an onsite primary care clinic
Williams 1996	USA	The costs of visits to emergency departments.	Analysis of emergency department charges and costs based on data from 6 community hospitals
Gadomski 1995	USA	Diverting managed care Medicaid patients from pediatric emergency department use.	6-month follow up of Medicaid children with non-emergent conditions not authorised to be seen in the Pediatric Emergency Department by their primary care provider
Derlet 1995	USA	Prospective identification and triage of nonemergency patients out of an emergency department - 5 year study	5 year study to analyse the outcome of adult patients refused care in the ED
Derlet 1992	USA	Triage of patients out of the emergency department: three year experience.	3 year study to analyse the outcome of adult patients refused care in the ED

Birnbaum 1994	USA	Failure to validate a predictive model for refusal of care to emergency-department patients.	Analysis of the outcome of 534 patients that met the pre-established criteria for refusal of care
Lowe 1994	USA	Refusing care to emergency department patients: evaluation of published triage guidelines.	Case note review of 106 patients who would have been refused care according to triage guidelines
Shaw 1990	USA	Indigent children who are denied care in the emergency department.	Six-month prospective study of 588 children denied care in the emergency department
Rivara 1986	USA	Pediatric nurse triage: its efficacy, safety and implications for care.	Evaluation of emergency room triage of 748 children over a 6-week period at a large urban children's hospital that routinely referred outside of the institution for care
Schull 2007	Canada	The Effect of Low-Complexity Patients on Emergency Department Waiting Times	Analysis of 4.1 million patient visits over a 1 year period (2002-3) and 110 EDs of the effect of low-complexity patients on time of physician contact of high complexity patients
Vertesi 2004	Canada	Does the Canadian Emergency Department Triage and Acuity Scale identify non-urgent patients who can be triaged away from the emergency department?	Retrospective database audit in an urban referral hospital ED.
Hutchison 2003	Canada	Patient satisfaction and quality of care in walk-in clinics, family practices and emergency departments: the Ontario Walk-In Clinic Study.	Prospective cohort study of the quality of care of 8 common acute conditions and patient satisfaction
Anantharaman 2008	Singapore	Impact of health care system interventions on emergency department utilisation and overcrowding in Singapore	Retrospective analysis of attendances at six main public EDs over 32 years
Wilson 2005	New Zealand	Co-locating primary care facilities within emergency departments: brilliant innovation or unwelcome intervention into clinical care?	Report reviewing a proposal to co-locate a primary care facility within the local emergency department
