# Supplementary material for: The impact of general practitioners working in or alongside emergency departments: a rapid realist review
Source: BMJ Open. 2019 Apr 11;9(4):e024501. doi: 10.1136/bmjopen-2018-024501 (PMC6500276; doi:10.1136/bmjopen-2018-024501)
Supplement: Author's manuscript [file bmjopen-2018-024501.draft_revisions.pdf]

BMJ Open is committed to open peer review. As part of this commitment we make the peer review history of every article we publish publicly available.

When an article is published we post the peer reviewers' comments and the authors' responses online. We also post the versions of the paper that were used during peer review. These are the versions that the peer review comments apply to.

The versions of the paper that follow are the versions that were submitted during the peer review process. They are not the versions of record or the final published versions. They should not be cited or distributed as the published version of this manuscript.

BMJ Open is an open access journal and the full, final, typeset and author-corrected version of record of the manuscript is available on our site with no access controls, subscription charges or pay-per-view fees (http://bmjopen.bmj.com).

If you have any questions on BMJ Open's open peer review process please email editorial.bmjopen@bmj.com

BMJ Open

The effectiveness of primary care service models in or alongside emergency departments: a rapid realist review

Journal:	*BMJ Open*
Manuscript ID	bmjopen-2018-024501
Article Type:	Research
Date Submitted by the Author:	12-Jun-2018

Complete List of Authors:	Cooper, Alison; Cardiff University, Division of Population Medicine<br>Davies, Freya; Cardiff University, Division of Population Medicine<br>Edwards, Michelle; Cardiff University, Division of Population Medicine<br>Anderson, Pippa; Swansea University, Centre for Health Economics<br>Carson-Stevens, Andrew; Cardiff University, Division of Population Medicine<br>Cooke, Matthew; University of Warwick Warwick Medical School<br>Donaldson, Liam; London School of Hygiene and Tropical Medicine<br>Dale, Jeremy; University of Warwick, Warwick Medical School<br>Evans, Bridie; Swansea University, Medicine<br>Hibbert, Peter; Macquarie University Faculty of Medicine and Health Sciences, Faculty of Medicine and Health Sciences; University of South Australia Division of Health Sciences,<br>Hughes, Thomas; John Radcliffe Hospital, Emergency Department<br>Porter, Alison; University of Swansea, College of Medicine<br>Rainer, Tim; Cardiff University, Division of Population Medicine<br>Siriwardena, Aloysius; University of Lincoln, School of Health and Social Care<br>Snooks, Helen; Swansea University<br>Edwards, Adrian; Cardiff University, Division of Population Medicine

Keywords:	Emergency Service, Hospital, Primary Health Care, General practitioners, Health Services Research

SCHOLARONE™ Manuscripts

The effectiveness of primary care service models in or alongside emergency departments: a rapid realist review

Cooper, A, Davies F, Edwards, M, Anderson, P, Carson-Stevens, A, Cooke, M, Donaldson, L, Dale J, Evans, B, Hibbert, P, Hughes, T, Porter, A, Rainer, T, Siriwardena, A, Snooks, H, Edwards, A

Corresponding author: Dr Alison Cooper, Division of Population Medicine, Cardiff University, 5th Floor Neuadd Meirionnydd, Heath Park, Cardiff CF14 4YS
CooperA8@cardiff.ac.uk 02920 870746

Dr Alison Cooper, Cardiff University, Cardiff, UK
Dr Freya Davies, Cardiff University, Cardiff, UK
Dr Michelle Edwards, Cardiff University, Cardiff, UK
A/Prof Pippa Anderson, Swansea University, Swansea, UK
Dr Andrew Carson-Stevens, Cardiff University, Cardiff, UK
Prof Matthew Cooke, Warwick University, Coventry, UK
Prof Sir Liam Donaldson, London School of Hygiene and Tropical Medicine. London, UK
Prof Jeremy Dale, Warwick University, Coventry, UK
Dr Bridie Evans, Swansea University, Swansea, UK
Mr Peter Hibbert, Macquarie University, Sydney, Australia
Dr Thomas Hughes, John Radcliffe Hospital, Oxford, UK
A/Prof Alison Porter, Swansea University, Swansea, UK
Prof Tim Rainer, Cardiff University, Cardiff, UK
Prof Aloysius Siriwardena, Lincoln University, UK
Prof Helen Snooks, Swansea University, Swansea, UK
Prof Adrian Edwards, Cardiff University, Cardiff, UK

Keywords: Emergency Service, Hospital; Primary Health Care; General practitioners; Health Services Research

Word count: 4210

Abstract

Objectives

Worldwide, emergency healthcare systems are under intense pressure from ever-increasing demand and evidence is urgently needed to understand how this can be safely managed. An estimated 10-43% of emergency department patients could be treated by primary care services. In England, this has led to a policy proposal and £100million of funding ($140million USD), for all emergency departments to stream appropriate patients to a co-

For peer review only

located primary care facility. However, the research evidence to support this initiative is weak. Our aim was to explain how and why general practitioners working in or alongside emergency departments affect: patient flow, patient experience, patient safety and the wider healthcare system

Design

Rapid realist literature review

Setting

Emergency departments

Intervention

General practitioners working in or alongside

Inclusion criteria

Articles contributing data to theory development

Primary outcome measure

Context-specific theories that explain how and why the intervention works and policy implications

Results

Ninety-six articles contributed data to theory development sourced from earlier systematic reviews, updated database searches (Medline, Embase, CINAHL, Cochrane DSR & CRCT, DARE, HTA Database, BSC, PsycINFO and SCOPUS), and citation tracking. We developed theories to explain: how staff interpret the streaming system, different roles general practitioners adopt in the emergency department setting (traditional, extended, gatekeeper or emergency clinician), and how these factors influence patient (experience and safety) and organisational (demand and cost-effectiveness) outcomes.

Conclusions

For peer review only

Multiple factors influence the effectiveness of emergency department streaming to primary care services; caution is needed in embedding the policy until further evaluation is available. Service models that encourage the traditional general practitioner approach may have shorter process times; however, there is little evidence on the safety implications or whether this improves care for the sickest patients. Distinct primary care services offering increased patient choice may result in provider-induced demand. Economic evaluation requires further research.

Trial registration

Prospero ID=CRD42017069741

300/300

Strengths and limitations of this study

- A realist approach to evidence synthesis leads to theory development that explains how and why context links to outcome
- Contextual factors can then be incorporated into the evidence base to inform healthcare management and policy making
- We utilised experts and stakeholders to streamline the process and produce a context-specific product in response to emerging issues
- We sought to inform United Kingdom policy regarding emergency department provision which limits transferability of results
- Some studies did not describe how the intervention operated in adequate depth to identify key mechanisms that led to the outcomes

For peer review only

The effectiveness of primary care service models in or alongside emergency departments: a rapid realist review

Background

Emergency healthcare systems, in most high-income countries, are under intense pressure from ever increasing demand. This is particularly so at colder times of year as an ageing and frail population is precipitated into crisis by acute illness.(1) Evidence is needed on how best to manage this demand whilst safely achieving the highest standards of care.(2) An estimated 10-43% of patients attending hospital emergency departments could be treated in primary care settings.(3–9) In the United Kingdom, this has led to a policy proposal, supported by £100million of funding ($140million USD), that all emergency departments in England have a co-located primary care facility, so they are "free to care for the sickest patients". (10–12)

The United Kingdom has a universal healthcare system, the National Health Service, funded though taxation.(13) Primary care is led by general practitioners, community-based doctors with generalist training. Three primary care service models associated with emergency departments are described: a) treatment in a unit alongside the emergency department; b) general practitioners redirecting patients with primary care problems out of the emergency department to the most appropriate healthcare setting; and c) general practitioners fully integrated into the hierarchy of emergency department provision.(14) There is little research evidence to guide decisions about which of these service models is most effective. The risk of provider-induced demand, potential patient safety issues and how to recruit a workforce for this initiative are also unclear. (15–19) Due to this uncertainty, the main standard-setting body in the National Health Service (NICE) does not currently recommend general practitioners work in emergency department settings.(20)

For peer review only

Research studies addressing these questions are heterogeneous and few are conducted at scale.(15–17) This limits the results of traditional synthesis methods required to shape practice or policy. Realist methods offer an alternative approach, generating theories to explain why a particular intervention is likely to work, how, for whom, in what circumstances, and why.(21) These methods incorporate contextual factors into the evidence base to inform healthcare management and policy making.(22) Urgent and emergency care settings vary in geographical location, the type of patients, the presenting conditions and the experience and disciplines of the healthcare clinicians that treat them. We decided that a realist approach, aiming to explain how general practitioners work in or alongside different emergency department settings and why the resultant successes or failures occur, would be more informative than a traditional review approach.

Our research question was, “Why and how do general practitioners working in or alongside emergency departments affect: patient attendance and flow; patient experience; patient safety; and the wider healthcare system?”

For peer review only

Box 1: Glossary of terms(21,23)

Primary care problem	A condition that a typical general practitioner in a typical general practice would be expected to manage
Streaming	A system to allocate patients to the most appropriate healthcare provider within the emergency department setting
Triage	A system to identify seriously unwell patients to prioritise treatment
Context (C)	Pre-existing conditions which influence the success or failure of different interventions or programmes.
Mechanism (M)	The intervention and people's reaction to it; how does it influence their reasoning?
Outcome (O)	Intended and unintended results as a result of a mechanism operating within a context
Initial theory	An early theory informed by available evidence describing why, how and for whom the intervention is thought to work using a context-mechanism-outcome configuration
Refined theory	An initial theory that has been refined using primary or secondary evidence

Method

We followed the realist review methodology to identify mechanisms (M) that explain how or why contexts (C) relate to outcomes (O), to generate *theories* described as context-mechanism-outcome configurations.(21) (Specific terminology is defined in Box 1.) Our focus was specifically on general practitioners working in or alongside emergency departments. We used the rapid realist review approach described by Saul et al. which utilises experts and stakeholders to streamline the process and to produce a context-specific product that is useful to policy makers and responsive to emerging issues; providing evidence and making explicit what is known on the given topic, also articulating the current research gaps.(24) We registered our protocol on the Prospero database (http://www.crd.york.ac.uk/PROSPERO/display_record.php?ID=CRD42017069741) and followed RAMESES publication standards for realist reviews.(25) The period of study was April – November 2017.

For peer review only

Three reviewers (AC, FD and ME) conducted a scoping exercise with the four United Kingdom papers identified in the review by Ramlakhan et al.(4,26–28) and two policy documents,(14,29) to generate *initial theories*. We then developed and piloted data extraction forms. Our theories were developed at the *micro-level* (the reasoning processes of general practitioners, emergency department staff and patients), *meso-level* (staff interactions resulting in department level outcomes) and *macro-level* (the impact on the wider system).(30)

We discussed these initial theories with the wider study team of 18 collaborators, including emergency department clinicians, policy makers, general practitioners, members of the public and methodologists at a study meeting in May 2018. We used them as an expert reference group, to contribute ideas for other possible initial theories and to identify further research papers in peer-reviewed journals and relevant reports in the grey literature. Six members of this group, including two (AP, PA, BE, BH, JD, ACS) met via teleconference every six weeks to discuss findings and guide priority search areas.

We used papers referenced in three previous systematic reviews as a starting point.(15–17) To identify papers published since, we combined search terms used previously.(16,17) A combination of free text and Medical Subject Headings (MeSH) terms was used (see supplementary file 1 for Medline strategy which was adapted for other databases). AC ran the searches on the following databases from 15th June – 4th July 2017: Medline via OVID, Embase, CINAHL, Cochrane DSR & CRCT, DARE, HTA Database, Business Source Complete, PsycINFO and SCOPUS and used Endnote X8 (Clarivate analytics) to export citations from the database searches and identify duplicates. AC screened the titles and abstracts of all identified papers using a checklist, developed and tested in collaboration with FD, which ranked abstracts according to relevance.

For peer review only - http://bmjopen.bmj.com/site/about/guidelines.xhtml

We selected studies if they could contribute to the process of theory development at the level of individual data extracts rather than assessing the full text against a set checklist.(25,31) We excluded papers that lacked relevance or explanatory power, or were unavailable in English. AC and FD imported data extracts into NVivo 11 (QRS international) that evidenced mechanisms (M) to explain how or why contexts (C) related to outcomes (O). Quantitative, qualitative or contextual data were extracted from any part of a paper. We continually considered the relevance and rigour of each included piece of evidence during the data extraction and synthesis phases.(31) We discussed weekly within the team (AC, FD, ME, AE) how individual data extracts should be used to ensure appropriate inferences were made.(31) A quarter of all included articles was read by both reviewers, and the coding process was discussed in detail, to ensure consistency of approach.

We used snowballing techniques (such as searching companion papers and citation tracking) for all included articles. We also searched to identify additional relevant grey literature (including policy documents and opinion pieces) from a variety of sources. The search process was iterative, overlapping with data extraction and analysis, and was directed towards the evidence gaps and finding explanatory information.

We applied Pawson's reasoning processes,(21) to synthesise the evidence and develop our theories. We presented these context-specific *developing theories* to our expert reference group in November 2017. At this stage, the group recognised that although the review had been useful in theory development, there were limited opportunities for theory testing and refinement due to evidence gaps. Rather than continuing to search the literature for additional secondary data, we decided that gathering primary data from our evaluation case study sites in the next phase of our wider ongoing study,(32) would give more meaningful testing to derive *refined theories* .(21)

Patient and public involvement

Three public contributors (BE, BH, JH) were co-applicants for the funded research and contributed to the conceptualisation of our wider study, including theory generation through the review.(32) They contributed in both meetings described above to ensure that the patient's perspective was acknowledged and at a stakeholder dissemination event in February 2018.

Results

Figure 1 shows the search strategy and results, a total of 96 articles contributed to the developing theories. The articles were largely primary research studies and involved patients with primary care problems, directed to general practitioners for treatment. Most articles were from the United Kingdom (n= 44 articles), with a large contribution from The Netherlands (n=17). Others were from Ireland, Belgium, Switzerland, Sweden, Italy, Finland, Australia, USA, Canada, Singapore and New Zealand.

We synthesised data to develop theories, described using Context (C) - Mechanism (M) - Outcome (O) configurations, to explain: how or why emergency department staff and general practitioners react to guidance to determine which patients are streamed to general practitioners; the role general practitioners may adopt in the emergency department setting (traditional general practitioner, extended general practitioner, gatekeeper or emergency clinician); and how these factors influence patient (experience and safety) and organisational (risk of provider-induced demand and cost-effectiveness) outcomes. These theories are summarised in Table 1 with an indication of supporting data, and how they link is shown in Figure 2. Full details of included articles are listed in supplementary file 2.

For peer review only - http://bmjopen.bmj.com/site/about/guidelines.xhtml

Table 1: Summary of *developing theories* and supporting evidence

Theory	Context (C) – Mechanism (M) – Outcome (O) Configuration	Example of supporting extract	Evidence base
1. Effectiveness of the streaming system	General practitioners and emergency department staff use their own personal experience and expectation (C) when interpreting streaming guidance (M) to influence which patients are streamed to general practitioners (O).	*"We've been underperforming really, from the amount we potentially thought we might be able to see and the number of patients we're actually seeing ... if you actually talk to the GPs, they're actually saying the cohort of patients that they're getting through are not suitable because they're minor injuries and we're not trained in minor injuries."* (Consultant) (33)	Data to support theory (4,14,40,41,26,33–39)
2a. Traditional general practitioner role vs emergency clinician role	When general practitioners working in the emergency department maintain a 'traditional role' using the same approach taken in the primary care setting (M) to treat patients with primary care problems (C), investigations, admissions and process times will reduce (O). However, if general practitioners adopt an 'emergency clinician role' working as another pair of hands ('going native') because of their personal interest or experience or because they feel this is the correct way to work in this setting (M), there will be no difference in the rate of investigations and admissions (O).	*"I guess our emergency medicine approach is we're looking for something dreadful and a GP approach is very different in that most of the time they know it's minor stuff or … moderate stuff that is self-limiting and so … they're looking to find symptomatic relief and how can we get this patient home and away from hospital."* (Consultant)(33)<br>*"Once they start becoming like everyone else then they stop being like a GP and they don't necessarily work quickly and effectively which is supposed to be the whole benefit of having them there."* (Consultant)(33)	Data to support traditional GP role theory (4,5,46–49,26,27,33,38,42–45)<br><br>Limited data to support ED clinician role theory(33,50)
2b. Extended general practitioner role	General practitioners in emergency departments can work in an 'extended role', outside the scope of usual practice, where their skills are directed at specific patient groups including non-urgent paediatric or elderly patients (C), to treat using the usual primary care approach (M), to reduce the use of hospital resources and admissions in these patient groups (O).	*"During a 6-month pilot scheme which co-located a primary care GP service in a busy paediatric ED, patients seen during the hours when the GP was available were significantly less likely to be admitted, exceed the 4-hour waiting target or leave before being seen, but more likely to receive antibiotics."(5)*	Data to support theory for paediatric patients only (5,29,51–53)

For peer review only - http://bmjopen.bmj.com/site/about/guidelines.xhtml

2c. Gatekeeper role	General practitioners can use their generalist skills and knowledge of community resources (M) to redirect patients presenting with primary care problems (C) back into the community for treatment thereby reducing emergency department attendances (O).	*"GPs and nurses based in triage identify patients who could be managed more appropriately in primary care as soon as they enter the Emergency Department, and re-direct them back to primary care services."*(54)	Limited data to support theory (54,55)
3. Patient satisfaction	Patients with primary care problems that present to emergency departments (C) and are seen by general practitioners, are more satisfied with the care they receive (O) if the experience exceeds expectation (M), but if they do not perceive any difference in the care they received compared to what they expected (M), there is no difference in satisfaction (O).	*"There were no significant differences in (patient) satisfaction ratings between the three groups of doctors (GPs/SHOs/Registrars)"*(38)	Limited data to support theory (27,38,42,44,56–60)
4. Safety implications	Patients with primary care problems that present to emergency departments (C) can be appropriately and safely managed by general practitioners (M) so that emergency department staff are free to appropriately and safely manage seriously unwell patients (O).	*"The attribution of overcrowding in ED to attendance by GP-type patients is simplistic; it does not address how patients are processed within ED or how they are transferred to wards later if required ('access block')."*(43)	No data to support, some opposing data
5. Risk of provider-induced demand	If patients with primary care type problems present to emergency departments (C) and are streamed to indistinct primary care services, without patient awareness or choice (M), there is no provider-induced demand (O). However, distinct urgent primary care services may offer convenient access to primary care (M), resulting in provider-induced demand (O).	*"A and E [ED] has not seen any reduction in their patients. If there is a service, patient[s] will use it. You could have three walk-in centres in the city and all three would be used and you may still not see any dropping in A and E [ED] counts."* (Manager)(61)	Data to support theory (4,28,69–72,61–68)
6. Cost-effectiveness	If there is demand for patients with primary care problems presenting to emergency departments (C), and they are streamed to an onsite primary care service and managed by a traditional general practitioner approach (M), the service is cost-effective due to fewer referrals, admissions, investigations and better outcomes compared to usual services (O).	*"Management of patients with primary care needs in accident and emergency department by general practitioners reduced costs with no apparent detrimental effect on outcome."(27)*	Limited data to support theory (27,42,48)

For peer review only - http://bmjopen.bmj.com/site/about/guidelines.xhtml

Theory 1: Effectiveness of the streaming system

General practitioners and emergency department staff use their own personal experience and expectation (C) when interpreting streaming guidance (M) to influence which patients are streamed to general practitioners (O).(4,14,40,41,26,33–39)

Twelve articles supported this theory and indicated how the streaming process itself directly influenced the effectiveness of the general practitioner service in the department. Variable streaming rates were described due to the guidance itself and also how the guidance was interpreted by emergency department staff.(33,37,39,40) The triage nurse was sometimes described as being unclear which patients general practitioners could deal with,(4,26,33–37) or being more familiar with emergency department work so favouring emergency department referral,(14,33,35,37,38,41) even overruling the guidelines if he/she felt that the patient would require specific investigations,(35) or admission.(41) General practitioners were also noted to override nurse triage decisions to select patients that suited their own interests or perceived skills. (73) Increased referral rates were reported when there was a good relationship between the general practitioners and emergency department nurses.(39) General practitioners were directly involved in the streaming process in two studies resulting in high general practitioner referral rates.(52,74) The influence of commissioning or leadership was not described.

Theory 2a: Traditional general practitioner role vs emergency clinician role

When general practitioners working in the emergency department maintain a 'traditional role' using the same approach taken in the primary care setting (M) to treat patients with primary care problems (C),(33,38,42–45) *investigations, admissions and process times will reduce (O).*(4,5,49,26,27,42,44–48) *However, if general practitioners adopt an 'emergency*

For peer review only - http://bmjopen.bmj.com/site/about/guidelines.xhtml

clinician role' working as another pair of hands ('going native') because of their personal interest or experience or because they feel this is the correct way to work in this setting (M), there will be no difference in the rate of investigations and admissions (O).(33,50)

The traditional general practitioner approach was described by many authors as a different approach to risk management and diagnostic uncertainty, with less reliance on acute investigations.(33,38,43–45) This approach was maintained in a variety of different settings despite full access to investigations – when general practitioners were allocated a separate consulting room mimicking usual general practice,(4,26) and also when general practitioners worked in a more fully integrated model, alongside emergency department clinicians.(42,44,45) Other articles supported general practitioners managing non-urgent patients in this way to divert attendances from emergency department staff. (36,37,76–83,40,52,53,62,63,68,74,75)

There were limited qualitative data to support the 'emergency clinician role' theory.(33) An Irish study described an "unstructured receptionist-based triage system" for all patients attending the department (including referrals from primary care) which may have influenced these relatively inexperienced general practitioners to adopt this role and rely more on diagnostic tests.(50) The influence of general practitioners' special interests, experience in emergency medicine or the effect of staff shortages were not described in the literature to affect this potential role shift.

Theory 2b: Extended general practitioner role

General practitioners in emergency departments can work in an 'extended role', outside the scope of usual practice, where their skills are directed at specific patient groups including

For peer review only

non-urgent paediatric or elderly patients (C) to treat using the usual primary care approach (M) to reduce the use of hospital resources and admissions in these patient groups (O).(5,29,51–53)

Several paediatric primary studies supported general practitioners treating children triaged as 'non-urgent' to divert attendances from the emergency department,(52,53) and reduce hospital admissions.(5,51) None of the included primary studies described general practitioners specifically treating care home residents or the elderly, as suggested in a policy document.(29)

Smith et al. reported an increase in antibiotic prescribing for children by general practitioners,(5) which could potentially be an unintended consequence of the 'traditional role' approach, relying on clinical acumen and treating a suspected source of infection rather than admitting, investigating and observing the patient to confirm the diagnosis. An increase in prescribing by general practitioners was not described in other United Kingdom studies,(4,26) but was reported (but not the drugs involved) in both Irish studies that involved more junior general practitioners.(42,50)

There was evidence that general practitioners working in or alongside emergency departments see a different cohort of patients to that in usual general practice, with more acutely unwell patients,(33,84) and minor injuries,(4,6,33,35–37,85,86) which could also be described as an 'extended role.' There was no evidence in the included studies for the implications of this on their skillset, learning needs, cognition processes or risk management behaviour.

For peer review only - http://bmjopen.bmj.com/site/about/guidelines.xhtml

Theory 2c: Gatekeeper role

General practitioners can use their generalist skills and knowledge of community resources (M) to redirect patients presenting with primary care problems (C) back into the community for treatment thereby reducing emergency department attendances (O). (54,55)

There were limited data to support this theory with two London case study reports identified in an "A&E avoidance" scheme document, describing 228 patients in total.(54,87) There was evidence that general practitioners were more likely to redirect patients after an initial assessment than senior emergency department nurses, but only from a sample of 384 patients that self-presented to a London emergency department.(55)

Due to a lack of evidence for general practitioners performing a redirection role, following realist methodology, we also included studies involving redirection of patients from the emergency department by a senior emergency department clinician or nurse to gain understanding about how and why the system worked. Many of these articles described reduced emergency department attendances.(88–94) Previous United Kingdom guidance has cautioned redirecting patients from emergency departments due to the risk of delayed assessment and treatment, especially in vulnerable patient groups including the homeless or those with mental health problems who may not go on to receive the care they need.(14,29) Studies from Scotland, Sweden and the United States that described a comprehensive assessment process involving measurement of vital signs and a focussed history, reported that their redirection policies were safe and worked well to reduce attendances.(66,89,91,93,94) Other United States studies, that did not describe the assessment process, reported adverse events when children were redirected without treatment.(95,96) The low sensitivity of triage criteria to identify those that needed urgent

For peer review only - http://bmjopen.bmj.com/site/about/guidelines.xhtml

care,(97) especially infants,(98) and failure to validate a predictive model for refusal of care,(99) were highlighted in other studies.

Theory 3: Patient satisfaction

Patients with primary care problems that present to emergency departments (C) and are seen by general practitioners, are more satisfied with the care they receive (O) if the experience exceeds expectation (M), but if they do not perceive any difference in the care they received compared to what they expected (M), there is no difference in satisfaction (O).(27,38,42,44,56–60)

Data to support this theory were limited, with an increase in satisfaction by patients seen by general practitioners generally associated with shorter waiting times,(44,56) rather than expectation of investigation and treatment.(38) The general practitioners were sometimes supernumerary which may have contributed towards this.(27,44) Other studies demonstrated that general practitioners focused more on patient education and counselling than emergency department clinicians with some improvement in satisfaction rates.(100,101) In more fully integrated models, the patient was often unaware that they had seen a general practitioner rather than an emergency department clinician and there was no difference in patient satisfaction.(27,38,42,57)

Theory 4: Safety implications

Patients with primary care problems that present to emergency departments (C) can be appropriately and safely managed by general practitioners (M), so that emergency department staff are free to appropriately and safely manage seriously unwell patients (O).

For peer review only - http://bmjopen.bmj.com/site/about/guidelines.xhtml

There were minimal data on the safety implications of general practitioners working in the emergency department setting. Several studies used emergency department re-attendance as a marker of safety, with no increase among patients seen by general practitioners compared to usual emergency department staff. (27,28,42,102,103) Annual death rates were used as another crude marker in a Dutch study, with no significant increase following the introduction of an out-of-hours primary care physician cooperative.(63) Shared or separate governance systems between primary care and the emergency department were rarely described in the primary studies, providing no evidence for best practice. Single entry co-located emergency department models were reported to promote good communication and integration in some studies,(4,27,33,84) with anecdotal reports of communication positively and negatively affecting care quality in others.(33,53)

There was a lack of evidence that co-located primary care services directly or indirectly improved care for the sickest patients. A reduction in time spent in the department for patients requiring emergency department level care was suggested in a United Kingdom simulation and modelling study,(104) and an Australian study also reported a reduced mean time taken to see more seriously ill patients but this was not seen on sites that described provider-induced demand.(105) A Canadian study of over 4 million patient visits reported that low complexity emergency department patients did not increase time to first physician contact for high-complexity patients.(106) Other studies also described how diverting non-urgent patients did not improve the high level care required by others, and that influences such as delayed transfer of patients to the ward were more likely to contribute to overcrowding.(43,107–109) Staffing levels, staff attitude and the time of day were independent factors described to affect emergency department flow.(110)

For peer review only - http://bmjopen.bmj.com/site/about/guidelines.xhtml

Theory 5: The risk of provider-induced demand

If patients with primary care type problems present to emergency departments (C) and are streamed to indistinct primary care services, without patient awareness or choice (M), there is no provider-induced demand (O).(4,28,62,63) *However, distinct urgent primary care services may offer convenient access to primary care (M), resulting in provider-induced demand (O).*(61,64–72)

Four articles described fully integrated models, where non-urgent patients were streamed directly to primary care services without provider-induced demand.(4,28,62,63). Here, there was no patient choice offered and often a lack of patient awareness. Another 10 articles described distinct urgent primary care services as duplicating services and creating their own demand, increasing patient presentation rates directly or at nearby services, rather than relieving pressure on the emergency department.(61,64,112,113,65–67,69–72,111)

Theory 6: Cost-effectiveness

If there is demand for patients with primary care problems presenting to emergency departments (C), and they are streamed to an onsite primary care services and managed by a traditional general practitioner approach (M), the service is cost-effective due to fewer referrals, admissions, investigations and better outcomes compared to usual services (O).(27,42,48)

Data to support this theory were limited, but supported by three economic evaluations (UK, Ireland and The Netherlands) where non-urgent patients were streamed to general practitioners during normal daytime hours.(27,42,48) The comparator was ‘business as usual’ with no general practitioner service. The United Kingdom and Irish studies were

For peer review only - http://bmjopen.bmj.com/site/about/guidelines.xhtml

published in 1996 and may not represent current emergency department staffing models. No articles were identified that studied the relative cost-effectiveness of general practitioners redirecting patients from the emergency department for care elsewhere. A 5-year USA redirection study calculated cost-effectiveness from the perspective of the institution but did not include costs for treatment incurred elsewhere,(91) whilst another United States study calculated that marginal costs for non-urgent visits to the emergency department were low and that cost savings from diverting visits may be less than widely believed.(114) However the USA has a complex health system, with a significant majority of the population covered by private health insurance alongside state funded Medicare, Medicaid, the Federally-funded Veterans Health Administration, and a substantial uninsured population - all factors which could influence access to emergency departments and the type of care needed and delivered.

Three other studies of 'out-of-hours' patients did not find the addition of a primary care service to be cost saving. One Dutch study, with an off-site general practitioner co-operative, reported parents refusing to take the child to a different location, or the triage nurse overruling the policy.(41) Another 12 year old Dutch study showed no change in costs, despite a substantial reduction in emergency department attendances, due to regulations dictating minimum staffing levels to cope with major trauma.(75) The Dutch health care system has a complex funding structure with a mix of social and private insurance and this may influence incentives and disincentives to access emergency departments. An Australian primary care out-of-hours service closed because patients chose to attend an equally accessible general practice service that existed nearby.(39)

For peer review only - http://bmjopen.bmj.com/site/about/guidelines.xhtml

Wider system implications

Limited, largely anecdotal evidence from the included studies prevented us developing theories on wider system implications. There were no reports of emergency department clinicians encouraged to adopt a more conservative approach, as a result of working alongside general practitioners, but some reports of general practitioners in management positions influencing system changes.(115,116) The potential reduction in learning opportunities for junior doctors was highlighted in two articles.(49,84) There was limited evidence that working in an emergency department setting led to increased job satisfaction for some United Kingdom general practitioners with a special interest in emergency care.(33,116) However, reduced satisfaction was also described because the job was outside the scope of usual general practice,(33,47) possibly contributing towards recruitment problems.(33,117)

Discussion

Principal findings

We developed theories using data from 96 articles to explain how contextual factors are linked to outcomes: about the streaming process itself; the role general practitioners may adopt in the emergency department setting; and the effects of these on the patient (experience and safety) and the organisation (risk of provider-induced demand and costs). There was little evidence that general practitioners in emergency departments directly or indirectly affected the care and throughput of the sickest patients. Distinct services may offer an attractive alternative to primary care and result in provider-induced demand. The literature describing economic impacts of general practitioners in emergency departments

For peer review only - http://bmjopen.bmj.com/site/about/guidelines.xhtml

comes from different countries, with different funding systems and spans over 20 years, limiting conclusions.

Strengths and limitations

Heterogeneous studies involving general practitioners working in or alongside emergency departments do not suit traditional systematic review methods. We have conducted the first realist review in this area, methods that are gaining prominence in healthcare research.(118,119) Specifically, we adopted the “rapid realist review” method, which includes a reference group of knowledge users and external experts, to provide policy makers with context specific evidence, quantification of existing knowledge and a summary of evidence gaps.(24) It identifies context, mechanisms and outcomes, including tensions in the evidence base, to suggest how a service is likely to work, for whom, in what circumstances and why.(21) The rapid realist review approach is appropriate in relation to the rapidly evolving NHS policy on emergency department use of general practitioners, (10–12) showing where such policies may be reinforced or refuted by the evidence available.(24) A weakness of our study was the time constraints of our project but the expert opinion via the reference group mitigated this, and enabled us to focus and direct our research.(24) Some studies did not describe the intervention in adequate depth to help facilitate the identification of key mechanisms. Also, the nature of different healthcare and funding systems limited international comparability.(21)

Comparison with other reviews

Before our review, the largest review to date by Ramlakhan et al. (2016) included 20 papers and described provider-induced demand, poor evidence for improved emergency

For peer review only - http://bmjopen.bmj.com/site/about/guidelines.xhtml

department throughput and minimal economic impact.(17) The Goncalves-Bradley et al. Cochrane review of four studies, published in 2018, highlighted inconsistent results and a lack of evidence on safety.(18) We also found evidence of provider-induced demand in distinct primary care units but less so in more fully integrated service models where patients lacked awareness that they had been directed to primary care services.(4,62,63) We found that patients with primary care problems may have reduced process times if treated by general practitioners adopting a traditional role but there was a lack of evidence for the improved care of seriously unwell patients in the department. There was also a lack of evidence on the impact on general practitioners' cognition processes and risk-taking behaviour when treating a different group of patients to that seen in usual general practice and the safety implications of this.

Policy implications

The global health priority recently given to Universal Health Coverage,(120) and the attention being given to the 40th anniversary of the Alma Ata declaration,(121) moves to centre stage the design of primary healthcare systems, particularly their capacity and capability to respond to urgent care needs. Internationally, emergency departments are exploring options on how to run more efficiently and safely. Our theories, informed by literature from 13 high-income countries, allow policy makers to make more considered judgements about their relevance to their own contexts for service provision. The United Kingdom has already commissioned further research in this area, funded by the National Institute for Health Research (HS&DR Projects: 15/145/04(32) and 15/145/06(122)), the former collecting primary data to further test and refine these theories.

For peer review only

For peer review only - http://bmjopen.bmj.com/site/about/guidelines.xhtml

Conclusion

The effectiveness of emergency department streaming to primary care services may be influenced by how staff interpret the streaming system and the roles general practitioners adopt. Caution is needed in embedding the policy until further evaluation is available. Service models that encourage the traditional general practitioner approach may have shorter process times; however, there is little evidence on the safety implications or whether this improves care for the sickest patients. Distinct primary care services offering increased patient choice may result in provider-induced demand. Economic evaluation requires further research.

Acknowledgements

Many thanks to our public contributors, Barbara Harrington and Julie Hepburn, and to Professor Damon Berridge for contributing to discussions. Also to Faris Hussain for assisting with citation tracking.

Contributors

All authors are co-applicants on the wider project and were involved in the conceptualisation of the study. AC and FD planned the synthesis approach with input from AE. The core review team AP, PA, BE, BH, JD and ACS then met via teleconference every 6 weeks to discuss findings and guide further searches. AC conducted the database searches. AC and FD extracted data extracts and were involved in the synthesis process, meeting weekly with AE and ME to discuss findings. AC prepared the first draft of the manuscript which was reviewed and critically appraised by all authors.

For peer review only

Funding

This work was supported by the National Institute for Health Research HS&DR Project 15/145/04

Competing interests

None declared

Data sharing statement

No further data available

For peer review only

References

1. What's going on with A&E waiting times? The King's Fund [Internet]. [cited 2018 Feb 5]. Available from: https://www.kingsfund.org.uk/projects/urgent-emergency-care/urgent-and-emergency-care-mythbusters
2. Keogh B. Transforming urgent and emergency services in England: Urgent and emergecy care review [Internet]. 2013. Available from: http://www.nhs.uk/NHSEngland/keogh-review/Pages/urgent-and-emergency-care-review.aspx
3. Emergency departments: More useful than the official data suggests [Internet]. The College of Emergency Medicine. 2014. Available from: http://www.kingstoned.org/uploads/2/4/0/2/24023085/ca_past_paper.pdf
4. Ward P, Huddy J, Hargreaves S, Touquet R, Hurley J, Fothergill J. Primary care in London: an evaluation of general practitioners working in an inner city accident and emergency department. J Accid Emerg Med. 1996;13(table 1):11–5.
5. Smith L, Narang Y, Pavon A, Edwardson K, Bowers S, Jones K, et al. To GP or not to GP: Evaluation of children triaged to see a GP in a tertiary paediatric emergency department. BMJ Qual Saf [Internet]. 2017;0:1–8. Available from: http://ovidsp.ovid.com/ovidweb.cgi?T=JS&PAGE=reference&D=emed18&NEWS=N&AN=612211602
6. Dale J, Reid F, Green J, Glucksman E. Primary care in the accident and emergency department: I. Prospective identification of patients. BMJ [Internet]. 1995 [cited 2017 Sep 13];311:423–6. Available from: https://www.ncbi.nlm.nih.gov/pmc/articles/PMC2550493/pdf/bmj00605-0027.pdf
7. Coleman P, Irons R, Nicholl J. Will alternative immediate care services reduce demands for non-urgent treatment at accident and emergency? Emerg Med J. 2001;18(6):482–7.
8. Nagree Y, Camarda V, Fatovich D, Cameron P, Dey I, Gosbell A, et al. Quantifying the proportion of general practice and low-acuity patients in the emergency department. 2013;198(June):612–5.
9. Thompson MIW, Lasserson D, Mccann L, Thompson M, Heneghan C. Suitability of emergency department attenders to be assessed in primary care : survey of general practitioner agreement in a random sample of triage records analysed in a service evaluation project. 2013;
10. Acute and emergency care: prescribing the remedy [Internet]. 2014. Available from: https://www.rcplondon.ac.uk/news/acute-and-emergency-care-prescribing-remedy
11. A&E departments to get more funding - GOV.UK [Internet]. [cited 2017 Nov 10]. Available from: https://www.gov.uk/government/news/ae-departments-to-get-more-funding
12. Next steps on the NHS five year forward view [Internet]. 2017. Available from: https://www.england.nhs.uk/wp-content/uploads/2017/03/NEXT-STEPS-ON-THE-NHS-FIVE-YEAR-FORWARD-VIEW.pdf
13. The principles and values of the NHS in England [Internet]. Department of Health; [cited 2017 Dec 18]. Available from: https://www.nhs.uk/NHSEngland/thenhs/about/Pages/nhscoreprinciples.aspx
14. Carson D, Clay H, Stern R, Primary Care Foundation. Primary care and emergency departments. 2010;(March). Available from: http://www.primarycarefoundation.co.uk/images/PrimaryCareFoundation/Downloading_Reports/Reports_and_Articles/Primary_Care_and_Emergency_Departments/Primary_Care_and_Emergency_Departments_RELEASE.pdf
15. Khangura JK, Flodgren G, Perera R, Rowe BH SS. Primary care professionals providing non-urgent care in hospital emergency departments. Cochrane Libr. 2012;53(11):1689–99.
16. Turner J, Coster J, Chambers D, Cantrell A, Phung V-H, Knowles E, et al. What evidence is there on the effectiveness of different models of delivering urgent care? A rapid review. Heal Serv Deliv Res [Internet]. 2015;3(43):1–134. Available from: http://eprints.lincoln.ac.uk/17833/2/WR3-13-05-12.pdf%5Cnhttp://www.journalslibrary.nihr.ac.uk/hsdr/volume-3/issue-43
17. Ramlakhan S, Mason S, O'Keeffe C, Ramtahal A, Ablard S, Burke D, et al. Primary care services located with EDs: a review of effectiveness. Emerg Med J [Internet]. 2016;33(7):495–503. Available from: http://www.ncbi.nlm.nih.gov/pubmed/27068868
18. Goncalves-Bradley D, Khangura JK, Flodgren G, Perera R, Rowe BH, Shepperd S. Primary care professionals providing non-urgent care in hospital emergency departments. Cochrane Database Syst Rev [Internet]. 2012;11(11):N.PAG-N.PAG. Available from: http://onlinelibrary.wiley.com/doi/10.1002/14651858.CD002097.pub3/abstract%5Cnhttp://search.eb

For peer review only - http://bmjopen.bmj.com/site/about/guidelines.xhtml

scohost.com/login.aspx?direct=true&db=cin20&AN=107992519&site=ehost-live
19. Baird B, Charles A, Honeyman M, Maguire D, Das P. Understanding pressures in general practice [Internet]. The King's Fund. 2016. Available from: https://www.kingsfund.org.uk/sites/files/kf/field/field_publication_file/Understanding-GP-pressures-Kings-Fund-May-2016.pdf
20. NICE guideline GPs within or on the same site as emergency departments. 2017;(July).
21. Pawson R, Tilley N. Realistic evaluation. SAGE Publications; 2008.
22. Lavis J, Davies H, Oxman A, Denis J-L, Golden-Biddle K, Ferlie E. Towards systematic reviews that inform health care management and policy-making. J Health Serv Res Policy [Internet]. 2005 [cited 2017 Aug 30];10:35–48. Available from: http://journals.sagepub.com/doi/pdf/10.1258/1355819054308549
23. Rycroft-Malone J, Burton CR, Williams L, Edwards S, Fisher D, Hall B, et al. Improving skills and care standards in the support workforce for older people: a realist synthesis of workforce development interventions [Internet]. NIHR HS&DR. 2016 [cited 2018 Feb 19]. p. 2050–4349. Available from: https://www.ncbi.nlm.nih.gov/books/NBK355884/pdf/Bookshelf_NBK355884.pdf
24. Saul J, Willis C, Bitz J, Best A. A time-responsive tool for informing policy making: rapid realist review. Implement Sci [Internet]. 2013 [cited 2017 Jun 7];8(103). Available from: http://download.springer.com/static/pdf/412/art%253A10.1186%252F1748-5908-8-103.pdf?originUrl=http%3A%2F%2Fimplementationscience.biomedcentral.com%2Farticle%2F10.1186%2F1748-5908-8-103&token2=exp=1496844827~acl=%2Fstatic%2Fpdf%2F412%2Fart%25253A10.1186%25
25. Wong G, Greenhalgh T, Westhorp G, Buckingham J, Pawson R. RAMESES publication standards: realist syntheses. BMC Med [Internet]. 2013 [cited 2017 Jun 7];11(21):1–14. Available from: http://www.wileyonlinelibrary.com/journal/jan
26. Dale J, Green J, Reid F, Glucksman E, Higgs R. Primary care in the accident and emergency department: II. Comparison of general practitioners and hospital doctors. BMJ [Internet]. 1995;311(7002):427–30. Available from: http://www.pubmedcentral.nih.gov/articlerender.fcgi?artid=2550494&tool=pmcentrez&rendertype=abstract
27. Dale J, Lang H, Roberts JA, Green J, Glucksman E. Cost effectiveness of treating primary care patients in accident and emergency: A comparison between general practitioners, senior officers, and registrars. Br Med J [Internet]. 1996;312(7042):1340. Available from: http://ezproxy.library.dal.ca/login?url=http://search.proquest.com/docview/203994036?accountid=10406%5Cnhttp://sfxhosted.exlibrisgroup.com/dal?url_ver=Z39.88-2004&rft_val_fmt=info:ofi/fmt:kev:mtx:journal&genre=article&sid=ProQ:ProQ%3Anursing&atitle=Cost+e
28. Salisbury C, Hollinghurst S, Montgomery A, Cooke M, Munro J, Sharp D, et al. The impact of co-located NHS walk-in centres on emergency departments. Emerg Med J. 2007;24(4):265–9.
29. Primary Care in Emergency Departments A guide to good practice. NHS Interim Mangement Support [Internet]. 2015 [cited 2017 Nov 1]; Available from: http://www.nhsimas.nhs.uk/fileadmin/Files/IST/Emergency_care_conference_2014/Primary_Care_in_A_E_Guidance_Feb_2015.pdf
30. Barach P, Johnson JK. Understanding the complexity of redesigning care around the clinical microsystem. Qual Saf Heal Care [Internet]. 2006 [cited 2018 Feb 5];15:10–6. Available from: http://qualitysafety.bmj.com/content/qhc/15/suppl_1/i10.full.pdf
31. Pawson R. Evidence-Based Policy. SAGE Publications Ltd; 2006.
32. Edwards A. Evaluating effectiveness, safety, patient experience and system implications of different models of using GPs in or alongside Emergency Departments. [Internet]. [cited 2018 Feb 16]. Available from: https://www.journalslibrary.nihr.ac.uk/programmes/hsdr/1514504/#/
33. Ablard S, O'Keeffe C, Ramlakhan S, Mason SM. Primary care services co-located with Emergency Departments across a UK region: early views on their development. Emerg Med J [Internet]. 2017;(Published Online First: [May 15, 2017].):1–4. Available from: http://emj.bmj.com/content/early/2017/05/09/emermed-2016-206539
34. Bickerton J. Streaming A&E patients to walk-in centre services. Emerg nurse. 2005;13(3):20–4.
35. van der Straten LM, van Stel HF, Spee FJM, Vreeburg ME, Schrijvers AJP, Sturms LM. Safety and efficiency of triaging low urgent self-referred patients to a general practitioner at an acute care post: an observational study. Emerg Med J [Internet]. 2012;29(11):877–81. Available from: http://emj.bmj.com/lookup/doi/10.1136/emermed-2011-200539

For peer review only - http://bmjopen.bmj.com/site/about/guidelines.xhtml

36. Chmiel C, Wang M, Sidler P, Eichler K, Rosemann T, Senn O. Implementation of a hospital-integrated general practice--a successful way to reduce the burden of inappropriate emergency-department use. Swiss Med Wkly [Internet]. 2016;146:w14284. Available from: http://ovidsp.ovid.com/ovidweb.cgi?T=JS&PAGE=reference&D=medl&NEWS=N&AN=26963904
37. van Gils-van Rooij ESJ, Meijboom BR, Broekman SM, Yzermans CJ, de Bakker DH. Is patient flow more efficient in Urgent Care Collaborations? [Internet]. European Journal of Emergency Medicine. aScientific Center for Transformation in Care and Welfare (Tranzo), Tilburg School of Social and Behavioral Sciences, Tilburg University, Tilburg bOut-of-hours General Practitioner service Oost-Brabant, 's-Hertogenbosch cThe Netherlands Institute for Heal; 2016. Available from: https://www.scopus.com/inward/record.uri?eid=2-s2.0-84973377027&doi=10.1097%2FMEJ.0000000000000412&partnerID=40&md5=e93f55d49d861dac3195891cb4223380
38. Dale J. Primary Care in Accident and Emergency Departments : the cost effectiveness and applicability of a new model of care. 1998;1–242.
39. Bolton P, Thompson L. The reasons for, and lessons learned from, the closure of the Canterbury GP After-Hours Service. Aust Health Rev [Internet]. 2001;24(3):66–73. Available from: http://www.ncbi.nlm.nih.gov/pubmed/11668928
40. Thijssen W a MH, Wijnen-van Houts M, Koetsenruijter J, Giesen P, Wensing M. The impact on emergency department utilization and patient flows after integrating with a general practitioner cooperative: an observational study. Emerg Med Int [Internet]. 2013;2013:364659. Available from: http://www.pubmedcentral.nih.gov/articlerender.fcgi?artid=3814098&tool=pmcentrez&rendertype=abstract
41. Van Veen M, Wolde F, Poley M, Ruige M, Meurs A, Hable C, et al. Referral of nonurgent children from the emergency department to general practice: compliance and cost savings. Eur J Emerg Med. 2012;19:14–9.
42. Murphy A, Bury G, Plunkett PK, Gibney D, Smith M, Mullan E, et al. Randomised controlled trial of general practitioner versus usual medical care in an urban accident and emergency department: process, outcome, and comparative cost. BMJ. 1996;312(May):1135–42.
43. Wilson H. Co-locating primary care facilities within emergency departments: Brilliant innovation or unwelcome intervention into clinical care? N Z Med J. 2005;118(1221):1–11.
44. Boeke AJP, Van Randwijck-Jacobze ME, De Lange-Klerk EMS, Grol SM, Kramer MHH, Van Der Horst HE. Effectiveness of GPs in accident and emergency departments. Br J Gen Pract. 2010;60(579):378–84.
45. Wang M, Wild S, Hilfiker G, Chmiel C, Sidler P, Eichler K, et al. Hospital-integrated general practice: A promising way to manage walk-in patients in emergency departments. J Eval Clin Pract. 2014;20(1):20–6.
46. Huibers L, Thijssen W, Koetsenruijter J, Giesen P, Grol R, Wensing M. GP cooperative and emergency department: An exploration of patient flows. J Eval Clin Pract. 2013;19(2).
47. Kool RB, Homberg DJ, Kamphuis HCM. Towards integration of general practitioner posts and accident and emergency departments: a case study of two integrated emergency posts in the Netherlands. BMC Health Serv Res. 2008;8:225.
48. Bosmans JE, Boeke a J, van Randwijck-Jacobze ME, Grol SM, Kramer MH, van der Horst HE, et al. Addition of a general practitioner to the accident and emergency department: a cost-effective innovation in emergency care. Emerg Med J [Internet]. 2012;29(3):192–6. Available from: http://www.ncbi.nlm.nih.gov/pubmed/21441265
49. Hess S, Sidler P, Chmiel C, Bogli K, Senn O. Satisfaction of health professionals after implementation of a primary care hospital emergency centre in Switzerland: A prospective before-after study [Internet]. Vol. 23, International emergency nursing. United Kingdom; 2015. p. 286–93. Available from: http://ovidsp.ovid.com/ovidweb.cgi?T=JS&PAGE=reference&D=emex&NEWS=N&AN=616509018
50. Gibney D, Murphy AW, Barton D, Byrne C, Smith M, Bury G, et al. Randomized controlled trial of general practitioner versus usual medical care in a suburban accident and emergency department using an informal triage system. Br J Gen Pract. 1999;49(438):43–4.
51. Gritz A, Sen A, Hiles S, Mackenzie G. More under-fives now seen in urgent care centre than a&e-should we shift our focus? [Internet]. Archives of Disease in Childhood. A. Gritz, Paediatrics, Northwick Park Hospital, Harrow, United Kingdom: BMJ Publishing Group; 2016. p. A132. (Annual Conference of the Royal College of Paediatrics and Child Health, RCPCH 2016. United Kingdom.; vol. 101). Available from: http://ovidsp.ovid.com/ovidweb.cgi?T=JS&PAGE=reference&D=emed18&NEWS=N&AN=612211417
52. Gnani S, Morton S, Ramzan F, Davison M, Ladbrooke T, Majeed A, et al. Healthcare use among

For peer review only - http://bmjopen.bmj.com/site/about/guidelines.xhtml

preschool children attending GP-led urgent care centres: a descriptive, observational study. BMJ Open [Internet]. 2016;6(6):e010672. Available from: http://ovidsp.ovid.com/ovidweb.cgi?T=JS&PAGE=reference&D=prem&NEWS=N&AN=27288373

53. Clancy E, Mayo A. Launching a social enterprise see-and-treat service. Emerg Nurse. 2009;17(3):22–4.
54. Proctor N, Woodward M, Partnership HL. A & E Avoidance Schemes across London A rapid review of good practice examples. 2016.
55. Harris T, McDonald K. How do clinicians with different training backgrounds manage walk-in patients in the ED setting? Emerg Med J [Internet]. 2014;31(12):975–9. Available from: http://emj.bmj.com/lookup/doi/10.1136/emermed-2013-202844
56. Arain M, Nicholl J, Campbell M. Patients' experience and satisfaction with GP led walk-in centres in the UK; a cross sectional study. BMC Health Serv Res [Internet]. 2013;13:142. Available from: http://www.pubmedcentral.nih.gov/articlerender.fcgi?artid=3637583&tool=pmcentrez&rendertype=abstract
57. Chalder M, Montgomery A, Hollinghurst S, Cooke M, Munro J, Lattimer V, et al. Comparing care at walk-in centres and at accident and emergency departments: an exploration of patient choice, preference and satisfaction. Emerg Med J [Internet]. 2007;24(4):260–4. Available from: http://www.scopus.com/inward/record.url?eid=2-s2.0-34247245165&partnerID=tZOtx3y1
58. Hunter C, Chew-Graham C, Langer S, Stenhoff A, Drinkwater J, Guthrie E, et al. A qualitative study of patient choices in using emergency health care for long-term conditions: The importance of candidacy and recursivity. Patient Educ Couns [Internet]. 2013;93(2):335–41. Available from: http://dx.doi.org/10.1016/j.pec.2013.06.001
59. Rajpar SF, Smith M a, Cooke MW. Study of choice between accident and emergency departments and general practice centres for out of hours primary care problems. J Accid Emerg Med. 2000;17:18–21.
60. Hutchison B, Østbye T, Barnsley J, Stewart M, Mathews M, Campbell MK, et al. Patient satisfaction and quality of care in walk-in clinics, family practices and emergency departments: The Ontario walk-in clinic study. C Downloads/82341895-E9B4-4AB2-A0EA-CC54D761B9BA/low acuity patient ED au 2013.ped.pdf. 2003;168(8):977–83.
61. Arain M, Baxter S, Nicholl J. Perceptions of healthcare professionals and managers regarding the effectiveness of GP-led walk-in centres in the UK. BMJ Open [Internet]. 2015;5(8):e008286. Available from: http://ovidsp.ovid.com/ovidweb.cgi?T=JS&PAGE=reference&D=medl&NEWS=N&AN=26297367
62. Van Uden C, Crebolder H. Does setting up out of hours primary care cooperatives outside a hospital reduce demand for emergency care? Emerg Med J. 2004;21(6):722–3.
63. Van Uden C, Winkens R, Wesseling G, Fiolet H, Van Schayck O, Crebolder H. The impact of a primary care physician cooperative on the caseload of an emergency department: The Maastricht integrated out-of-hours service. J Gen Intern Med. 2005;20(7):612–7.
64. Krakau I, Hassler E. Provision for clinic patients in the ED produces more nonemergency visits. Am J Emerg Med [Internet]. 1999 Jan [cited 2017 Jun 15];17(1):18–20. Available from: http://linkinghub.elsevier.com/retrieve/pii/S0735675799900062
65. Hsu RT, Lambert PC, Dixon-Woods M, Kurinczuk JJ. Effect of NHS walk-in centre on local primary healthcare services: before and after observational study. BMJ [Internet]. 2003;326(7388):530. Available from: http://www.ncbi.nlm.nih.gov/pubmed/12623913%5Cnhttp://www.pubmedcentral.nih.gov/articlerender.fcgi?artid=PMC150464
66. Anantharaman V. Impact of health care system interventions on emergency department utilization and overcrowding in Singapore. Int J Emerg Med [Internet]. 2008 Apr 14 [cited 2017 Sep 8];1(1):11–20. Available from: http://www.springerlink.com/index/10.1007/s12245-008-0004-8
67. Maheswaran R, Pearson T, Jiwa M. Repeat attenders at National Health Service walk-in centres - a descriptive study using routine data. Public Health [Internet]. 2009;123(7):506–10. Available from: http://dx.doi.org/10.1016/j.puhe.2009.06.008
68. O'Kelly F, Teljeur C, Carter I, Plunkett P. Impact of a GP cooperative on lower acuity emergency department attendances. Emerg Med J [Internet]. 2010;27(10):770–3. Available from: http://emj.bmj.com/cgi/doi/10.1136/emj.2009.072686
69. Kork A, Vakkuri J. Improving access and managing healthcare demand with walk-in clinic Convenient, but at what cost? Int J Public Sect Manag. 2016;29(2):148–63.
70. Greenfield G, Ignatowicz A, S G, Bucktowonsing M, Ladbrooke T, Millington H, et al. Staff perceptions on patient motives for attending GP-led urgent care centres in London: A qualitative study. BMJ Open [Internet]. 2016;6(1):e007683. Available from:

For peer review only - http://bmjopen.bmj.com/site/about/guidelines.xhtml

http://ovidsp.ovid.com/ovidweb.cgi?T=JS&PAGE=reference&D=medl&NEWS=N&AN=26769775
71. Tammes P, Morris RW, Brangan E, Checkland K, England H, Huntley A, et al. Exploring the relationship between general practice characteristics, and ttendance at walk-in centres, minor injuries units and EDs in England 2012/2013: A cross-sectional study. Emerg Med J [Internet]. 2016;33(10):702–8. Available from: http://emj.bmj.com/
72. Colliers A, Remmen R, Streffer M-L, Michiels B, Bartholomeeusen S, Monsieurs KG, et al. Implementation of a general practitioner cooperative adjacent to the emergency department of a hospital increases the caseload for the GPC but not for the emergency department. Acta Clin Belg [Internet]. 2017;72(1):49–54. Available from: http://www.tandfonline.com/loi/yacb20#.VwH3Xk1f1Qs
73. Freeman GK, Meakin RP, Lawrenson RA, Leydon GM, Craig G. Primary care units in A and E departments in North Thames in the 1990s: Initial experience and future implications. Br J Gen Pract. 1999;49(439):107–10.
74. Cowling T, Ramzan F, Ladbrooke T, Millington H, Majeed A, Gnani S. Referral outcomes of attendances at general practitioner-led urgent care centres in London, England: retrospective analysis of hospital administrative data. Emerg Med J. 2015;doi: 10.11.
75. van Uden CJT, Ament AJH a, Voss GBWE, Wesseling G, Winkens RAG, van Schayck OCP, et al. Out-of-hours primary care. Implications of organisation on costs. BMC Fam Pract [Internet]. 2006;7:29. Available from: http://www.pubmedcentral.nih.gov/articlerender.fcgi?artid=1464145&tool=pmcentrez&rendertype=abstract%5Cnhttp://dl2af5jf3e.search.serialssolutions.com/?url_ver=Z39.88-2004&rft_val_fmt=info:ofi/fmt:kev:mtx:journal&rfr_id=info:sid/Ovid:med4&rft.genre=article
76. Van Gils-Van Rooij ESJ, Yzermans CJ, Broekman SM, Meijboom BR, Welling GP, De Bakker DH. Out-of-Hours Care Collaboration between general practitioners and hospital emergency departments in the Netherlands. J Am Board Fam Med. 2015;28(6):807–15.
77. Van Veelen M, Van den Brand C, Reijnen R, Van der Linden M. Effects of a general practitioner cooperative co-located with an emergency department on patient throughput. World J Emerg Med [Internet]. 2016;7(4):270–3. Available from: http://ovidsp.ovid.com/ovidweb.cgi?T=JS&CSC=Y&NEWS=N&PAGE=fulltext&D=emed13&AN=2015817712%5Cnhttp://sfx.ucl.ac.uk/sfx_local?sid=OVID:embase&id=pmid:&id=doi:10.5847/wjem.j.1920-8642.2015.01.008&issn=1920-8642&isbn=&volume=6&issue=1&spage=44&pages=44-47&dat
78. Arain M, Campbell MJ, Nicholl JP. Impact of a GP-led walk-in centre on NHS emergency departments. Emerg Med J [Internet]. 2015;(April 2009):1–6. Available from: http://www.ncbi.nlm.nih.gov/pubmed/24406328
79. van Uden CJT, Winkens RAG, Wesseling GJ, Crebolder HFJM, van Schayck CP. Use of out of hours services: a comparison between two organisations. Emerg Med J [Internet]. 2003;20(2):184–7. Available from: http://www.ncbi.nlm.nih.gov/pubmed/12642541%5Cnhttp://www.pubmedcentral.nih.gov/articlerender.fcgi?artid=PMC1726038
80. Johnson SJ. Evidence for primary care services at A&E. BMJ [Internet]. 2015;350:h3352. Available from: http://ovidsp.ovid.com/ovidweb.cgi?T=JS&PAGE=reference&D=medl&NEWS=N&AN=26109566
81. Grant C, Nicholas R, Moore L, Salisbury C. An observational study comparing quality of care in walk-in centres with general practice and NHS Direct using standardised patients. BMJ [Internet]. 2002;324(7353):1556. Available from: http://www.ncbi.nlm.nih.gov/pubmed/12089094%5Cnhttp://www.pubmedcentral.nih.gov/articlerender.fcgi?artid=PMC116613
82. Schols A, Stevens F, Zeijen C, Dinant G, van Vugt C, Cals J. Access to diagnostic tests during GP out-of-hours care: A cross-sectional study of all GP out-of-hours services in the Netherlands. Eur J Gen Pract [Internet]. 2016;22(3):176–81. Available from: http://ovidsp.ovid.com/ovidweb.cgi?T=JS&PAGE=reference&D=prem&NEWS=N&AN=27362282
83. Posocco A, Scapinello MP, De Ronch I, Castrogiovanni F, Lollo G, Sergi G, et al. Role of out of hours primary care service in limiting inappropriate access to emergency department. Intern Emerg Med [Internet]. 2017; Available from: http://ovidsp.ovid.com/ovidweb.cgi?T=JS&PAGE=reference&D=medp&NEWS=N&AN=28547207
84. Morton S, Igantowicz A, Gnani S, Majeed A, G. G. Describing team development within a novel GP-led urgent care centre model: A qualitative study. BMJ Open [Internet]. 2016;6(6):e010224. Available from: http://ovidsp.ovid.com/ovidweb.cgi?T=JS&PAGE=reference&D=prem&NEWS=N&AN=27406636
85. Giesen P. Patients either contacting a general practice cooperative or accident and emergency

For peer review only - http://bmjopen.bmj.com/site/about/guidelines.xhtml

department out of hours: a comparison. Emerg Med J [Internet]. 2006;23(9):731–4. Available from: http://emj.bmj.com/cgi/doi/10.1136/emj.2005.032359
86. Chmiel C, Huber CA, Rosemann T, Zoller M, Eichler K, Sidler P, et al. Walk-ins seeking treatment at an emergency department or general practitioner out-of-hours service: a cross-sectional comparison. BMC Health Serv Res [Internet]. 2011;11(1):94. Available from: http://bmchealthservres.biomedcentral.com/articles/10.1186/1472-6963-11-94
87. Begum F, Khan H, Moss P. Solving the A & E crisis using GP lead triage and redirection Evaluation of GP-lead service to identify and re-direct patients from A & E to primary care services. 2016;83. Available from: https://www.myhealth.london.nhs.uk/sites/default/files/30. Solving the A%26E crisis using GP lead triage and redirection_0.pdf
88. Hansagi H, Carlsson B, Olsson M, Edhag O. Trial of a method of reducing inappropriate demands on a hospital emergency department. Public Health. 1987;101(2):99–105.
89. Rivara F, Wall H, Worley P, James K. Pediatric nurse triage: it's efficacy, safety and implications for care. J Pediatr Heal Care. 1991;5(6):291–8.
90. Derlet RW, Nishio D, Cole LM, Silva J. Triage of patients out of the emergency department: Three-year experience. Am J Emerg Med. 1992;10(3):195–9.
91. Derlet RW, Kinser D, Ray L, Hamilton B, McKenzie J. Prospective Identification and Triage of Nonemergency Patients Out of an Emergency Department: A 5-Year Study. Ann Emerg Med. 1995;25(2):215–23.
92. McGugan E. Primary care or A&E? A study of patients redirected from an accident and emergency department. Scott Med J. 2000;45(5):144–6.
93. Ellbrant J, Akeson J, Akeson PK, J. E, J. A. Pediatric emergency department management benefits from appropriate early redirection of nonurgent visits. Pediatr Emerg Care [Internet]. 2015;31(2):95–100. Available from: http://journals.lww.com/pec-online
94. Bentley JA, Thakore S, Morrison W, Wang W. Emergency Department redirection to primary care: a prospective evaluation of practice. Scott Med J [Internet]. 2017;62(1):2–10. Available from: http://www.uk.sagepub.com/journals/Journal202200
95. Shaw KN, Selbst SM, Gill FM. Indigent children who are denied care in the emergency department. Ann Emerg Med. 1990;19(1):59–62.
96. Gadomski AM, Perkis V, Horton L, Cross S, Stanton B. Diverting Managed Care Medicaid Patients From Pediatric Emergency Department Use. Pediatrics [Internet]. 1995;95(2):170–8. Available from: http://pediatrics.aappublications.org/content/95/2/170
97. Lowe RA, Bindman AB, Ulrich SK. Refusing care to emergency department patients: evaluation of published triage guidelines. Ann Emerg Med [Internet]. 1994;23(February):286–93. Available from: https://doi.org/10.1016/S0196-0644(94)70042-7
98. van Veen M, Steyerberg EW, Lettinga L, Ruige M, van Meurs AHJ, van der Lei J, et al. Safety of the Manchester Triage System to identify less urgent patients in paediatric emergence care: a prospective observational study. Arch Dis Child [Internet]. 2011;96(6):513–8. Available from: http://adc.bmj.com/cgi/doi/10.1136/adc.2010.199018
99. Birnbaum A, Gallagher EJ, Utkewicz M, Gennis P, Carter W. Failure to Validate a Predictive Model for Refusal of Care to Emergency-department Patients. Acad Emerg Med. 1994;1(3):213–7.
100. Dale J, Sandhu H, Lall R, Glucksman E. The patient, the doctor and the emergency department: A cross-sectional study of patient-centredness in 1990 and 2005. Patient Educ Couns. 2008;72(2):320–9.
101. Sandhu H, Dale J, Stallard N, Crouch R, Glucksman E. Emergency nurse practitioners and doctors consulting with patients in an emergency department: a comparison of communication skills and satisfaction. Emerg Med J [Internet]. 2009 [cited 2017 Nov 21];26:400–4. Available from: http://emj.bmj.com/content/emermed/26/6/400.full.pdf
102. Murphy AW, Plunkett PK, Bury G, Leonard C, al et. Effect of patients seeing a general practitioner in accident and emergency on their subsequent reattendance: Cohort study. Br Med J [Internet]. 2000;320(7239):903–4. Available from: http://ezproxy.library.dal.ca/login?url=http://search.proquest.com/docview/204000852?accountid=10406%5Cnhttp://sfxhosted.exlibrisgroup.com/dal?url_ver=Z39.88-2004&rft_val_fmt=info:ofi/fmt:kev:mtx:journal&genre=article&sid=ProQ:ProQ%3Anursing&atitle=Effect
103. Doran KM, Colucci AC, Hessler RA, Ngai CK, Williams ND, Wallach AB, et al. An Intervention Connecting Low-Acuity Emergency Department Patients With Primary Care: Effect on Future Primary Care Linkage. Ann Emerg Med [Internet]. 2013 Mar [cited 2017 Sep 8];61(3):312–321.e7. Available from:

For peer review only - http://bmjopen.bmj.com/site/about/guidelines.xhtml

http://linkinghub.elsevier.com/retrieve/pii/S0196064412016447
104. Lengu D, Kobbacy K, Sapountzis S, Kagioglou M. Application Of Simulation And Modelling In Managing Unplanned Healthcare Demand [Internet]. 2012. Available from: http://usir.salford.ac.uk/29360/1/D_Lengu_et_al_-_Application_of_simulation_and_modelling_in_managing_unplanned_healthcare_demand.pdf
105. Sharma A, Inder B. Impact of co-located general practitioner (GP) clinics and patient choice on duration of wait in the emergency department. Emerg Med J [Internet]. 2011;28(8):658–61. Available from: http://www.ncbi.nlm.nih.gov/pubmed/20668112
106. Schull MJ, Kiss A, Szalai J-P. The Effect of Low-Complexity Patients on Emergency Department Waiting Times. Annu Emerg Med [Internet]. 2007 [cited 2018 Feb 16];49:257–64. Available from: https://s3.amazonaws.com/academia.edu.documents/45757002/The_Effect_of_Low-Complexity_Patients_on20160518-23148-sk0q6f.pdf?AWSAccessKeyId=AKIAIWOWYYGZ2Y53UL3A&Expires=1518803030&Signature=ffSXDL5QnUO1pD9bopykhva%2F4kQ%3D&response-content-disposition=inlin
107. Vertesi L. Does the Canadian Emergency Department Triage and Acuity Scale identify non-urgent patients who can be triaged away from the emergency department? Can J Emerg Med. 2004;6(5):337–42.
108. Richardson DB, Mountain D. Myths versus facts in emergency department overcrowding and hospital access block. Med J Aust. 2009;190(7):369–74.
109. Allen P, Cheek C, Foster S, Ruigrok M, Wilson D, Shires L. Low acuity and general practice-type presentations to emergency departments: a rural perspective. Emerg Med Australas [Internet]. 2015;27(2):113–8. Available from: http://ovidsp.ovid.com/ovidweb.cgi?T=JS&PAGE=reference&D=medl&NEWS=N&AN=25720647
110. O'Cathain A, Knowles E, Turner J, Hirst E, Goodacre S, Nicholl J, et al. Variation in avoidable emergency admissions: multiple case studies of emergency and urgent care systems. J Health Serv Res Policy [Internet]. 2016 Jan;21(1):5–14. Available from: http://www.uk.sagepub.com/journals/Journal202186
111. Pope C, Chalder M, Moore L, Salisbury C. What do other local providers think of NHS walk-in centres? Results of a postal survey. Public Health. 2005;119(1):39–44.
112. Salisbury C, Chalder M, Scott TM, Pope C, Moore L. What is the role of walk-in centres in the NHS? BMJ [Internet]. 2002;324(7334):399–402. Available from: http://www.ncbi.nlm.nih.gov/pubmed/11850372%5Cnhttp://www.pubmedcentral.nih.gov/articlerender.fcgi?artid=PMC65536
113. Desborough J, Parker R, Forrest L. Development and implementation of a nurse-led walk-in centre: evidence lost in translation? J Health Serv Res Policy [Internet]. 2013;18(3):174–8. Available from: http://journals.sagepub.com/doi/10.1177/1355819613488574
114. Williams RM. The costs of visits to emergency departments. N Engl J Med [Internet]. 1996;334(10):642–6. Available from: http://www.ncbi.nlm.nih.gov/pubmed/8592529%5Cnhttp://www.nejm.org/doi/pdf/10.1056/NEJM199603073341007
115. Chew-Graham C, Rogers A, May C, Sheaf R, Ball E. A new role for the general practitioner? Reframing 'inappropriate attenders' to inappropriate services. Prim Heal Care Res Dev [Internet]. 2004;(5):60–7. Available from: http://dx.doi.org/10.1191/1463423604pc188oa
116. Dale J, Russell R, Harkness F, Wilkie V, Aiello M. Extended training to prepare GPs for future workforce needs: a qualitative investigation of a 1-year fellowship in urgent care. Br J Gen Pract [Internet]. 2017;67(662):e659–67. Available from: http://bjgp.org/lookup/doi/10.3399/bjgp17X691853
117. Van den Heede K, Quentin W, Dubois C, Devriese S, Van de Voorde C. The 2016 proposal for the reorganisation of urgent care provision in Belgium: A political struggle to co-locate primary care providers and emergency departments. Health Policy [Internet]. 2017;121(4):339–45. Available from: http://ovidsp.ovid.com/ovidweb.cgi?T=JS&PAGE=reference&D=prem&NEWS=N&AN=28222904
118. Greenhalgh T, Humphrey C, Hughes J, MacFarlane F, Butler C, Pawson R. How Do You Modernize a Health Service? A Realist Evaluation of Whole-Scale Transformation in London. Milbank Q. 2009;87(2):391–416.
119. Gillespie BM, Marshall A, Baker R, Cleary K, Davies H, Davies S. Implementation of safety checklists in surgery: a realist synthesis of evidence. Implement Sci [Internet]. 2015 Dec 28 [cited 2017 Sep 12];10(1):137. Available from: http://implementationscience.biomedcentral.com/articles/10.1186/s13012-015-0319-9
120. Report of the Expert Consultation on Primary Care Systems Profiles & Performance (Primasys). The

For peer review only

Alliance for Health Policy and Systems Research. 2015. p. 0–34.
121. Declaration of Alma-Ata [Internet]. 1978 [cited 2018 Feb 5]. Available from: http://www.who.int/publications/almaata_declaration_en.pdf
122. Benger J. General Practitioners and Emergency Departments (GPED): Efficient Models of Care [Internet]. [cited 2018 Feb 23]. Available from: https://www.journalslibrary.nihr.ac.uk/programmes/hsdr/1514506/#/

For peer review only

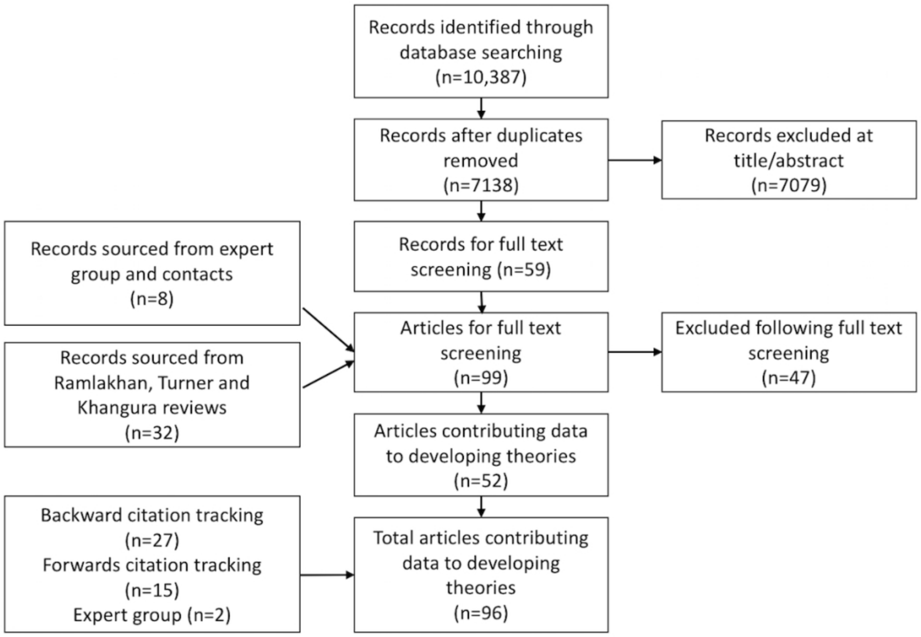

Figure 1: Search strategy and results

112x76mm (300 x 300 DPI)

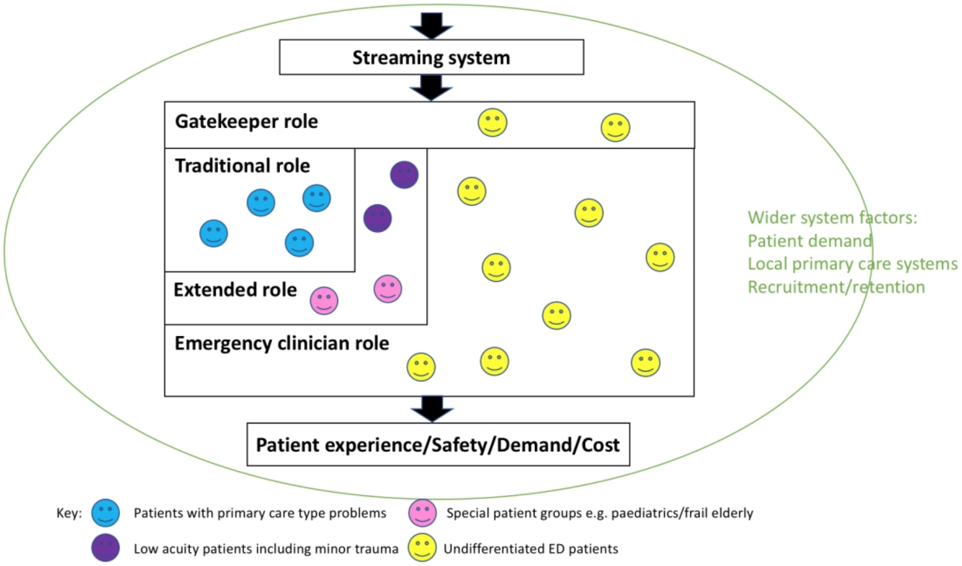

Figure 2: Potential roles general practitioners may adopt in the emergency department setting, influenced by the streaming system and impacting on outcomes

148x86mm (300 x 300 DPI)

Supplementary file 1: Search Strategy for Medline (adapted for other databases)

1. exp Primary Health Care/
2. primary care.mp. [mp=title, abstract, original title, name of substance word, subject heading word, keyword heading word, protocol supplementary concept word, rare disease supplementary concept word, unique identifier, synonyms]
3. exp Physicians, Family/
4. exp Physicians, Primary Care/
5. family physician*.mp. [mp=title, abstract, original title, name of substance word, subject heading word, keyword heading word, protocol supplementary concept word, rare disease supplementary concept word, unique identifier, synonyms]
6. exp Family Practice/
7. family practic*.mp. [mp=title, abstract, original title, name of substance word, subject heading word, keyword heading word, protocol supplementary concept word, rare disease supplementary concept word, unique identifier, synonyms]
8. GP.mp. [mp=title, abstract, original title, name of substance word, subject heading word, keyword heading word, protocol supplementary concept word, rare disease supplementary concept word, unique identifier, synonyms]
9. exp After-Hours Care/
10. (after-hours care or out of hours or out-of-hours or OOH).mp. [mp=title, abstract, original title, name of substance word, subject heading word, keyword heading word, protocol supplementary concept word, rare disease supplementary concept word, unique identifier, synonyms]
11. exp General Practitioners/
12. general practic*.mp. [mp=title, abstract, original title, name of substance word, subject heading word, keyword heading word, protocol supplementary concept word, rare disease supplementary concept word, unique identifier, synonyms]
13. 1 or 2 or 3 or 4 or 5 or 6 or 7 or 8 or 9 or 10 or 11 or 12
14. exp Emergency Medical Services/
15. exp Emergency Service, Hospital/
16. emergency department*.mp. [mp=title, abstract, original title, name of substance word, subject heading word, keyword heading word, protocol supplementary concept word, rare disease supplementary concept word, unique identifier, synonyms]
17. (accident and emergenc*).mp. [mp=title, abstract, original title, name of substance word, subject heading word, keyword heading word, protocol supplementary concept word, rare disease supplementary concept word, unique identifier, synonyms]
18. casualt*.mp. [mp=title, abstract, original title, name of substance word, subject heading word, keyword heading word, protocol supplementary concept word, rare disease supplementary concept word, unique identifier, synonyms]

19. emergency room.mp. [mp=title, abstract, original title, name of substance word, subject heading word, keyword heading word, protocol supplementary concept word, rare disease supplementary concept word, unique identifier, synonyms]

20. A&E.mp. [mp=title, abstract, original title, name of substance word, subject heading word, keyword heading word, protocol supplementary concept word, rare disease supplementary concept word, unique identifier, synonyms]

21. urgent care centre*.mp. [mp=title, abstract, original title, name of substance word, subject heading word, keyword heading word, protocol supplementary concept word, rare disease supplementary concept word, unique identifier, synonyms]

22. (walkin or walk in or walk-in).mp. [mp=title, abstract, original title, name of substance word, subject heading word, keyword heading word, protocol supplementary concept word, rare disease supplementary concept word, unique identifier, synonyms]

23. 14 or 15 or 16 or 17 or 18 or 19 or 20 or 21 or 22

24. 13 and 23

25. limit 24 to yr="2015 -Current"

review only

Supplementary file 2: Articles that contributed data to theory development (listed chronologically by Country)

Lead author	Country	Title	Study Design
Ablard 2017	UK	Primary care services co-located with Emergency Departments across a UK region: early views on their development	Survey and semi-structured interviews
Bentley 2017	UK	Emergency Department redirection to primary care: a prospective evaluation of practice	Prospective evaluation of the subsequent management and outcome of redirected non-urgent patients from a Scottish ED over 2 months
Dale 2017	UK	Extended training to prepare GPs for future workforce needs: a qualitative investigation of a one-year fellowship in urgent care	Qualitative investigation of a one-year fellowship in urgent care
Tammes 2016	UK	Exploring the relationship between general practice characteristics, and attendance at walk-in centres, minor injuries units and EDs in England 2012/2013: a cross-sectional study	Cross-sectional observational large data analysed using multivariable regression models
Proctor 2016	UK	A&E Avoidance schemes across London: A rapid review of good practice examples	NHS report - 2 case studies involving redirection of non-urgent patients from the ED
Smith 2016	UK	To GP or not TO GP: Evaluation of children triaged to see a GP in a tertiary paediatric emergency department	Retrospective cohort study of children classified as 'GP appropriate' seen by a GP between 14:00 and 22:00 and seen by ED staff outside these hours
Gnani 2016	UK	Healthcare use among preschool children attending GP-led urgent care centres: a descriptive observational study	Retrospective observational study using routinely collected data
O'Cathain 2016	UK	Variation in avoidable emergency admissions: multiple case studies of emergency and urgent care systems	Ethnographic residual analysis. Interviews with members of emergency care teams at 6 case study sites

For peer review only

Begum 2016	UK	Solving the A&E crisis using GP lead triage and redirection	Poster - Patient questionnaire of 150 patients over 5 weeks redirected back to the GP for treatment with an appointment made on the same day
Gritz 2016	UK	More under fives now seen in urgent care centre than A&E should we shift our focus	Observational - retrospective analysis of routine operational data for attendances
Greenfield 2016	UK	Staff perceptions on patient motives for attending GP-led urgent care centres in London: a qualitative study	Semi-structured interviews
Cowling 2015	UK	Referral outcomes of attendances at general practitioner-led urgent care centres in London, England: retrospective analysis of hospital administrative data	Retrospective analysis of administrative data recorded at a London urgent care centre of 243042 attendances from October 2009 to December 2012
Morton 2016	UK	Describing team development within a novel GP-led urgent care centre model: a qualitative study	Staff semi-structured interviews at 2 GP-led urgent care centres in 2 London academic teaching hospitals
Arain 2015a	UK	Perceptions of healthcare professionals and managers regarding the effectiveness of GP-led walk-in centres in the UK	Qualitative using a phenomenological approach using semi structured interviews
Arain 2015b	UK	Impact of a GP-led walk-in centre on NHS emergency departments	Patient survey over a 3-week period and analysis of attendances at the local children's hospital and minor injuries unit a year before and after the WIC opened
Johnson 2015	UK	Evidence of primary care services at A&E	Letter (opinion piece) Provider of 4 UCC in London supporting co-located GPs services with emergency departments

For peer review only

NHS ECIST 2015	UK	Primary care in emergency departments: a guide to good practice	NHS policy document - Overview of factors to be considered when planning how best to use primary care clinicians in emergency departments, monitoring and refining the service
Harris 2014	UK	How do clinicians with different training backgrounds manage walk-in patients in the ED setting?	Retrospective case note review of a random sample of 384 patients that self-presented to the ED and were initially assessed by GPs or ED staff
Thompson 2013	UK	Suitability of emergency department attenders to be assessed in primary care: survey of general practitioner agreement in a random sample of triage records analysed in a service evaluation project	Four GPs independently used data extracted from 765 clinical notes to rate the appropriateness for management in primary care
Arain 2013	UK	Patients' experience and satisfaction with GP led walk-in centres in the UK; a cross sectional study.	Patient survey over 3 weeks in 2 GP-led WICs
Hunter 2013	UK	A qualitative study of patient choices in using emergency health care for long-term conditions: The importance of candidacy and recursivity.	Questionnaire and semi-structured interviews
Lengu 2012	UK	Application of simulation and modelling in managing unplanned healthcare demand	Conference paper - Simulation and modelling to assess the impact of primary care clinicians deflecting patients with non-urgent needs away from A&E
Carson 2010	UK	Primary care and emergency departments	Report based on results of a literature review, web-based survey and ED visits
Clancy 2009	UK	Launching a social enterprise see-and-treat service	Report outlining the service, number of patients seen and referred on in a 4-month period
Maheswaren 2009	UK	Repeat attenders at national health service walk in centres	Descriptive study using routine data from 4 walk-in centres in England

Sandhu 2009	UK	Emergency nurse practitioners and doctors consulting with patients in an emergency department: a comparison of communication skills and satisfaction	Observation study with a stratified sample of 296 video-taped consultations
Dale 2008	UK	The patient, the doctor and the emergency department: A cross-sectional study of patient-centeredness in 1990 and 2005	Observational study with a stratified sample of 430 video-taped consultations with data collection in May–July 1990 and May–July 2005.
Salisbury 2007	UK	The impact of co-located NHS walk-in centres on emergency departments	Controlled before and after study
Chalder 2007	UK	Comparing care at walk-in centres and at accident and emergency departments: an exploration of patient choice, preference and satisfaction	A controlled, mixed-method study comparing 8 EDs with co-located WICs with the same number of ''traditional'' EDs.
Pope 2005	UK	What do other local providers think of NHS walk-in centres? Results of a postal survey	Postal survey
Bickerton 2005	UK	Streaming A&E patients to walk-in centre services	Analysis of all patients attending a London hospital over 24 hours for suitability for WIC treatment
Chew-Graham 2004	UK	A new role for the general practitioners? Reframing inappropriate attenders to inappropriate services	Qualitative semi-structured staff interviews
Hsu 2003	UK	Effect of NHS walk-in centre on local primary healthcare services	Before and after observational study of consultation rate in 12 general practices after the implementation of a walk-in centre
Salisbury 2002	UK	What is the role of walk-in centres in the NHS?	Analysis of routinely collected data, questionnaire completed by managers followed by semi-structured interviews and site visits
Grant 2002	UK	An observational study comparing quality of care in walk-in centres with general practice and NHS Direct using standardised patients	Observational study involving assessment of clinicians by standardised patients at 20 walk in centres, 20 general practices ad 11 NHS direct sites

For peer review only

Coleman 2001	UK	Will alternative immediate care services reduce demands for non-urgent treatment at accident and emergency?	Questionnaire survey and notes review of non-urgent patients to assess the suitability of management by an alternative care service
McGugan 2000	UK	Primary care or A&E?	Prospective study over 2 months of a redirection policy
Rajpar 2000	UK	Study of choice between accident and emergency departments and general practice centres for out of hours primary care problems	Interview of patients attending A&E and GP out-of-hours
Freeman 1999	UK	Primary care units in A and E departments in North Thames in the 1990s: Initial experience and future implications	Postal questionnaire to ED staff and local GPs with follow up staff interviews
Dale 1998	UK	Primary care in accident and emergency departments: the cost effectiveness and applicability of a new model of care	PhD thesis – Includes data for included papers and additional analysis of 163 video-taped consultations
Ward 1996	UK	Primary care in London: an evaluation of general practitioners working in an inner-city accident and emergency department	Prospective survey over 6 weeks
Dale 1996	UK	Cost effectiveness of treating primary care patients in accident and emergency: a comparison between GPs, senior house officers and registrars	Prospective intervention study which was retrospectively costed
Dale 1995a	UK	Primary care in the accident and emergency department I: Prospective identification of patients	1 year prospective study at a London ED to compare patient characteristics and consultation activities for attenders assessed by nurse triage to have 'primary care' or 'accident and emergency' type problems
Dale 1995b	UK	Primary care in the accident and emergency department: II. Comparison of general practitioners and hospital doctors	1 year prospective study at a London ED to compare patient characteristics and consultation activities for attenders assessed by nurse triage to have 'primary care' or 'accident and emergency' type problems

For peer review only

O'Kelly 2010	Ireland	Impact of a GP cooperative on lower acuity emergency department attendances	A retrospective review of all attendances at the 'Dubdoc' service was compared with attendances at the ED for triage categories 4 and 5 of the same hospital over a 9-year period
Murphy 2000	Ireland	Effect of patients seeing a general practitioner in accident and emergency on their subsequent attendance: cohort study	Analysis of reattendance of non-urgent patients that had been allocated to general practitioners or usual accident and emergency staff depending on time of registration
Gibney 1999	Ireland	Randomized controlled trial of general practitioner versus usual medical care in a suburban accident and emergency department using an informal triage system	Patients 'randomised' at time of registration to either GP or ED care. Case note review
Murphy 1996	Ireland	Randomised controlled trial of general practitioner versus usual medical care in an urban accident and emergency department: process, outcome and comparative cost	Randomised controlled trial of care provided by general practitioners to non-emergency patients in an accident and emergency department differs significantly from care by usual emergency staff in terms of process, outcome ad cost
van Veelen 2016	Netherlands	Effects of a general practitioner cooperative co-located with an emergency department on patient throughput	Pre-post comparison before and after implementation of a GP cooperative at an ED
Schols 2016	Netherlands	Access to diagnostic tests during GP out-of -hours care: A cross sectional study of all GP out-hours services in the Netherlands	Cross-sectional survey of all 117 GP out of hours services in the Netherlands
Van-Gils-van Rooij 2016	Netherlands	Is patient flow more efficient in urgent care collaborations?	Observational study, compared usual care with UCCs (single point of access for ED and GP OOH)

For peer review only

van Gils-van Rooij 2015	Netherlands	Out-of-Hours Care Collaboration between General Practitioners and Hospital Emergency Departments in the Netherlands	Observational study - comparing attendance and patient characteristics between EDs with standard care and EDs with co-located primary care and single joint triage
Thijssen 2013	Netherlands	The impact on emergency department utilization and patient flows after integrating with a general practitioner cooperative: an observational study	Observational study - routinely collected data over 6 years
Huibers 2013	Netherlands	GP cooperative and emergency department: an exploration of patient flows	Retrospective record review of patients who had visited GPC or ED
Van der Straten 2012	Netherlands	Safety and efficiency of triaging low urgent self-referred patients to a general practitioner at an acute care post: an observational study	Prospective observational study
Bosmans 2012	Netherlands	Addition of a general practitioner to the accident and emergency department: a cost-effective innovation in emergency care	Observational study before and after implementation of new service
Van Veen 2012	Netherlands	Van Veen referral of non-urgent children from the emergency department to general practice: compliance and cost savings	Prospective observational before after study
Van Veen 2011	Netherlands	Safety of the Manchester Triage System to identify less urgent patients in paediatric emergency care: a prospective observational study	Analysis of the hospitalisation rate of self referred children triaged as non-urgent
Boeke 2010	Netherlands	Effectiveness of GPs in accident and emergency departments	Observational study before and after implementation of new service
Kool 2008	Netherlands	Towards integration of general practitioner posts and accident and emergency departments: a case study of two integrated emergency posts in the Netherlands	Observational study comparing contacts, patient satisfaction and staff satisfaction pre-and post set up of a 2 co-located GP OOHs and 2 control sites

For peer review only

Giesen 2006	Netherlands	Patients either contacting a general practice cooperative or accident and emergency department out of hours: a comparison	Retrospective record review
Van Uden 2006	Netherlands	Out-of-hours primary care. Implications of organisation on costs	Annual reports of 2 GP co-operatives (1 co-located, 1 separate) analysed together with ED costs
Van Uden 2005	Netherlands	The Impact of a Primary Care Physician Cooperative on the Caseload of an Emergency Department: The Maastricht Integrated Out-of-Hours Service	Observational study, patient characteristics collected for 3 weeks in Jan/Fen 1998 and March 2001 (co-operative set up in 2000)
Van Uden 2004	Netherlands	Does setting up out of hours primary care cooperatives outside a hospital reduce demand for emergency care?	Before and after observational study
Van Uden 2003	Netherlands	Use of out of hours services: a comparison between two organisations	Observational study of patient contacts at 2 different OOH centres and their associated EDs (1 co-located, 1 not)
Colliers 2017	Belgium	Implementation of a general practitioner cooperative adjacent to the emergency department of a hospital increases the caseload for the GPC but not for the emergency department	Quasi-experimental study analysing the implementation of 2 out of hours general practitioner co-operatives one adjacent to the ED, the other not and 2 control sites
Van den Heede 2016	Belgium	The 2016 proposal for the reorganisation of urgent care provision in Belgium: A political struggle to co-locate primary care providers and emergency departments	Outline of the 2016 political proposal for the reorganisation of urgent care provision toned down due to GP opposition
Ellbrant 2015	Sweden	Paediatric emergency department management benefits from appropriate early redirection of non-urgent visits	Prospective observational study using ED records and case notes
Krakau 1999	Sweden	Provision for clinic patients in the ED produces more nonemergency visits	Before and after observational study

For peer review only

Hansagi 1987	Sweden	Trial of a method of reducing inappropriate demands on a hospital emergency department.	Prospective observational study of 454 patients classified as non-urgent by the ED and redirected to alternative care over a 3-month period
Chmiel 2016	Switzerland	Implementation of a hospital-integrated general practice – a successful way to reduce the burden of inappropriate emergency-department use	Longitudinal observational study
Hess 2015	Switzerland	Satisfaction of health professionals after implementation of a primary care hospital emergency centre in Switzerland: A prospective before-after study	Questionnaire study of job satisfaction before and after a new emergency care model was implemented in Switzerland
Wang 2014	Switzerland	Hospital integrated general practice: a promising way to manage walk in patients	Pre and post comparison study before and after implementation of a new hospital-integrated general practice model
Chmiel 2011	Switzerland	Walk-ins seeking treatment at an emergency department or general practitioner out-of-hours service: a cross-sectional comparison	Analysis of routinely collected data of 2974 patient encounters attending a GPC or ED
Posocco 2017	Italy	Role of out of hours primary care service in limiting inappropriate access to emergency department	Retrospective analysis of 408 ED referrals from a local OOH service
Kork 2016	Finland	Improving access and managing healthcare demand with walk in clinic: convenient but at what cost?	Observational study over 48 months of the characteristics of 107 frequent attenders at a WIC from electronic patient records
Allen 2015	Australia	Low acuity and general practice type presentations to emergency departments: A rural perspective	Analysis of GP type presentations to 2 rural EDs over a 4-month period
Desborough 2013	Australia	Development and implementation of a nurse-led walk-in centre: evidence lost in translation?	Evaluation of the first 12 months of operation of the first Australian public nurse-led primary care walk-in centre compared to the English NHS model.

Nagree 2013	Australia	Quantifying the proportion of general practice and low-acuity patients in the emergency department	Four methods for calculating general practice-type patients were compared for 3 tertiary EDs in Perth, Australia in 2009-2011
Sharma 2011	Australia	Impact of co-located general practitioner (GP) clinics and patient choice on duration of wait in the emergency department	Mathematical modelling of wait times using routine ED data
Richardson 2009	Australia	Myths versus facts in emergency department overcrowding and hospital access block.	Report referencing previous work
Bolton 2001	Australia	The reasons for, and lessons learned from, the closure of the Canterbury GP After-Hours Service.	Report describing why a 12-month trial of GP staffed after hours service with an ED was not continued because the opportunity cost was greater than existing alternative services
Doran 2013	USA	An intervention connecting low acuity emergency department patients with primary care: Effect on future primary care linkage	Analysis of primary care follow up of patients presenting to ED assessed to have non-urgent problem and referred to an onsite primary care clinic
Williams 1996	USA	The costs of visits to emergency departments.	Analysis of emergency department charges and costs based on data from 6 community hospitals
Gadomski 1995	USA	Diverting managed care Medicaid patients from pediatric emergency department use.	6-month follow up of Medicaid children with non-emergent conditions not authorised to be seen in the Pediatric Emergency Department by their primary care provider
Derlet 1995	USA	Prospective identification and triage of nonemergency patients out of an emergency department - 5 year study	5 year study to analyse the outcome of adult patients refused care in the ED
Derlet 1992	USA	Triage of patients out of the emergency department: three year experience.	3 year study to analyse the outcome of adult patients refused care in the ED

For peer review only

Birnbaum 1994	USA	Failure to validate a predictive model for refusal of care to emergency-department patients.	Analysis of the outcome of 534 patients that met the pre-established criteria for refusal of care
Lowe 1994	USA	Refusing care to emergency department patients: evaluation of published triage guidelines.	Case note review of 106 patients who would have been refused care according to triage guidelines
Shaw 1990	USA	Indigent children who are denied care in the emergency department.	Six-month prospective study of 588 children denied care in the emergency department
Rivara 1986	USA	Pediatric nurse triage: its efficacy, safety and implications for care.	Evaluation of emergency room triage of 748 children over a 6-week period at a large urban children's hospital that routinely referred outside of the institution for care
Schull 2007	Canada	The Effect of Low-Complexity Patients on Emergency Department Waiting Times	Analysis of 4.1 million patient visits over a 1 year period (2002-3) and 110 EDs of the effect of low-complexity patients on time of physician contact of high complexity patients
Vertesi 2004	Canada	Does the Canadian Emergency Department Triage and Acuity Scale identify non-urgent patients who can be triaged away from the emergency department?	Retrospective database audit in an urban referral hospital ED.
Hutchison 2003	Canada	Patient satisfaction and quality of care in walk-in clinics, family practices and emergency departments: the Ontario Walk-In Clinic Study.	Prospective cohort study of the quality of care of 8 common acute conditions and patient satisfaction
Anantharaman 2008	Singapore	Impact of health care system interventions on emergency department utilisation and overcrowding in Singapore	Retrospective analysis of attendances at six main public EDs over 32 years
Wilson 2005	New Zealand	Co-locating primary care facilities within emergency departments: brilliant innovation or unwelcome intervention into clinical care?	Report reviewing a proposal to co-locate a primary care facility within the local emergency department

For peer review only

For peer review only

For peer review only - http://bmjopen.bmj.com/site/about/guidelines.xhtml

Research checklist: RAMESES publication standards for realist syntheses(25)

List of items to be included when reporting a realist synthesis

TITLE			Reported on page
1		In the title, identify the document as a realist synthesis or review -	1
ABSTRACT			
2		While acknowledging publication requirements and house style, abstracts should ideally contain brief details of: the study's background, review question or objectives; search strategy; methods of selection, appraisal, analysis and synthesis of sources; main results; and implications for practice.	1-2
INTRODUCTION			
3	Rationale for review	Explain why the review is needed and what it is likely to contribute to existing understanding of the topic area.	3-4
4	Objectives and focus of review	State the objective(s) of the review and/or the review question(s). Define and provide a rationale for the focus of the review.	3-4
METHODS			
5	Changes in the review process	Any changes made to the review process that was initially planned should be briefly described and justified.	n/a
6	Rationale for using realist synthesis	Explain why realist synthesis was considered the most appropriate method to use.	5-6

For peer review only

TITLE			Reported on page
7	Scoping the literature	Describe and justify the initial process of exploratory scoping of the literature.	7
8	Searching processes	While considering specific requirements of the journal or other publication outlet, state and provide a rationale for how the iterative searching was done. Provide details on all the sources accessed for information in the review. Where searching in electronic databases has taken place, the details should include, for example, name of database, search terms, dates of coverage and date last searched. If individuals familiar with the relevant literature and/or topic area were contacted, indicate how they were identified and selected.	7
9	Selection and appraisal of documents	Explain how judgements were made about including and excluding data from documents, and justify these.	8
10	Data extraction	Describe and explain which data or information were extracted from the included documents and justify this selection.	8
11	Analysis and synthesis processes	Describe the analysis and synthesis processes in detail. This section should include information on the constructs analyzed and describe the analytic process.	8
RESULTS			
12	Document flow diagram	Provide details on the number of documents assessed for eligibility and included in the review with reasons for exclusion at each stage as well as an indication of their	Figure 1

For peer review only

TITLE			Reported on page
		source of origin (for example, from searching databases, reference lists and so on). You may consider using the example templates (which are likely to need modification to suit the data) that are provided.	
13	Document characteristics	Provide information on the characteristics of the documents included in the review.	9<br>And supplementary file 2
14	Main findings	Present the key findings with a specific focus on theory building and testing.	9-20
DISCUSSION			
15	Summary of findings	Summarize the main findings, taking into account the review's objective(s), research question(s), focus and intended audience(s).	20
16	Strengths, limitations and future research directions	Discuss both the strengths of the review and its limitations. These should include (but need not be restricted to) (a) consideration of all the steps in the review process and (b) comment on the overall strength of evidence supporting the explanatory insights which emerged.<br>The limitations identified may point to areas where further work is needed.	21
17	Comparison with existing literature	Where applicable, compare and contrast the review's findings with the existing literature (for example, other reviews) on the same topic.	21-22
18	Conclusion and	List the main implications of the findings and place these in the context of other	23

For peer review only

**TITLE**			**Reported on page**
	recommendations	relevant literature. If appropriate, offer recommendations for policy and practice.	
19	Funding	Provide details of funding source (if any) for the review, the role played by the funder (if any) and any conflicts of interests of the reviewers.	24

For peer review only

BMJ Open

The impact of general practitioners working in or alongside emergency departments: a rapid realist review

Journal:	*BMJ Open*
Manuscript ID	bmjopen-2018-024501.R1
Article Type:	Research
Date Submitted by the Author:	14-Dec-2018
Complete List of Authors:	Cooper, Alison; Cardiff University, Division of Population Medicine<br>Davies, Freya; Cardiff University, Division of Population Medicine<br>Edwards, Michelle; Cardiff University, Division of Population Medicine<br>Anderson, Pippa; Swansea University, Centre for Health Economics<br>Carson-Stevens, Andrew; Cardiff University, Division of Population Medicine<br>Cooke, Matthew; University of Warwick Warwick Medical School<br>Donaldson, Liam; London School of Hygiene and Tropical Medicine<br>Dale, Jeremy; University of Warwick, Warwick Medical School<br>Evans, Bridie; Swansea University, Medicine<br>Hibbert, Peter; Macquarie University Faculty of Medicine and Health Sciences, Faculty of Medicine and Health Sciences; University of South Australia Division of Health Sciences,<br>Hughes, Thomas; John Radcliffe Hospital, Emergency Department<br>Porter, Alison; University of Swansea, College of Medicine<br>Rainer, Tim; Cardiff University, Division of Population Medicine<br>Siriwardena, Aloysius; University of Lincoln, School of Health and Social Care<br>Snooks, Helen; Swansea University<br>Edwards, Adrian; Cardiff University, Division of Population Medicine
**Primary Subject Heading**:	Emergency medicine
Secondary Subject Heading:	General practice / Family practice, Health services research
Keywords:	Emergency Service, Hospital, Primary Health Care, General practitioners, Health Services Research

SCHOLARONE™
Manuscripts

The impact of general practitioners working in or alongside emergency departments: a rapid realist review

Cooper A, Davies F, Edwards M, Anderson P, Carson-Stevens A, Cooke M, Donaldson L, Dale J, Evans B, Hibbert P, Hughes T, Porter A, Rainer T, Siriwardena A, Snooks H, Edwards A

Corresponding author: Dr Alison Cooper, Division of Population Medicine, Cardiff University, 5th Floor Neuadd Meirionnydd, Heath Park, Cardiff CF14 4YS
CooperA8@cardiff.ac.uk 02920 870746

Dr Alison Cooper, Cardiff University, Cardiff, UK
Dr Freya Davies, Cardiff University, Cardiff, UK
Dr Michelle Edwards, Cardiff University, Cardiff, UK
A/Prof Pippa Anderson, Swansea University, Swansea, UK
Dr Andrew Carson-Stevens, Cardiff University, Cardiff, UK
Prof Matthew Cooke, Warwick University, Coventry, UK
Prof Sir Liam Donaldson, London School of Hygiene and Tropical Medicine. London, UK
Prof Jeremy Dale, Warwick University, Coventry, UK
Dr Bridie Evans, Swansea University, Swansea, UK
Mr Peter Hibbert, Macquarie University, Sydney, Australia
Dr Thomas Hughes, John Radcliffe Hospital, Oxford, UK
A/Prof Alison Porter, Swansea University, Swansea, UK
Prof Tim Rainer, Cardiff University, Cardiff, UK
Prof Aloysius Siriwardena, Lincoln University, UK
Prof Helen Snooks, Swansea University, Swansea, UK
Prof Adrian Edwards, Cardiff University, Cardiff, UK

Keywords: Emergency Service, Hospital; Primary Health Care; General practitioners; Health Services Research

Word count: 4256

For peer review only

Abstract

Objectives

Worldwide, emergency healthcare systems are under intense pressure from ever-increasing demand and evidence is urgently needed to understand how this can be safely managed. An estimated 10-43% of emergency department patients could be treated by primary care services. In England, this has led to a policy proposal and £100million of funding ($130million USD), for emergency departments to stream appropriate patients to a co-located primary care facility so they are “free to care for the sickest patients”. However, the research evidence to support this initiative is weak.

Design

Rapid realist literature review.

Setting

Emergency departments.

Inclusion criteria

Articles describing general practitioners working in or alongside emergency departments

Aim

To develop context-specific theories that explain how and why general practitioners working in or alongside emergency departments affect: patient flow; patient experience; patient safety; and the wider healthcare system.

Results

Ninety-six articles contributed data to theory development sourced from earlier systematic reviews, updated database searches (Medline, Embase, CINAHL, Cochrane DSR & CRCT, DARE, HTA Database, BSC, PsycINFO and SCOPUS), and citation tracking. We developed theories to explain: how staff interpret the streaming system; different roles general

For peer review only

practitioners adopt in the emergency department setting (traditional, extended, gatekeeper or emergency clinician); and how these factors influence patient (experience and safety) and organisational (demand and cost-effectiveness) outcomes.

Conclusions

Multiple factors influence the effectiveness of emergency department streaming to general practitioners; caution is needed in embedding the policy until further research and evaluation are available. Service models that encourage the traditional general practitioner approach may have shorter process times for non-urgent patients; however, there is little evidence that this frees up emergency department staff to care for the sickest patients. Distinct primary care services offering increased patient choice may result in provider-induced demand. Economic evaluation and safety requires further research.

Trial registration

Prospero ID=CRD42017069741

298/300

Strengths and limitations of this study

- A realist approach to evidence synthesis leads to theory development that explains how and why context links to outcome; contextual factors can then be incorporated into the evidence base to inform healthcare management and policy making.
- We used experts and stakeholders to facilitate the process, help confirm findings and produce a context-specific document in response to emerging issues.
- Some studies did not describe how general practitioners worked in adequate depth to identify key mechanisms that led to the outcomes.

For peer review only

- We have focussed on general practitioners treating patients in emergency department settings relevant to the UK healthcare system; patient demographics and other healthcare professionals working in primary care services may vary and influence the effectiveness of these services.

For peer review only

The impact of general practitioners working in or alongside emergency departments: a rapid realist review

Background

Worldwide, emergency healthcare systems are under intense pressure from ever increasing demand. (1) Evidence is urgently needed to understand how best to manage this demand whilst safely achieving the highest standards of care.(2) An estimated 10-43% of patients attending hospital emergency departments could be treated in primary care settings.(3–9) In England, this has led to a policy proposal, supported by £100million of funding ($130million USD), that all emergency departments have a co-located primary care facility, so they are "free to care for the sickest patients". (10–12)

The United Kingdom (UK) has a universal healthcare system, the National Health Service (NHS), funded though taxation.(13) Primary care is led by general practitioners, community-based doctors with generalist training. General practitioners are described as working in or alongside emergency departments in three main ways: treating patients identified as having primary care type problems in a unit alongside the emergency department including walk-in centres, urgent care centres or out-of-hours services; treating patients inside the emergency department which may include patients presenting with a wider range of conditions; or working at the front door of the emergency department, redirecting patients with primary care type problems to an alternative primary care service off site (including pharmacists, opticians, or back to their own general practitioner).(14) There is little research evidence to guide decisions about how general practitioners most effectively work within these service models. The risk of provider-induced demand, potential patient safety issues and how to recruit a workforce for this initiative are also unclear.(15–19) Due to this uncertainty, the main standard setting body of the NHS (NICE),

For peer review only

does not currently recommend general practitioners work in emergency department settings.(20)

Research studies addressing these questions are heterogeneous and few are conducted at scale.(15–17) This limits the results of traditional synthesis methods to shape practice or policy. Realist methods offer an alternative approach, generating theories to explain why a particular intervention is likely to work, how, for whom, in what circumstances, and why.(21) These methods identify the important contextual factors that facilitate or inhibit desired intervention outcomes to inform healthcare management and policy making.(22) Urgent and emergency care settings vary in geographical location, the type of patients, the presenting conditions and the experience and disciplines of the healthcare professionals that treat them. We decided that a realist approach, aiming to explain how general practitioners work in or alongside different emergency department settings and why the resultant successes or failures occur, would be more informative than a traditional review approach.

Our research question was, “Why and how do general practitioners working in or alongside emergency departments affect: patient attendance and flow; patient experience; patient safety; and the wider healthcare system?”

For peer review only

Box 1: Glossary of terms

Primary care type problem	A condition that a typical general practitioner in a typical general practice would be expected to manage.
Streaming	A system, following brief clinical assessment, to allocate patients to the most appropriate healthcare provider within the emergency department setting.(23)
Triage	Identifying acuity, and prioritising patients on that basis.(23)
Redirection	"Sending people away" to an appropriate off site or separately managed service.(23)
Context (C)	Pre-existing conditions which influence the success or failure of different interventions or programmes. (21,24)
Mechanism (M)	The intervention and people's reaction to it; how does it influence their reasoning? (21,24)
Outcome (O)	Intended and unintended results as a result of a mechanism operating within a context. (21,24)
Initial theory	An early theory informed by available evidence describing why, how and for whom the intervention is thought to work using a context-mechanism-outcome configuration. (21,24)
Refined theory	An initial theory that has been refined using primary or secondary evidence. (21,24)

Method

We followed the realist review methodology to identify mechanisms (M) that explain how or why contexts (C) relate to outcomes (O), to generate *theories* described as context-mechanism-outcome configurations.(21) (Specific terminology is defined in Box 1.) Our focus was specifically on general practitioners working in or alongside emergency departments. We used the rapid realist review approach described by Saul et al. which utilises experts and stakeholders to streamline the process and to produce a context-specific product that is useful to policy makers and responsive to emerging issues; providing evidence and making explicit what is known on the given topic, also articulating the current research gaps.(25) We registered our protocol on the Prospero database (http://www.crd.york.ac.uk/PROSPERO/display_record.php?ID=CRD42017069741) and

For peer review only

followed RAMESES publication standards for realist reviews.(26) The period of study was April – November 2017.

Three reviewers (AC, FD and ME) conducted a scoping exercise with the four United Kingdom papers identified in the review by Ramlakhan et al.(4,27–29) and two policy documents,(14,30) to generate *initial theories*. We then developed and piloted data extraction forms. Our theories were developed at the *micro-level* (the reasoning processes of general practitioners, emergency department staff and patients), *meso-level* (staff interactions resulting in department level outcomes) and *macro-level* (the impact on the wider system).(31)

We discussed these initial theories with the wider study team of 18 collaborators, including emergency department clinicians, policy makers, general practitioners, members of the public and methodologists at a study meeting in May 2017. We used them as an expert reference group, to contribute ideas for other possible initial theories and to identify further research papers in peer-reviewed journals and relevant reports in the grey literature. Six members of this group (AP, PA, BE, BH, JD, ACS) met via teleconference every six weeks to discuss findings and guide priority search areas.

We used papers referenced in three previous systematic reviews as a starting point,(15–17) and to identify papers published since, we combined search terms used previously.(16,17) A combination of free text and Medical Subject Headings (MeSH) terms was used (see supplementary file 1 for Medline strategy which was adapted for other databases). AC ran the searches on the following databases from 15th June – 4th July 2017: Medline via OVID, Embase, CINAHL, Cochrane DSR & CRCT, DARE, HTA Database, Business Source Complete, PsycINFO and SCOPUS and used Endnote X8 (Clarivate analytics) to export citations from the database searches and identify duplicates. AC screened the titles and

For peer review only - http://bmjopen.bmj.com/site/about/guidelines.xhtml

abstracts of all identified papers using a checklist, developed and tested in collaboration with FD, which ranked abstracts according to relevance.

We selected studies if they could contribute to the process of theory development at the level of individual data extracts rather than assessing the full text against a set checklist.(26,32) We excluded papers that lacked relevance or explanatory power, or were unavailable in English. AC and FD imported data extracts into NVivo 11 (QRS international) that evidenced how mechanisms (M), influenced by local contexts (C), related to outcomes (O). Quantitative, qualitative or contextual data were extracted from any part of a paper. We continually considered the relevance and rigour of each included piece of evidence during the data extraction and synthesis phases.(32) We discussed weekly within the team (AC, FD, ME, AE) how individual data extracts should be used to ensure appropriate inferences were made.(32) A quarter of all included articles was read by both reviewers, and the coding process was discussed in detail, to ensure consistency of approach.

We used snowballing techniques (such as searching companion papers and citation tracking) for all included articles. We also searched to identify additional relevant grey literature (including policy documents and opinion pieces) from a variety of sources. The search process was iterative, overlapping with data extraction and analysis, and was directed towards the evidence gaps and finding explanatory information.

We applied Pawson's reasoning processes,(21) to synthesise the evidence and develop our theories. We presented these context-specific *developing theories* to our expert reference group in November 2017. At this stage, the group recognised that although the review had been useful in theory development, there were limited opportunities for theory testing and refinement due to evidence gaps. Rather than continuing to search the literature, we decided that gathering primary data from our evaluation case study sites in

the next phase of our wider ongoing study,(33) would give more meaningful testing to derive *refined theories*.(21)

Patient and public involvement

Three public contributors (BE, BH, JH) were co-applicants for the funded research and contributed to the conceptualisation of our wider study, including theory generation through the review.(33) They contributed in both meetings described above to ensure that the patient's perspective was acknowledged and at a stakeholder dissemination event in February 2018.

Results

Figure 1 shows the search strategy and results. A total of 96 articles contributed to the developing theories. The articles were largely primary research studies, most from the UK (n= 44 articles), with a large contribution from The Netherlands (n=17). Others were from Ireland, Belgium, Switzerland, Sweden, Italy, Finland, Australia, USA, Canada, Singapore and New Zealand. Most described patients identified by the emergency department as having primary care type problems, appropriate for treatment by a general practitioner.

We synthesised data to develop theories, described using Context (C) - Mechanism (M) - Outcome (O) configurations, to explain: how or why emergency department staff and general practitioners react to guidance to determine which patients are streamed to general practitioners; the role general practitioners may adopt in the emergency department setting (traditional general practitioner, extended general practitioner, gatekeeper or emergency clinician); and how these factors influence patient (experience and safety) and

For peer review only

organisational (risk of provider-induced demand and cost-effectiveness) outcomes. These theories are summarised in Table 1 with an indication of supporting data. Full details of included articles are listed in supplementary file 2.

For peer review only

Table 1: Summary of *developing theories* and supporting evidence

Theory	Context (C) – Mechanism (M) – Outcome (O) Configuration	Example of supporting extract	Evidence base
1. Effectiveness of the streaming system	General practitioners and emergency department staff use their own personal experience and expectation (C) when interpreting streaming guidance (M) to influence which patients are streamed to general practitioners (O).	*"It seems that patients are difficult to classify (for A&E, WiC GP or WiC NP) on limited information for several reasons: serious conditions can sound minor, and vice versa; conditions can present in various ways; and complaints can have several underlying causes."(37)*	Data to support theory (4,14,27,34–42)
2a. Traditional general practitioner role vs emergency clinician role	When general practitioners working in the emergency department maintain a 'traditional role' using the same approach taken in the primary care setting (M) to treat patients with primary care problems (C), investigations, admissions and process times will reduce (O). However, if general practitioners adopt an 'emergency clinician role' working as another pair of hands ('going native') because of their personal interest or experience or because they feel this is the correct way to work in this setting (M), there will be no difference in the rate of investigations and admissions (O).	*"I guess our emergency medicine approach is we're looking for something dreadful and a GP approach is very different in that most of the time they know it's minor stuff or … moderate stuff that is self-limiting and so … they're looking to find symptomatic relief and how can we get this patient home and away from hospital."* (Consultant)(40)<br>*"Once they start becoming like everyone else then they stop being like a GP and they don't necessarily work quickly and effectively which is supposed to be the whole benefit of having them there."* (Consultant)(40)	Data to support traditional GP role theory (4,5,27,28,40,41,43–50)<br><br>Limited data to support ED clinician role theory (40,51)
2b. Extended general practitioner role	General practitioners in emergency departments can work in an 'extended role' where their skills are directed at specific patient groups including non-urgent paediatric or elderly patients (C), to treat using the usual primary care approach (M), to reduce the use of hospital resources and admissions in these patient groups (O).	*"During a 6-month pilot scheme which co-located a primary care GP service in a busy paediatric ED, patients seen during the hours when the GP was available were significantly less likely to be admitted, exceed the 4-hour waiting target or leave before being seen, but more likely to receive antibiotics."(5)*	Data to support theory for paediatric patients only (5,30,52–54)

For peer review only

2c. Gatekeeper role	General practitioners can use their generalist skills and knowledge of community resources (M) to redirect patients presenting with primary care problems (C) out of the emergency department to alternative primary care services off site for treatment thereby reducing emergency department attendances (O).	*"GPs and nurses based in triage identify patients who could be managed more appropriately in primary care as soon as they enter the Emergency Department, and re-direct them back to primary care services."*(55)	Limited data to support theory (55,56)
3. Patient satisfaction	Patients with primary care problems that present to emergency departments (C) and are seen by general practitioners, are more satisfied with the care they receive (O) if the experience exceeds expectation (M), but if they do not perceive any difference in the care they received compared to what they expected (M), there is no difference in satisfaction (O).	*"There were no significant differences in (patient) satisfaction ratings between the three groups of doctors (GPs/SHOs/Registrars)"*(41)	Limited data to support theory (28,41,43,45,57–61)
4. Safety implications	In emergency departments where there are delayed patient transfers to wards or inadequate staffing (C) general practitioners seeing patients with primary care type problems (M), may not free up emergency department staff to care for the sickest patients (O).	*"The attribution of overcrowding in ED to attendance by GP-type patients is simplistic; it does not address how patients are processed within ED or how they are transferred to wards later if required ('access block')."*(44)	Limited data to support theory (44,62–68)
5. Risk of provider-induced demand	If patients with primary care type problems present to emergency departments (C) and are streamed to indistinct primary care services, without patient awareness or choice (M), there is no provider-induced demand (O). However, distinct urgent primary care services may offer convenient access to primary care (M), resulting in provider-induced demand (O).	*"A and E [ED] has not seen any reduction in their patients. If there is a service, patient[s] will use it. You could have three walk-in centres in the city and all three would be used and you may still not see any dropping in A and E [ED] counts."* (Manager)(69)	Data to support theory (4,29,69–80)
6. Cost-effectiveness	If there is demand for patients with primary care problems presenting to emergency departments (C), and they are streamed to on site general practitioners and managed using a traditional general practitioner approach (M), the service is cost-effective due to fewer referrals, admissions, investigations and better outcomes compared to usual services (O).	*"Management of patients with primary care needs in accident and emergency department by general practitioners reduced costs with no apparent detrimental effect on outcome."*(28)	Limited data to support theory (28,43,49)

For peer review only

Theory 1: Effectiveness of the streaming system

General practitioners and emergency department staff use their own personal experience and expectation (C) when interpreting streaming guidance (M) to influence which patients are streamed to general practitioners (O).(4,14,27,34–42)

Twelve articles supported this theory and indicated how the streaming process itself directly influenced the effectiveness of the general practitioner service in the department. Variable streaming rates were described due to differences in guidelines and also how the guidance was interpreted by emergency department clinical and non-clinical staff of varying experience.(34,39,40,42,51) The (streaming) nurse was sometimes described as being unclear which patients general practitioners could deal with,(4,27,36–40) or being more familiar with emergency department work so favouring emergency department referral,(14,35,37,39–41) even overruling the guidelines if he/she felt that the patient would require specific investigations,(37) or admission.(35) General practitioners were also noted to override nurse decisions to select patients that suited their own interests or perceived skills.(81) Increased general practitioner streaming rates were reported when there was a good relationship between the general practitioners and emergency department nurses,(42) and when the general practitioners were directly involved in the streaming process.(53,82) The influence of commissioning or leadership was not described.

Theory 2a: Traditional general practitioner role vs emergency clinician role

When general practitioners working in the emergency department maintain a 'traditional role' using the same approach taken in the primary care setting (M) to treat patients with primary care problems (C),(40,41,43–46) *investigations, admissions and process times will reduce (O).*(4,5,27,28,43,45–50) *However, if general practitioners adopt an 'emergency*

For peer review only - http://bmjopen.bmj.com/site/about/guidelines.xhtml

clinician role' working as another pair of hands ('going native') because of their personal interest or experience or because they feel this is the correct way to work in this setting (M), there will be no difference in the rate of investigations and admissions (O).(40,51)

The traditional general practitioner approach was described by many authors as a different approach to risk management and diagnostic uncertainty, with less reliance on acute investigations.(40,41,44–46) This approach was maintained in a variety of different settings despite full access to investigations – when general practitioners were allocated a separate consulting room mimicking usual general practice,(4,27) and also when general practitioners worked in a more fully integrated model, alongside emergency department clinicians.(43,45,46) Other articles reported general practitioners managing non-urgent patients in this way to divert attendances from emergency department staff. (34,38,39,53,54,74,75,80,82–91)

There were limited qualitative data to support the 'emergency clinician role' theory.(40) An Irish study described an "unstructured receptionist-based triage system" for all patients attending the department (including referrals from primary care) which may have influenced relatively inexperienced general practitioners to adopt a 'diagnosis driven' emergency clinician approach.(51) The influence of general practitioners' special interests, experience in emergency medicine or the effect of staff shortages were not described in the literature to affect this potential role shift.

For peer review only

Theory 2b: Extended general practitioner role

General practitioners in emergency departments can work in an 'extended role' where their skills are directed at specific patient groups including non-urgent paediatric or elderly patients (C) to treat using the usual primary care approach (M) to reduce the use of hospital resources and admissions in these patient groups (O).(5,30,52–54)

Several paediatric primary studies supported general practitioners treating children triaged as 'non-urgent' to divert attendances from the emergency department,(53,54) and reduce hospital admissions.(5,52) None of the included primary studies described general practitioners specifically treating care home residents or the elderly, as suggested in a policy document.(30)

Smith et al. reported an increase in antibiotic prescribing for children by general practitioners,(5) which could potentially be an unintended consequence of the 'traditional role' approach; relying on clinical acumen and treating a suspected source of infection rather than admitting, investigating and observing the patient to confirm the diagnosis. An increase in prescribing by general practitioners was not described in other UK studies,(4,27) but was reported (but not the drugs involved) in both Irish studies that involved more junior general practitioners.(43,51)

There was evidence that general practitioners working in or alongside emergency departments see a different cohort of patients to that in usual general practice, with more acutely unwell patients,(40,92) and minor injuries,(4,6,37–40,93,94) which could also be described as an 'extended role.' There was no evidence in the included studies for the implications of this on their skillset, learning needs, cognition processes or risk management behaviour.

For peer review only

Theory 2c: Gatekeeper role

General practitioners can use their generalist skills and knowledge of community resources (M) to redirect patients presenting with primary care problems (C) out of the emergency department to alternative primary care services off site for treatment thereby reducing emergency department attendances (O). (55,56)

There were limited data to support this theory with two London case study reports identified in an “A&E avoidance” scheme document, describing 228 patients in total.(55,95) There was evidence that general practitioners were more likely to redirect patients after an initial assessment than senior emergency department nurses, but only from a sample of 384 patients that self-presented to a London emergency department.(56)

Due to a lack of evidence for general practitioners performing a redirection role, following realist methodology, we also included studies involving redirection of patients from the emergency department by a senior emergency department clinician or nurse to gain understanding about how and why the system worked. Many of these articles described reduced emergency department attendances.(96–102) Previous UK guidance has cautioned against redirecting patients from emergency departments due to the risk of delayed assessment and treatment, especially in vulnerable patient groups including the homeless or those with mental health problems who may not go on to receive the care they need.(14,30) Studies from Scotland, Sweden and the United States that described a comprehensive assessment process, including measurement of vital signs and a focussed history, reported that their redirection policies were safe and worked well to reduce attendances.(78,97,99,101,102) Other United States studies, that did not describe the assessment process, reported adverse events when children were redirected without treatment.(103,104) The low sensitivity of triage criteria to identify those that needed

For peer review only

urgent care,(105) especially infants,(106) and failure to validate a predictive model for refusal of care,(107) were highlighted in other studies. The influence of governance processes restricting redirection of patients by some staff to services off site was not described in these articles.

Theory 3: Patient satisfaction

Patients with primary care problems that present to emergency departments (C) and are seen by general practitioners, are more satisfied with the care they receive (O) if the experience exceeds expectation (M), but if they do not perceive any difference in the care they received compared to what they expected (M), there is no difference in satisfaction (O).(28,41,43,45,57–61)

Data to support this theory were limited, with an increase in satisfaction by patients seen by general practitioners generally associated with shorter waiting times,(45,57) rather than expectation of investigation and treatment.(41) The general practitioners were sometimes supernumerary which may have contributed towards this.(28,45) Other studies demonstrated that general practitioners focused more on patient education and counselling than emergency department clinicians with some improvement in satisfaction rates.(108,109) In more fully integrated models, the patient was often unaware that they had seen a general practitioner rather than an emergency department clinician and there was no difference in patient satisfaction.(28,41,43,58)

Theory 4: Safety implications

In emergency departments where there are delayed patient transfers to wards or inadequate staffing (C) general practitioners seeing patients with primary care type

For peer review only

problems (M), may not free up emergency department staff to care for the sickest patients (O).(44,62–68)

There was a lack of evidence that general practitioners working in or alongside emergency departments directly or indirectly improved care and safety for the sickest patients. A reduction in time spent in the department for patients requiring emergency department level care was suggested in a UK simulation and modelling study,(62) and an Australian study also reported a reduced mean time taken to see more seriously ill patients but this was not seen on sites that described provider-induced demand.(63) A Canadian study of over 4 million patient visits reported that low complexity emergency department patients did not increase time to first physician contact for high-complexity patients.(64) Other studies also described how diverting non-urgent patients did not improve the high level care required by others, and that influences such as delayed transfer of patients to the ward were more likely to contribute to overcrowding.(44,65–67) Staffing levels, staff attitude and the time of day were independent factors described to affect emergency department flow.(68)

There were minimal data on the safety implications of general practitioners working in emergency department settings. Several studies used emergency department re-attendance as a marker of safety, with no increase among patients seen by general practitioners compared to usual emergency department staff. (28,29,43,110,111) Annual death rates were used as another crude marker in a Dutch study, with no significant increase following the introduction of an out-of-hours primary care physician cooperative.(75) Shared or separate governance systems between general practitioners and the emergency department were rarely described in the primary studies, providing no evidence for best practice. For general practitioners working inside the emergency

For peer review only

department good communication and integration were described in some studies,(4,28,40,92) with anecdotal reports of poor communication negatively affecting care quality in others.(34)

Theory 5: The risk of provider-induced demand

If patients with primary care type problems present to emergency departments (C) and are streamed to indistinct primary care services, without patient awareness or choice (M), there is no provider-induced demand (O).(4,29,74,75) *However, distinct urgent primary care services may offer convenient access to primary care (M), resulting in provider-induced demand (O).*(69–73,76–80)

Four articles described fully integrated models, where non-urgent patients were streamed directly to general practitioners inside the emergency department without provider-induced demand.(4,29,74,75). Here, there was no patient choice offered and often a lack of patient awareness. Another 10 articles described distinct urgent primary care services, often in separate buildings outside the emergency departments, as duplicating services and creating their own demand, increasing patient presentation rates directly or at nearby services, rather than relieving pressure on the emergency department.(69–73,76–79,112–114)

Theory 6: Cost-effectiveness

If there is demand for patients with primary care problems presenting to emergency departments (C), and they are streamed to on site general practitioners and managed using a traditional general practitioner approach (M), the service is cost-effective due to fewer

For peer review only - http://bmjopen.bmj.com/site/about/guidelines.xhtml

referrals, admissions, investigations and better outcomes compared to usual services (O).(28,43,49)

Data to support this theory were limited, but supported by three economic evaluations (UK, Ireland and The Netherlands) where non-urgent patients were streamed to general practitioners during normal daytime hours.(28,43,49) The comparator was 'business as usual' with no general practitioner service. The UK and Irish studies were published in 1996 and may not represent current emergency department staffing models. No articles were identified that studied the relative cost-effectiveness of general practitioners redirecting patients from the emergency department for care elsewhere. A 5-year USA redirection study calculated cost-effectiveness from the perspective of the institution but did not include costs for treatment incurred elsewhere,(99) whilst another United States study calculated that marginal costs for non-urgent visits to the emergency department were low and that cost savings from diverting visits may be less than widely believed.(115) However the USA has a complex health system, with a significant majority of the population covered by private health insurance alongside state funded Medicare, Medicaid, the Federally-funded Veterans Health Administration, and a substantial uninsured population - all factors which could influence access to emergency departments and the type of care needed and delivered.

Three other studies of 'out-of-hours' patients did not find the addition of a primary care service to be cost saving. One Dutch study, with an off-site general practitioner co-operative, reported parents refusing to take the child to a different location, or the (streaming) nurse overruling the policy.(35) Another 12 year old Dutch study showed no change in costs, despite a substantial reduction in emergency department attendances, due to regulations dictating minimum staffing levels to cope with major trauma.(91) The Dutch

For peer review only

health care system has a complex funding structure with a mix of social and private insurance and this may influence incentives and disincentives to access emergency departments. An Australian primary care out-of-hours service closed because patients chose to attend an equally accessible general practice service that existed nearby.(42)

Wider system implications

Limited evidence from the included studies prevented us developing theories on wider system implications. There were no reports of emergency department clinicians encouraged to adopt a more conservative approach, as a result of working alongside general practitioners, but some reports of general practitioners in management positions influencing system changes.(116,117) The potential reduction in learning opportunities for junior doctors was highlighted in two articles.(50,92) There was limited evidence that working in an emergency department setting led to increased job satisfaction for some UK general practitioners with a special interest in emergency care.(40,117) However, reduced satisfaction was also described because the job was outside the scope of usual general practice,(40,48) possibly contributing towards recruitment problems.(40,118)

Discussion

Principal findings

We developed theories using data from 96 articles to describe the mechanisms by which general practitioner services are linked to outcomes: about the streaming process itself; the role general practitioners may adopt in the emergency department setting; and the effects of these on the patient (experience and safety) and the organisation (risk of

For peer review only

provider-induced demand and costs). There was little evidence that general practitioners in emergency departments directly or indirectly affected the care and throughput of the sickest patients. Distinct units, advertising these services, may offer an attractive alternative to primary care and result in provider-induced demand. The literature describing economic impacts of general practitioners in emergency departments comes from different countries, with different funding systems and spans over 20 years, limiting conclusions.

Strengths and limitations

Heterogeneous studies involving general practitioners working in or alongside emergency departments do not suit traditional systematic review methods. We have conducted the first realist review in this area, using methods that are gaining prominence in healthcare research.(119,120) The rapid realist review approach is appropriate in relation to the rapidly evolving NHS policy on emergency department use of general practitioners, (10–12) showing where such policies may be reinforced or refuted by the evidence available.(25) A weakness of our study was the time constraint on our project but the expert group mitigated this, and enabled us to focus and direct our research.(25) Some studies did not describe the intervention in adequate depth to help facilitate the identification of key mechanisms. Single site heterogeneous studies and the nature of different healthcare and funding systems limited international comparability.(21)

The wide estimates of patients presenting with primary care type problems to emergency departments highlights the difficulty in defining and identifying this target patient group and therefore the effectiveness of these services in different local contexts. We have focussed on general practitioners working in or alongside emergency departments but in the UK this role has evolved to include nurses and advanced care practitioners from

For peer review only

other disciplines, often due to staffing and recruitment challenges. These challenges may be mirrored in emergency department based services, affecting variation between services and need to be considered in further research.

Comparison with other reviews

Before our review, the largest review to date by Ramlakhan et al. (2016) included 20 papers and described provider-induced demand, poor evidence for improved emergency department throughput and minimal economic impact.(17) The Goncalves-Bradley et al. Cochrane review of four studies, published in 2018, highlighted inconsistent results and a lack of evidence on safety.(18) We also found evidence of provider-induced demand in distinct primary care units but less so in more fully integrated service models where patients lacked awareness that they had been directed to primary care services.(4,74,75) We found that patients with primary care problems may have reduced process times if treated by general practitioners adopting a traditional role but there was a lack of evidence for an improvement in overall throughput for patients in the department. There was also a lack of evidence on the impact on general practitioners' cognition processes and risk-taking behaviour when treating a different group of patients to that seen in usual general practice and the safety implications of this.

Policy implications

The global health priority recently given to Universal Health Coverage,(121) and the attention being given to the 40th anniversary of the Alma Ata declaration,(122) moves to centre stage the design of primary healthcare systems, particularly their capacity and capability to respond to urgent care needs. Internationally, emergency departments are

For peer review only

exploring options on how to run more efficiently and safely. Our theories, informed by literature from 13 countries, allow policy makers to make more considered judgements about their relevance to their own contexts for service provision. The UK has already commissioned further research in this area, funded by the National Institute for Health Research (HS&DR Projects: 15/145/04(33) and 15/145/06(123)), the former collecting primary data to further test and refine these theories.

Conclusion

The effectiveness of emergency department streaming to primary care services may be influenced by how staff interpret the streaming system and the roles general practitioners adopt. Caution is needed in embedding the policy until further research and evaluation are available. Service models that encourage the traditional general practitioner approach may have shorter process times for non-urgent patients; however, there is little evidence that this frees up emergency department staff to care for the sickest patients. Distinct primary care services offering increased patient choice may result in provider-induced demand. Economic evaluation and safety requires further research.

Figure 1: Search Strategy and Results

For peer review only

Acknowledgements

Many thanks to our public contributors, Barbara Harrington and Julie Hepburn, and to Professor Damon Berridge for contributing to discussions. Also to Faris Hussain for assisting with citation tracking.

Contributors

Authors AC, FD, PA, ACS, MC, LD, JD, BE, PH, TH, AP, TR, AS, HS and AE are co-applicants on the wider project and were involved in the conceptualisation of the study. AC, FD, ME, PA, ACS, MC, JD, BE, PH, TH, AP, TR, AS, HS and AE contributed as part of the expert group in team meetings in May and/or November 2017. AC and FD planned the synthesis approach with input from AE. ME contributed to data analysis and interpretation in the pilot work and weekly team meetings. The core review team (AP, PA, BE, JD and ACS) met via teleconference every 6 weeks to discuss findings and guide further searches. AC conducted the database searches. AC and FD extracted data extracts and were involved in the synthesis process, meeting weekly with AE and ME to discuss findings. AC prepared the first draft of the manuscript which was reviewed and critically appraised by all authors, who approved the final version and agree to be accountable for this work.

Funding

This work was supported by the National Institute for Health Research HS&DR Project 15/145/04

Competing interests

None declared

For peer review only

Data sharing statement

No further data available

For peer review only

References

1. What's going on with A&E waiting times? The King's Fund https://www.kingsfund.org.uk/projects/urgent-emergency-care/urgent-and-emergency-care-mythbusters
2. Keogh B. Transforming urgent and emergency services in England: Urgent and emergecy care review 2013. http://www.nhs.uk/NHSEngland/keogh-review/Pages/urgent-and-emergency-care-review.aspx
3. Emergency departments: More useful than the official data suggests The College of Emergency Medicine. 2014. http://www.kingstoned.org/uploads/2/4/0/2/24023085/ca_past_paper.pdf
4. Ward P, Huddy J, Hargreaves S, Touquet R, Hurley J, Fothergill J. Primary care in London: an evaluation of general practitioners working in an inner city accident and emergency department. J Accid Emerg Med. 1996;13(table 1):11–5.
5. Smith L, Narang Y, Ibarz Pavon AB, *et al* To GP or not to GP: a natural experiment in children triaged to see a GP in a tertiary paediatric emergency department (ED) BMJ Qual Saf 2018;27:521-528
6. Dale J, Green J, Reid F, Glucksman E. Primary care in the accident and emergency department: I. Prospective identification of patients. BMJ. 1995;311(7002):423-6
7. Coleman P, Irons R, Nicholl J. Will alternative immediate care services reduce demands for non-urgent treatment at accident and emergency? Emerg Med J. 2001;18(6):482–7.
8. Nagree Y, Camarda V, Fatovich D, Cameron P, Dey I, Gosbell A, et al. Quantifying the proportion of general practice and low-acuity patients in the emergency department. 2013;198:612–5.
9. Thompson MI, Lasserson D, McCann L, Thompson M, Heneghan C. Suitability of emergency department attenders to be assessed in primary care: survey of general practitioner agreement in a random sample of triage records analysed in a service evaluation project. BMJ open. 2013;3(12):e003612
10. Acute and emergency care: prescribing the remedy 2014. https://www.rcplondon.ac.uk/news/acute-and-emergency-care-prescribing-remedy
11. A&E departments to get more funding - GOV.UK https://www.gov.uk/government/news/ae-departments-to-get-more-funding
12. Next steps on the NHS five year forward view 2017. https://www.england.nhs.uk/wp-content/uploads/2017/03/NEXT-STEPS-ON-THE-NHS-FIVE-YEAR-FORWARD-VIEW.pdf
13. The principles and values of the NHS in England Department of Health https://www.nhs.uk/NHSEngland/thenhs/about/Pages/nhscoreprinciples.aspx
14. Carson D, Clay H, Stern R, Primary care and emergency departments. Primary Care Foundation. 2010
15. Khangura JK, Flodgren G, Perera R, Rowe B, Shepperd S. Primary care professionals providing non-urgent care in hospital emergency departments. The Cochrane Library. 2012;53(11):1689–99.
16. Turner J, Coster J, Chambers D, Cantrell A, Phung V-H, Knowles E, et al. What evidence is there on the effectiveness of different models of delivering urgent care? A rapid review. Heal Serv Deliv Res 2015;3(43):1–134.
17. Ramlakhan S, Mason S, O'Keeffe C, Ramtahal A, Ablard S, Burke D, et al. Primary care services located with EDs: a review of effectiveness. Emerg Med J 2016;33(7):495–503.
18. Goncalves-Bradley D, Khangura JK, Flodgren G, Perera R, Rowe BH, Shepperd S. Primary care professionals providing non-urgent care in hospital emergency departments. The Cochrane Library 2018
19. Baird B, Charles A, Honeyman M, Maguire D, Das P. Understanding pressures in general practice The King's Fund. 2016. https://www.kingsfund.org.uk/sites/files/kf/field/field_publication_file/Understanding-GP-pressures-Kings-Fund-May-2016.pdf
20. NICE guideline GPs within or on the same site as emergency departments. 2017;(July)
21. Pawson R, Tilley N. Realistic evaluation. SAGE Publications; 2008.
22. Lavis J, Davies H, Oxman A, Denis JL, Golden-Biddle K, Ferlie E. Towards systematic reviews that inform health care management and policy-making. Journal of health services research & policy. 2005;10(1_suppl):35-48
23. RCEM: Initial Assessment of Emergency Department Patients 2017 https://www.rcem.ac.uk/docs/SDDC Intial Assessment (Feb 2017).pdf
24. Rycroft-Malone J, Burton CR, Williams L, Edwards S, Fisher D, Hall B, et al. Improving skills and care

standards in the support workforce for older people: a realist synthesis of workforce development interventions NIHR HS&DR. 2016. https://www.ncbi.nlm.nih.gov/books/NBK355884/pdf/Bookshelf_NBK355884.pdf
25. Saul JE, Willis CD, Bitz J, Best A. A time-responsive tool for informing policy making: rapid realist review. Implementation Science. 2013;8(1):103.
26. Wong G, Greenhalgh T, Westhorp G, Buckingham J, Pawson R. RAMESES publication standards: realist syntheses. BMC medicine. 2013;11(1):21
27. Dale J, Green J, Reid F, Glucksman E, Higgs R. Primary care in the accident and emergency department: II. Comparison of general practitioners and hospital doctors. BMJ. 1995;311(7002):427-30.
28. Dale J, Lang H, Roberts JA, Green J, Glucksman E. Cost effectiveness of treating primary care patients in accident and emergency: a comparison between general practitioners, senior house officers, and registrars. BMJ. 1996;312(7042):1340-4
29. Salisbury C, Hollinghurst S, Montgomery A, Cooke M, Munro J, Sharp D, et al. The impact of co-located NHS walk-in centres on emergency departments. Emerg Med J. 2007;24(4):265–9.
30. Primary Care in Emergency Departments A guide to good practice. NHS Interim Mangement and Support. 2015 http://www.nhsimas.nhs.uk/fileadmin/Files/IST/Emergency_care_conference_2014/Primary_Care_in_A_E_Guidance_Feb_2015.pdf
31. Barach P, Johnson JK. Understanding the complexity of redesigning care around the clinical microsystem. BMJ Qual & Saf. 2006;15(suppl 1):i10-6.
32. Pawson R. Evidence-Based Policy. SAGE Publications Ltd; 2006.
33. Edwards A. Evaluating effectiveness, safety, patient experience and system implications of different models of using GPs in or alongside Emergency Departments. https://www.journalslibrary.nihr.ac.uk/programmes/hsdr/1514504/#/
34. Thijssen W a MH, Wijnen-van Houts M, Koetsenruijter J, Giesen P, Wensing M. The impact on emergency department utilization and patient flows after integrating with a general practitioner cooperative: an observational study. Emerg Med Int 2013;2013:364659. http://www.pubmedcentral.nih.gov/articlerender.fcgi?artid=3814098&tool=pmcentrez&rendertype=abstract
35. Van Veen M, Wolde F, Poley M, Ruige M, Meurs A, Hable C, et al. Referral of nonurgent children from the emergency department to general practice: compliance and cost savings. Eur J Emerg Med. 2012;19:14–9.
36. Bickerton J. Streaming A&E patients to walk-in centre services. Emerg nurse. 2005;13(3):20–4.
37. Van der Straten LM, van Stel HF, Spee FJ, Vreeburg ME, Schrijvers AJ, Sturms LM. Safety and efficiency of triaging low urgent self-referred patients to a general practitioner at an acute care post: an observational study. Emerg Med J. 2012;29(11):877-81.
38. Chmiel C, Wang M, Sidler P, Eichler K, Rosemann T, Senn O. Implementation of a hospital-integrated general practice-a successful way to reduce the burden of inappropriate emergency-department use. Swiss Med Wkly. 2016;146:w14284
39. Van Gils-van Rooij ES, Meijboom BR, Broekman SM, Yzermans CJ, de Bakker DH. Is patient flow more efficient in Urgent Care Collaborations?. European Journal of Emergency Medicine. 2018;25(1):58-64.
40. Ablard S, O'keeffe C, Ramlakhan S, Mason SM. Primary care services co-located with Emergency Departments across a UK region: early views on their development. Emerg Med J. 2017;34(10):672-6.
41. Dale J. Primary Care in Accident and Emergency Departments : the cost effectiveness and applicability of a new model of care. 1998;1–242.
42. Bolton P, Thompson L. The reasons for, and lessons learned from, the closure of the Canterbury GP After-Hours Service. Aust Health Rev 2001;24(3):66–73.
43. Murphy A, Bury G, Plunkett PK, Gibney D, Smith M, Mullan E, et al. Randomised controlled trial of general practitioner versus usual medical care in an urban accident and emergency department: process, outcome, and comparative cost. BMJ. 1996;312(May):1135–42.
44. Wilson H. Co-locating primary care facilities within emergency departments: Brilliant innovation or unwelcome intervention into clinical care? N Z Med J. 2005;118(1221):1–11.
45. Boeke AJP, Van Randwijck-Jacobze ME, De Lange-Klerk EMS, Grol SM, Kramer MHH, Van Der Horst HE. Effectiveness of GPs in accident and emergency departments. Br J Gen Pract. 2010;60(579):378–84.
46. Wang M, Wild S, Hilfiker G, Chmiel C, Sidler P, Eichler K, et al. Hospital-integrated general practice: A promising way to manage walk-in patients in emergency departments. J Eval Clin Pract. 2014;20(1):20–6.

For peer review only - http://bmjopen.bmj.com/site/about/guidelines.xhtml

47. Huibers L, Thijssen W, Koetsenruijter J, Giesen P, Grol R, Wensing M. GP cooperative and emergency department: An exploration of patient flows. J Eval Clin Pract. 2013;19(2).
48. Kool RB, Homberg DJ, Kamphuis HCM. Towards integration of general practitioner posts and accident and emergency departments: a case study of two integrated emergency posts in the Netherlands. BMC Health Serv Res. 2008;8:225.
49. Bosmans JE, Boeke AJ, van Randwijck-Jacobze ME, Grol SM, Kramer MH, van der Horst HE, van Tulder MW. Addition of a general practitioner to the accident and emergency department: a cost-effective innovation in emergency care. Emerg Med J. 2012;29(3):192-6.
50. Hess S, Sidler P, Chmiel C, Bögli K, Senn O, Eichler K. Satisfaction of health professionals after implementation of a primary care hospital emergency centre in Switzerland: A prospective before–after study. International emergency nursing. 2015;23(4):286-93.
51. Gibney D, Murphy AW, Barton D, Byrne C, Smith M, Bury G, et al. Randomized controlled trial of general practitioner versus usual medical care in a suburban accident and emergency department using an informal triage system. Br J Gen Pract. 1999;49(438):43–4.
52. Gritz A, Sen A, Hiles S, Mackenzie G. More under-fives now seen in urgent care centre than a&e-should we shift our focus? Archives of Disease in Childhood. (Annual Conference of the Royal College of Paediatrics and Child Health, RCPCH 2016. United Kingdom.; vol. 101). http://ovidsp.ovid.com/ovidweb.cgi?T=JS&PAGE=reference&D=emed18&NEWS=N&AN=612211417
53. Gnani S, Morton S, Ramzan F, Davison M, Ladbrooke T, Majeed A, Saxena S. Healthcare use among preschool children attending GP-led urgent care centres: a descriptive, observational study. BMJ open. 2016;6(6):e010672
54. Clancy E, Mayo A. Launching a social enterprise see-and-treat service. Emerg Nurse. 2009;17(3):22–4.
55. Proctor N, Woodward M, Partnership HL. A & E Avoidance Schemes across London A rapid review of good practice examples. 2016.
56. Harris T, McDonald K. How do clinicians with different training backgrounds manage walk-in patients in the ED setting? Emerg Med J 2014;31(12):975–9.
57. Arain M, Nicholl J, Campbell M. Patients' experience and satisfaction with GP led walk-in centres in the UK; a cross sectional study. BMC health services research. 2013;13(1):142
58. Chalder M, Montgomery A, Hollinghurst S, Cooke M, Munro J, Lattimer V, Sharp D, Salisbury C. Comparing care at walk-in centres and at accident and emergency departments: an exploration of patient choice, preference and satisfaction. Emergency Medicine Journal. 2007;24(4):260-4
59. Hunter C, Chew-Graham C, Langer S, Stenhoff A, Drinkwater J, Guthrie E, Salmon P. A qualitative study of patient choices in using emergency health care for long-term conditions: the importance of candidacy and recursivity. Patient education and counseling. 2013;93(2):335-41
60. Rajpar SF, Smith M a, Cooke MW. Study of choice between accident and emergency departments and general practice centres for out of hours primary care problems. J Accid Emerg Med. 2000;17:18–21.
61. Hutchison B, Østbye T, Barnsley J, Stewart M, Mathews M, Campbell MK, Vayda E, Harris SB, Torrance-Rynard V, Tyrrell C. Patient satisfaction and quality of care in walk-in clinics, family practices and emergency departments: the Ontario Walk-In Clinic Study. Canadian Medical Association Journal. 2003;168(8):977-83
62. Lengu D, Kobbacy K, Sapountzis S, Kagioglou M. Application Of Simulation And Modelling In Managing Unplanned Healthcare Demand Conference paper University of Salford, Manchester. 2012. http://usir.salford.ac.uk/29360/1/D_Lengu_et_al_-_Application_of_simulation_and_modelling_in_managing_unplanned_healthcare_demand.pdf
63. Sharma A, Inder B. Impact of co-located general practitioner (GP) clinics and patient choice on duration of wait in the emergency department. Emerg Med J 2011;28(8):658–61.
64. Schull MJ, Kiss A, Szalai JP. The effect of low-complexity patients on emergency department waiting times. Annals of emergency medicine. 2007;49(3):257-64
65. Vertesi L. Does the Canadian Emergency Department Triage and Acuity Scale identify non-urgent patients who can be triaged away from the emergency department? Can J Emerg Med. 2004;6(5):337–42.
66. Richardson DB, Mountain D. Myths versus facts in emergency department overcrowding and hospital access block. Med J Aust. 2009;190(7):369–74.
67. Allen P, Cheek C, Foster S, Ruigrok M, Wilson D, Shires L. Low acuity and general practice-type presentations to emergency departments: A rural perspective. Emergency Medicine Australasia. 2015;27(2):113-8
68. O'Cathain A, Knowles E, Turner J, Hirst E, Goodacre S, Nicholl J, et al. Variation in avoidable emergency

admissions: multiple case studies of emergency and urgent care systems. J Health Serv Res Policy 2016;21(1):5–14.
69. Arain M, Baxter S, Nicholl J. Perceptions of healthcare professionals and managers regarding the effectiveness of GP-led walk-in centres in the UK. BMJ Open 2015;5(8):e008286.
70. Kork A, Vakkuri J. Improving access and managing healthcare demand with walk-in clinic Convenient, but at what cost? Int J Public Sect Manag. 2016;29(2):148–63.
71. Greenfield G, Ignatowicz A, S G, Bucktowonsing M, Ladbrooke T, Millington H, et al. Staff perceptions on patient motives for attending GP-led urgent care centres in London: A qualitative study. BMJ Open 2016;6(1):e007683.
72. Tammes P, Morris RW, Brangan E, Checkland K, England H, Huntley A, et al. Exploring the relationship between general practice characteristics, and ttendance at walk-in centres, minor injuries units and EDs in England 2012/2013: A cross-sectional study. Emerg Med J 2016;33(10):702–8.
73. Colliers A, Remmen R, Streffer ML, Michiels B, Bartholomeeusen S, Monsieurs KG, Goris J, Coenen S, Verhoeven V, Philips H. Implementation of a general practitioner cooperative adjacent to the emergency department of a hospital increases the caseload for the GPC but not for the emergency department. Acta Clinica Belgica. 2017;72(1):49-54.
74. Van Uden C, Crebolder H. Does setting up out of hours primary care cooperatives outside a hospital reduce demand for emergency care? Emerg Med J. 2004;21(6):722–3.
75. Van Uden C, Winkens R, Wesseling G, Fiolet H, Van Schayck O, Crebolder H. The impact of a primary care physician cooperative on the caseload of an emergency department: The Maastricht integrated out-of-hours service. J Gen Intern Med. 2005;20(7):612–7.
76. Krakau I, Hassler E. Provision for clinic patients in the ED produces more nonemergency visits. Am J Emerg Med 1999;17(1):18–20.
77. Hsu RT, Lambert PC, Dixon-Woods M, Kurinczuk JJ. Effect of NHS walk-in centre on local primary healthcare services: before and after observational study. BMJ 2003;326(7388):530.
78. Anantharaman V. Impact of health care system interventions on emergency department utilization and overcrowding in Singapore. Int J Emerg Med 2008;1(1):11–20.
79. Maheswaran R, Pearson T, Jiwa M. Repeat attenders at National Health Service walk-in centres - a descriptive study using routine data. Public Health 2009;123(7):506–10.
80. O'Kelly F, Teljeur C, Carter I, Plunkett P. Impact of a GP cooperative on lower acuity emergency department attendances. Emerg Med J 2010;27(10):770–3.
81. Freeman GK, Meakin RP, Lawrenson RA, Leydon GM, Craig G. Primary care units in A and E departments in North Thames in the 1990s: Initial experience and future implications. Br J Gen Pract. 1999;49(439):107–10.
82. Cowling TE, Ramzan F, Ladbrooke T, Millington H, Majeed A, Gnani S. Referral outcomes of attendances at general practitioner led urgent care centres in London, England: retrospective analysis of hospital administrative data. Emerg Med J. 2016;33(3):200-7.
83. Van Gils-Van Rooij ESJ, Yzermans CJ, Broekman SM, Meijboom BR, Welling GP, De Bakker DH. Out-of-Hours Care Collaboration between general practitioners and hospital emergency departments in the Netherlands. J Am Board Fam Med. 2015;28(6):807–15.
84. Van Veelen M, Van den Brand C, Reijnen R, Van der Linden M. Effects of a general practitioner cooperative co-located with an emergency department on patient throughput. World J Emerg Med 2016;7(4):270–3.
85. Arain M, Campbell MJ, Nicholl JP. Impact of a GP-led walk-in centre on NHS emergency departments. Emerg Med J 2015;32(4):295-300.
86. van Uden CJT, Winkens RAG, Wesseling GJ, Crebolder HFJM, van Schayck CP. Use of out of hours services: a comparison between two organisations. Emerg Med J 2003;20(2):184–7.
87. Johnson SJ. Evidence for primary care services at A&E. BMJ [Internet]. 2015;350:h3352.
88. Grant C, Nicholas R, Moore L, Salisbury C. An observational study comparing quality of care in walk-in centres with general practice and NHS Direct using standardised patients. BMJ 2002;324(7353):1556.
89. Schols A, Stevens F, Zeijen C, Dinant G, van Vugt C, Cals J. Access to diagnostic tests during GP out-of-hours care: A cross-sectional study of all GP out-of-hours services in the Netherlands. Eur J Gen Pract [Internet]. 2016;22(3):176–81.
90. Posocco A, Scapinello MP, De Ronch I, Castrogiovanni F, Lollo G, Sergi G, Tomaselli I, Tonon L, Solmi M, Pescador D, Battistuz E. Role of out of hours primary care service in limiting inappropriate access to emergency department. Internal and emergency medicine. 2018;13(4):549-55.
91. Van Uden C, Ament A, Voss G, Wesseling G, Winkens R, van Schayck O, et al. Out-of-hours primary

For peer review only

care. Implications of organisation on costs. BMC Fam Pract. 2006;7:29.
92. Morton S, Igantowicz A, Gnani S, Majeed A, G. G. Describing team development within a novel GP-led urgent care centre model: A qualitative study. BMJ Open 2016;6(6):e010224.
93. Giesen P. Patients either contacting a general practice cooperative or accident and emergency department out of hours: a comparison. Emerg Med J 2006;23(9):731–4.
94. Chmiel C, Huber CA, Rosemann T, Zoller M, Eichler K, Sidler P, et al. Walk-ins seeking treatment at an emergency department or general practitioner out-of-hours service: a cross-sectional comparison. BMC Health Serv Res. 2011;11(1):94.
95. Begum F, Khan H, Moss P. Solving the A & E crisis using GP lead triage and redirection Evaluation of GP-lead service to identify and re-direct patients from A & E to primary care services. 2016;83. https://www.myhealth.london.nhs.uk/sites/default/files/30. Solving the A%26E crisis using GP lead triage and redirection_0.pdf
96. Hansagi H, Carlsson B, Olsson M, Edhag O. Trial of a method of reducing inappropriate demands on a hospital emergency department. Public Health. 1987;101(2):99–105.
97. Rivara F, Wall H, Worley P, James K. Pediatric nurse triage: it's efficacy, safety and implications for care. J Pediatr Heal Care. 1991;5(6):291–8.
98. Derlet RW, Nishio D, Cole LM, Silva J. Triage of patients out of the emergency department: Three-year experience. Am J Emerg Med. 1992;10(3):195–9.
99. Derlet RW, Kinser D, Ray L, Hamilton B, McKenzie J. Prospective Identification and Triage of Nonemergency Patients Out of an Emergency Department: A 5-Year Study. Ann Emerg Med. 1995;25(2):215–23.
100. McGugan E. Primary care or A&E? A study of patients redirected from an accident and emergency department. Scott Med J. 2000;45(5):144–6.
101. Ellbrant J, Akeson J, Akeson PK, J. E, J. A. Pediatric emergency department management benefits from appropriate early redirection of nonurgent visits. Pediatr Emerg Care [Internet]. 2015;31(2):95–100.
102. Bentley JA, Thakore S, Morrison W, Wang W. Emergency Department redirection to primary care: a prospective evaluation of practice. Scott Med J 2017;62(1):2–10.
103. Shaw KN, Selbst SM, Gill FM. Indigent children who are denied care in the emergency department. Ann Emerg Med. 1990;19(1):59–62.
104. Gadomski AM, Perkis V, Horton L, Cross S, Stanton B. Diverting Managed Care Medicaid Patients From Pediatric Emergency Department Use. Pediatrics 1995;95(2):170–8.
105. Lowe RA, Bindman AB, Ulrich SK. Refusing care to emergency department patients: evaluation of published triage guidelines. Ann Emerg Med 1994;23:286–93.
106. Van Veen M, Steyerberg EW, Lettinga L, Ruige M, van Meurs AHJ, van der Lei J, et al. Safety of the Manchester Triage System to identify less urgent patients in paediatric emergence care: a prospective observational study. Arch Dis Child 2011;96(6):513–8.
107. Birnbaum A, Gallagher EJ, Utkewicz M, Gennis P, Carter W. Failure to Validate a Predictive Model for Refusal of Care to Emergency-department Patients. Acad Emerg Med. 1994;1(3):213–7.
108. Dale J, Sandhu H, Lall R, Glucksman E. The patient, the doctor and the emergency department: A cross-sectional study of patient-centredness in 1990 and 2005. Patient Educ Couns. 2008;72(2):320–9.
109. Sandhu H, Dale J, Stallard N, Crouch R, Glucksman E. Emergency nurse practitioners and doctors consulting with patients in an emergency department: a comparison of communication skills and satisfaction. Emerg Med J 2009;26:400–4.
110. Murphy AW, Plunkett PK, Bury G, Leonard C, al et. Effect of patients seeing a general practitioner in accident and emergency on their subsequent reattendance: Cohort study. Br Med J 2000;320(7239):903–4.
111. Doran KM, Colucci AC, Hessler RA, Ngai CK, Williams ND, Wallach AB, et al. An Intervention Connecting Low-Acuity Emergency Department Patients With Primary Care: Effect on Future Primary Care Linkage. Ann Emerg Med. 2013;61(3):312–321.e7.
112. Salisbury C, Chalder M, Scott TM, Pope C, Moore L. What is the role of walk-in centres in the NHS? BMJ 2002;324(7334):399–402.
113. Desborough J, Parker R, Forrest L. Development and implementation of a nurse-led walk-in centre: evidence lost in translation? J Health Serv Res Policy 2013;18(3):174–8.
114. Pope C, Chalder M, Moore L, Salisbury C. What do other local providers think of NHS walk-in centres? Results of a postal survey. Public Health. 2005;119(1):39–44.
115. Williams RM. The costs of visits to emergency departments. N Engl J Med 1996;334(10):642–6.
116. Chew-Graham C, Rogers A, May C, Sheaf R, Ball E. A new role for the general practitioner? Reframing

For peer review only - http://bmjopen.bmj.com/site/about/guidelines.xhtml

'inappropriate attenders' to inappropriate services. Prim Heal Care Res Dev 2004;(5):60–7.
117. Dale J, Russell R, Harkness F, Wilkie V, Aiello M. Extended training to prepare GPs for future workforce needs: a qualitative investigation of a 1-year fellowship in urgent care. Br J Gen Pract 2017;67(662):e659–67.
118. Van den Heede K, Quentin W, Dubois C, Devriese S, Van de Voorde C. The 2016 proposal for the reorganisation of urgent care provision in Belgium: A political struggle to co-locate primary care providers and emergency departments. Health Policy 2017;121(4):339–45.
119. Greenhalgh T, Humphrey C, Hughes J, MacFarlane F, Butler C, Pawson R. How Do You Modernize a Health Service? A Realist Evaluation of Whole-Scale Transformation in London. Milbank Q. 2009;87(2):391–416.
120. Gillespie BM, Marshall A, Baker R, Cleary K, Davies H, Davies S. Implementation of safety checklists in surgery: a realist synthesis of evidence. Implement Sci 2015;10(1):137.
121. Report of the Expert Consultation on Primary Care Systems Profiles & Performance (Primasys). The Alliance for Health Policy and Systems Research. 2015. p. 0–34.
122. Declaration of Alma-Ata 1978 http://www.who.int/publications/almaata_declaration_en.pdf
123. Benger J. General Practitioners and Emergency Departments (GPED): Efficient Models of Care https://www.journalslibrary.nihr.ac.uk/programmes/hsdr/1514506/#/

For peer review only

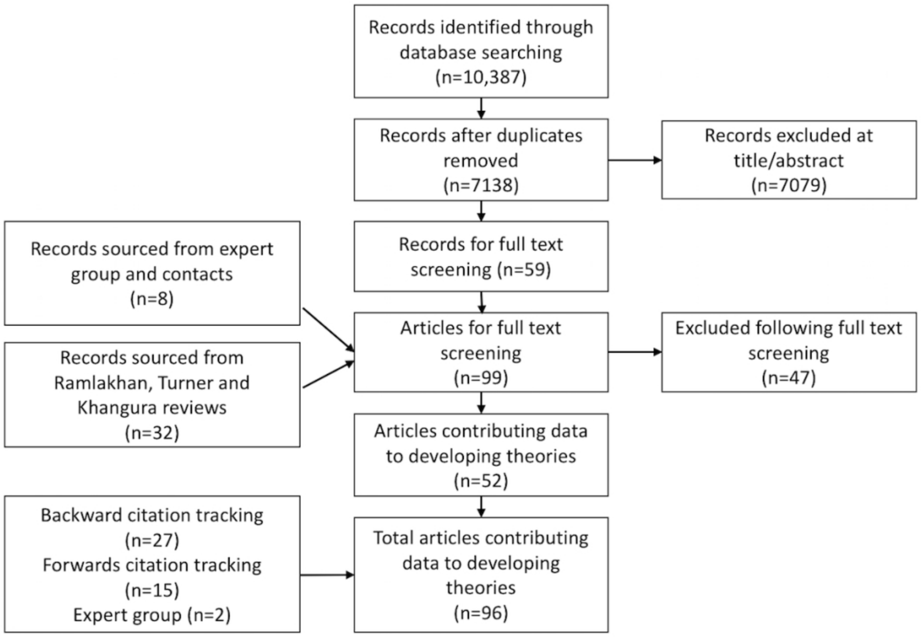

Figure 1: Search strategy and results

112x76mm (300 x 300 DPI)

Supplementary file 1: Search Strategy for Medline (adapted for other databases)

1. exp Primary Health Care/
2. primary care.mp. [mp=title, abstract, original title, name of substance word, subject heading word, keyword heading word, protocol supplementary concept word, rare disease supplementary concept word, unique identifier, synonyms]
3. exp Physicians, Family/
4. exp Physicians, Primary Care/
5. family physician*.mp. [mp=title, abstract, original title, name of substance word, subject heading word, keyword heading word, protocol supplementary concept word, rare disease supplementary concept word, unique identifier, synonyms]
6. exp Family Practice/
7. family practic*.mp. [mp=title, abstract, original title, name of substance word, subject heading word, keyword heading word, protocol supplementary concept word, rare disease supplementary concept word, unique identifier, synonyms]
8. GP.mp. [mp=title, abstract, original title, name of substance word, subject heading word, keyword heading word, protocol supplementary concept word, rare disease supplementary concept word, unique identifier, synonyms]
9. exp After-Hours Care/
10. (after-hours care or out of hours or out-of-hours or OOH).mp. [mp=title, abstract, original title, name of substance word, subject heading word, keyword heading word, protocol supplementary concept word, rare disease supplementary concept word, unique identifier, synonyms]
11. exp General Practitioners/
12. general practic*.mp. [mp=title, abstract, original title, name of substance word, subject heading word, keyword heading word, protocol supplementary concept word, rare disease supplementary concept word, unique identifier, synonyms]
13. 1 or 2 or 3 or 4 or 5 or 6 or 7 or 8 or 9 or 10 or 11 or 12
14. exp Emergency Medical Services/
15. exp Emergency Service, Hospital/
16. emergency department*.mp. [mp=title, abstract, original title, name of substance word, subject heading word, keyword heading word, protocol supplementary concept word, rare disease supplementary concept word, unique identifier, synonyms]
17. (accident and emergenc*).mp. [mp=title, abstract, original title, name of substance word, subject heading word, keyword heading word, protocol supplementary concept word, rare disease supplementary concept word, unique identifier, synonyms]
18. casualt*.mp. [mp=title, abstract, original title, name of substance word, subject heading word, keyword heading word, protocol supplementary concept word, rare disease supplementary concept word, unique identifier, synonyms]

19. emergency room.mp. [mp=title, abstract, original title, name of substance word, subject heading word, keyword heading word, protocol supplementary concept word, rare disease supplementary concept word, unique identifier, synonyms]

20. A&E.mp. [mp=title, abstract, original title, name of substance word, subject heading word, keyword heading word, protocol supplementary concept word, rare disease supplementary concept word, unique identifier, synonyms]

21. urgent care centre*.mp. [mp=title, abstract, original title, name of substance word, subject heading word, keyword heading word, protocol supplementary concept word, rare disease supplementary concept word, unique identifier, synonyms]

22. (walkin or walk in or walk-in).mp. [mp=title, abstract, original title, name of substance word, subject heading word, keyword heading word, protocol supplementary concept word, rare disease supplementary concept word, unique identifier, synonyms]

23. 14 or 15 or 16 or 17 or 18 or 19 or 20 or 21 or 22

24. 13 and 23

25. limit 24 to yr="2015 -Current"

review only

Supplementary file 2: Articles that contributed data to theory development (listed chronologically by Country)

Lead author	Country	Title	Study Design
Ablard 2017	UK	Primary care services co-located with Emergency Departments across a UK region: early views on their development	Survey and semi-structured interviews
Bentley 2017	UK	Emergency Department redirection to primary care: a prospective evaluation of practice	Prospective evaluation of the subsequent management and outcome of redirected non-urgent patients from a Scottish ED over 2 months
Dale 2017	UK	Extended training to prepare GPs for future workforce needs: a qualitative investigation of a one-year fellowship in urgent care	Qualitative investigation of a one-year fellowship in urgent care
Tammes 2016	UK	Exploring the relationship between general practice characteristics, and attendance at walk-in centres, minor injuries units and EDs in England 2012/2013: a cross-sectional study	Cross-sectional observational large data analysed using multivariable regression models
Proctor 2016	UK	A&E Avoidance schemes across London: A rapid review of good practice examples	NHS report - 2 case studies involving redirection of non-urgent patients from the ED
Smith 2016	UK	To GP or not TO GP: Evaluation of children triaged to see a GP in a tertiary paediatric emergency department	Retrospective cohort study of children classified as 'GP appropriate' seen by a GP between 14:00 and 22:00 and seen by ED staff outside these hours
Gnani 2016	UK	Healthcare use among preschool children attending GP-led urgent care centres: a descriptive observational study	Retrospective observational study using routinely collected data
O'Cathain 2016	UK	Variation in avoidable emergency admissions: multiple case studies of emergency and urgent care systems	Ethnographic residual analysis. Interviews with members of emergency care teams at 6 case study sites

For peer review only

Begum 2016	UK	Solving the A&E crisis using GP lead triage and redirection	Poster - Patient questionnaire of 150 patients over 5 weeks redirected back to the GP for treatment with an appointment made on the same day
Gritz 2016	UK	More under fives now seen in urgent care centre than A&E should we shift our focus	Observational - retrospective analysis of routine operational data for attendances
Greenfield 2016	UK	Staff perceptions on patient motives for attending GP-led urgent care centres in London: a qualitative study	Semi-structured interviews
Cowling 2015	UK	Referral outcomes of attendances at general practitioner-led urgent care centres in London, England: retrospective analysis of hospital administrative data	Retrospective analysis of administrative data recorded at a London urgent care centre of 243042 attendances from October 2009 to December 2012
Morton 2016	UK	Describing team development within a novel GP-led urgent care centre model: a qualitative study	Staff semi-structured interviews at 2 GP-led urgent care centres in 2 London academic teaching hospitals
Arain 2015a	UK	Perceptions of healthcare professionals and managers regarding the effectiveness of GP-led walk-in centres in the UK	Qualitative using a phenomenological approach using semi structured interviews
Arain 2015b	UK	Impact of a GP-led walk-in centre on NHS emergency departments	Patient survey over a 3-week period and analysis of attendances at the local children's hospital and minor injuries unit a year before and after the WIC opened
Johnson 2015	UK	Evidence of primary care services at A&E	Letter (opinion piece) Provider of 4 UCC in London supporting co-located GPs services with emergency departments

For peer review only

NHS ECIST 2015	UK	Primary care in emergency departments: a guide to good practice	NHS policy document - Overview of factors to be considered when planning how best to use primary care clinicians in emergency departments, monitoring and refining the service
Harris 2014	UK	How do clinicians with different training backgrounds manage walk-in patients in the ED setting?	Retrospective case note review of a random sample of 384 patients that self-presented to the ED and were initially assessed by GPs or ED staff
Thompson 2013	UK	Suitability of emergency department attenders to be assessed in primary care: survey of general practitioner agreement in a random sample of triage records analysed in a service evaluation project	Four GPs independently used data extracted from 765 clinical notes to rate the appropriateness for management in primary care
Arain 2013	UK	Patients' experience and satisfaction with GP led walk-in centres in the UK; a cross sectional study.	Patient survey over 3 weeks in 2 GP-led WICs
Hunter 2013	UK	A qualitative study of patient choices in using emergency health care for long-term conditions: The importance of candidacy and recursivity.	Questionnaire and semi-structured interviews
Lengu 2012	UK	Application of simulation and modelling in managing unplanned healthcare demand	Conference paper - Simulation and modelling to assess the impact of primary care clinicians deflecting patients with non-urgent needs away from A&E
Carson 2010	UK	Primary care and emergency departments	Report based on results of a literature review, web-based survey and ED visits
Clancy 2009	UK	Launching a social enterprise see-and-treat service	Report outlining the service, number of patients seen and referred on in a 4-month period
Maheswaren 2009	UK	Repeat attenders at national health service walk in centres	Descriptive study using routine data from 4 walk-in centres in England

For peer review only

Sandhu 2009	UK	Emergency nurse practitioners and doctors consulting with patients in an emergency department: a comparison of communication skills and satisfaction	Observation study with a stratified sample of 296 video-taped consultations
Dale 2008	UK	The patient, the doctor and the emergency department: A cross-sectional study of patient-centeredness in 1990 and 2005	Observational study with a stratified sample of 430 video-taped consultations with data collection in May–July 1990 and May–July 2005.
Salisbury 2007	UK	The impact of co-located NHS walk-in centres on emergency departments	Controlled before and after study
Chalder 2007	UK	Comparing care at walk-in centres and at accident and emergency departments: an exploration of patient choice, preference and satisfaction	A controlled, mixed-method study comparing 8 EDs with co-located WICs with the same number of ''traditional'' EDs.
Pope 2005	UK	What do other local providers think of NHS walk-in centres? Results of a postal survey	Postal survey
Bickerton 2005	UK	Streaming A&E patients to walk-in centre services	Analysis of all patients attending a London hospital over 24 hours for suitability for WIC treatment
Chew-Graham 2004	UK	A new role for the general practitioners? Reframing inappropriate attenders to inappropriate services	Qualitative semi-structured staff interviews
Hsu 2003	UK	Effect of NHS walk-in centre on local primary healthcare services	Before and after observational study of consultation rate in 12 general practices after the implementation of a walk-in centre
Salisbury 2002	UK	What is the role of walk-in centres in the NHS?	Analysis of routinely collected data, questionnaire completed by managers followed by semi-structured interviews and site visits
Grant 2002	UK	An observational study comparing quality of care in walk-in centres with general practice and NHS Direct using standardised patients	Observational study involving assessment of clinicians by standardised patients at 20 walk in centres, 20 general practices ad 11 NHS direct sites

For peer review only

Coleman 2001	UK	Will alternative immediate care services reduce demands for non-urgent treatment at accident and emergency?	Questionnaire survey and notes review of non-urgent patients to assess the suitability of management by an alternative care service
McGugan 2000	UK	Primary care or A&E?	Prospective study over 2 months of a redirection policy
Rajpar 2000	UK	Study of choice between accident and emergency departments and general practice centres for out of hours primary care problems	Interview of patients attending A&E and GP out-of-hours
Freeman 1999	UK	Primary care units in A and E departments in North Thames in the 1990s: Initial experience and future implications	Postal questionnaire to ED staff and local GPs with follow up staff interviews
Dale 1998	UK	Primary care in accident and emergency departments: the cost effectiveness and applicability of a new model of care	PhD thesis – Includes data for included papers and additional analysis of 163 video-taped consultations
Ward 1996	UK	Primary care in London: an evaluation of general practitioners working in an inner-city accident and emergency department	Prospective survey over 6 weeks
Dale 1996	UK	Cost effectiveness of treating primary care patients in accident and emergency: a comparison between GPs, senior house officers and registrars	Prospective intervention study which was retrospectively costed
Dale 1995a	UK	Primary care in the accident and emergency department I: Prospective identification of patients	1 year prospective study at a London ED to compare patient characteristics and consultation activities for attenders assessed by nurse triage to have 'primary care' or 'accident and emergency' type problems
Dale 1995b	UK	Primary care in the accident and emergency department: II. Comparison of general practitioners and hospital doctors	1 year prospective study at a London ED to compare patient characteristics and consultation activities for attenders assessed by nurse triage to have 'primary care' or 'accident and emergency' type problems

For peer review only - http://bmjopen.bmj.com/site/about/guidelines.xhtml

O'Kelly 2010	Ireland	Impact of a GP cooperative on lower acuity emergency department attendances	A retrospective review of all attendances at the 'Dubdoc' service was compared with attendances at the ED for triage categories 4 and 5 of the same hospital over a 9-year period
Murphy 2000	Ireland	Effect of patients seeing a general practitioner in accident and emergency on their subsequent attendance: cohort study	Analysis of reattendance of non-urgent patients that had been allocated to general practitioners or usual accident and emergency staff depending on time of registration
Gibney 1999	Ireland	Randomized controlled trial of general practitioner versus usual medical care in a suburban accident and emergency department using an informal triage system	Patients 'randomised' at time of registration to either GP or ED care. Case note review
Murphy 1996	Ireland	Randomised controlled trial of general practitioner versus usual medical care in an urban accident and emergency department: process, outcome and comparative cost	Randomised controlled trial of care provided by general practitioners to non-emergency patients in an accident and emergency department differs significantly from care by usual emergency staff in terms of process, outcome ad cost
van Veelen 2016	Netherlands	Effects of a general practitioner cooperative co-located with an emergency department on patient throughput	Pre-post comparison before and after implementation of a GP cooperative at an ED
Schols 2016	Netherlands	Access to diagnostic tests during GP out-of -hours care: A cross sectional study of all GP out-hours services in the Netherlands	Cross-sectional survey of all 117 GP out of hours services in the Netherlands
Van-Gils-van Rooij 2016	Netherlands	Is patient flow more efficient in urgent care collaborations?	Observational study, compared usual care with UCCs (single point of access for ED and GP OOH)

For peer review only

van Gils-van Rooij 2015	Netherlands	Out-of-Hours Care Collaboration between General Practitioners and Hospital Emergency Departments in the Netherlands	Observational study - comparing attendance and patient characteristics between EDs with standard care and EDs with co-located primary care and single joint triage
Thijssen 2013	Netherlands	The impact on emergency department utilization and patient flows after integrating with a general practitioner cooperative: an observational study	Observational study - routinely collected data over 6 years
Huibers 2013	Netherlands	GP cooperative and emergency department: an exploration of patient flows	Retrospective record review of patients who had visited GPC or ED
Van der Straten 2012	Netherlands	Safety and efficiency of triaging low urgent self-referred patients to a general practitioner at an acute care post: an observational study	Prospective observational study
Bosmans 2012	Netherlands	Addition of a general practitioner to the accident and emergency department: a cost-effective innovation in emergency care	Observational study before and after implementation of new service
Van Veen 2012	Netherlands	Van Veen referral of non-urgent children from the emergency department to general practice: compliance and cost savings	Prospective observational before after study
Van Veen 2011	Netherlands	Safety of the Manchester Triage System to identify less urgent patients in paediatric emergency care: a prospective observational study	Analysis of the hospitalisation rate of self referred children triaged as non-urgent
Boeke 2010	Netherlands	Effectiveness of GPs in accident and emergency departments	Observational study before and after implementation of new service
Kool 2008	Netherlands	Towards integration of general practitioner posts and accident and emergency departments: a case study of two integrated emergency posts in the Netherlands	Observational study comparing contacts, patient satisfaction and staff satisfaction pre-and post set up of a 2 co-located GP OOHs and 2 control sites

For peer review only

Giesen 2006	Netherlands	Patients either contacting a general practice cooperative or accident and emergency department out of hours: a comparison	Retrospective record review
Van Uden 2006	Netherlands	Out-of-hours primary care. Implications of organisation on costs	Annual reports of 2 GP co-operatives (1 co-located, 1 separate) analysed together with ED costs
Van Uden 2005	Netherlands	The Impact of a Primary Care Physician Cooperative on the Caseload of an Emergency Department: The Maastricht Integrated Out-of-Hours Service	Observational study, patient characteristics collected for 3 weeks in Jan/Fen 1998 and March 2001 (co-operative set up in 2000)
Van Uden 2004	Netherlands	Does setting up out of hours primary care cooperatives outside a hospital reduce demand for emergency care?	Before and after observational study
Van Uden 2003	Netherlands	Use of out of hours services: a comparison between two organisations	Observational study of patient contacts at 2 different OOH centres and their associated EDs (1 co-located, 1 not)
Colliers 2017	Belgium	Implementation of a general practitioner cooperative adjacent to the emergency department of a hospital increases the caseload for the GPC but not for the emergency department	Quasi-experimental study analysing the implementation of 2 out of hours general practitioner co-operatives one adjacent to the ED, the other not and 2 control sites
Van den Heede 2016	Belgium	The 2016 proposal for the reorganisation of urgent care provision in Belgium: A political struggle to co-locate primary care providers and emergency departments	Outline of the 2016 political proposal for the reorganisation of urgent care provision toned down due to GP opposition
Ellbrant 2015	Sweden	Paediatric emergency department management benefits from appropriate early redirection of non-urgent visits	Prospective observational study using ED records and case notes
Krakau 1999	Sweden	Provision for clinic patients in the ED produces more nonemergency visits	Before and after observational study

Hansagi 1987	Sweden	Trial of a method of reducing inappropriate demands on a hospital emergency department.	Prospective observational study of 454 patients classified as non-urgent by the ED and redirected to alternative care over a 3-month period
Chmiel 2016	Switzerland	Implementation of a hospital-integrated general practice – a successful way to reduce the burden of inappropriate emergency-department use	Longitudinal observational study
Hess 2015	Switzerland	Satisfaction of health professionals after implementation of a primary care hospital emergency centre in Switzerland: A prospective before-after study	Questionnaire study of job satisfaction before and after a new emergency care model was implemented in Switzerland
Wang 2014	Switzerland	Hospital integrated general practice: a promising way to manage walk in patients	Pre and post comparison study before and after implementation of a new hospital-integrated general practice model
Chmiel 2011	Switzerland	Walk-ins seeking treatment at an emergency department or general practitioner out-of-hours service: a cross-sectional comparison	Analysis of routinely collected data of 2974 patient encounters attending a GPC or ED
Posocco 2017	Italy	Role of out of hours primary care service in limiting inappropriate access to emergency department	Retrospective analysis of 408 ED referrals from a local OOH service
Kork 2016	Finland	Improving access and managing healthcare demand with walk in clinic: convenient but at what cost?	Observational study over 48 months of the characteristics of 107 frequent attenders at a WIC from electronic patient records
Allen 2015	Australia	Low acuity and general practice type presentations to emergency departments: A rural perspective	Analysis of GP type presentations to 2 rural EDs over a 4-month period
Desborough 2013	Australia	Development and implementation of a nurse-led walk-in centre: evidence lost in translation?	Evaluation of the first 12 months of operation of the first Australian public nurse-led primary care walk-in centre compared to the English NHS model.

For peer review only

Nagree 2013	Australia	Quantifying the proportion of general practice and low-acuity patients in the emergency department	Four methods for calculating general practice-type patients were compared for 3 tertiary EDs in Perth, Australia in 2009-2011
Sharma 2011	Australia	Impact of co-located general practitioner (GP) clinics and patient choice on duration of wait in the emergency department	Mathematical modelling of wait times using routine ED data
Richardson 2009	Australia	Myths versus facts in emergency department overcrowding and hospital access block.	Report referencing previous work
Bolton 2001	Australia	The reasons for, and lessons learned from, the closure of the Canterbury GP After-Hours Service.	Report describing why a 12-month trial of GP staffed after hours service with an ED was not continued because the opportunity cost was greater than existing alternative services
Doran 2013	USA	An intervention connecting low acuity emergency department patients with primary care: Effect on future primary care linkage	Analysis of primary care follow up of patients presenting to ED assessed to have non-urgent problem and referred to an onsite primary care clinic
Williams 1996	USA	The costs of visits to emergency departments.	Analysis of emergency department charges and costs based on data from 6 community hospitals
Gadomski 1995	USA	Diverting managed care Medicaid patients from pediatric emergency department use.	6-month follow up of Medicaid children with non-emergent conditions not authorised to be seen in the Pediatric Emergency Department by their primary care provider
Derlet 1995	USA	Prospective identification and triage of nonemergency patients out of an emergency department - 5 year study	5 year study to analyse the outcome of adult patients refused care in the ED
Derlet 1992	USA	Triage of patients out of the emergency department: three year experience.	3 year study to analyse the outcome of adult patients refused care in the ED

For peer review only

Birnbaum 1994	USA	Failure to validate a predictive model for refusal of care to emergency-department patients.	Analysis of the outcome of 534 patients that met the pre-established criteria for refusal of care
Lowe 1994	USA	Refusing care to emergency department patients: evaluation of published triage guidelines.	Case note review of 106 patients who would have been refused care according to triage guidelines
Shaw 1990	USA	Indigent children who are denied care in the emergency department.	Six-month prospective study of 588 children denied care in the emergency department
Rivara 1986	USA	Pediatric nurse triage: its efficacy, safety and implications for care.	Evaluation of emergency room triage of 748 children over a 6-week period at a large urban children's hospital that routinely referred outside of the institution for care
Schull 2007	Canada	The Effect of Low-Complexity Patients on Emergency Department Waiting Times	Analysis of 4.1 million patient visits over a 1 year period (2002-3) and 110 EDs of the effect of low-complexity patients on time of physician contact of high complexity patients
Vertesi 2004	Canada	Does the Canadian Emergency Department Triage and Acuity Scale identify non-urgent patients who can be triaged away from the emergency department?	Retrospective database audit in an urban referral hospital ED.
Hutchison 2003	Canada	Patient satisfaction and quality of care in walk-in clinics, family practices and emergency departments: the Ontario Walk-In Clinic Study.	Prospective cohort study of the quality of care of 8 common acute conditions and patient satisfaction
Anantharaman 2008	Singapore	Impact of health care system interventions on emergency department utilisation and overcrowding in Singapore	Retrospective analysis of attendances at six main public EDs over 32 years
Wilson 2005	New Zealand	Co-locating primary care facilities within emergency departments: brilliant innovation or unwelcome intervention into clinical care?	Report reviewing a proposal to co-locate a primary care facility within the local emergency department

For peer review only

For peer review only

Research checklist: RAMESES publication standards for realist syntheses(25)

List of items to be included when reporting a realist synthesis

TITLE			Reported on page
1		In the title, identify the document as a realist synthesis or review -	1
ABSTRACT			
2		While acknowledging publication requirements and house style, abstracts should ideally contain brief details of: the study's background, review question or objectives; search strategy; methods of selection, appraisal, analysis and synthesis of sources; main results; and implications for practice.	2
INTRODUCTION			
3	Rationale for review	Explain why the review is needed and what it is likely to contribute to existing understanding of the topic area.	5
4	Objectives and focus of review	State the objective(s) of the review and/or the review question(s). Define and provide a rationale for the focus of the review.	6
METHODS			
5	Changes in the review process	Any changes made to the review process that was initially planned should be briefly described and justified.	n/a
6	Rationale for using realist synthesis	Explain why realist synthesis was considered the most appropriate method to use.	6-7

For peer review only

TITLE			Reported on page
7	Scoping the literature	Describe and justify the initial process of exploratory scoping of the literature.	8
8	Searching processes	While considering specific requirements of the journal or other publication outlet, state and provide a rationale for how the iterative searching was done. Provide details on all the sources accessed for information in the review. Where searching in electronic databases has taken place, the details should include, for example, name of database, search terms, dates of coverage and date last searched. If individuals familiar with the relevant literature and/or topic area were contacted, indicate how they were identified and selected.	8
9	Selection and appraisal of documents	Explain how judgements were made about including and excluding data from documents, and justify these.	9
10	Data extraction	Describe and explain which data or information were extracted from the included documents and justify this selection.	9
11	Analysis and synthesis processes	Describe the analysis and synthesis processes in detail. This section should include information on the constructs analyzed and describe the analytic process.	9
RESULTS			
12	Document flow diagram	Provide details on the number of documents assessed for eligibility and included in the review with reasons for exclusion at each stage as well as an indication of their	Figure 1

For peer review only

TITLE			Reported on page
		source of origin (for example, from searching databases, reference lists and so on). You may consider using the example templates (which are likely to need modification to suit the data) that are provided.	
13	Document characteristics	Provide information on the characteristics of the documents included in the review.	10<br>And supplementary file 2
14	Main findings	Present the key findings with a specific focus on theory building and testing.	10-23
DISCUSSION			
15	Summary of findings	Summarize the main findings, taking into account the review's objective(s), research question(s), focus and intended audience(s).	24
16	Strengths, limitations and future research directions	Discuss both the strengths of the review and its limitations. These should include (but need not be restricted to) (a) consideration of all the steps in the review process and (b) comment on the overall strength of evidence supporting the explanatory insights which emerged.<br>The limitations identified may point to areas where further work is needed.	24
17	Comparison with existing literature	Where applicable, compare and contrast the review's findings with the existing literature (for example, other reviews) on the same topic.	25

For peer review only

TITLE			Reported on page
18	Conclusion and recommendations	List the main implications of the findings and place these in the context of other relevant literature. If appropriate, offer recommendations for policy and practice.	27
19	Funding	Provide details of funding source (if any) for the review, the role played by the funder (if any) and any conflicts of interests of the reviewers.	28

For peer review only
